# Novel Hybrid Indole-Based Caffeic Acid Amide Derivatives as Potent Free Radical Scavenging Agents: Rational Design, Synthesis, Spectroscopic Characterization, In Silico and In Vitro Investigations

**DOI:** 10.3390/metabo13020141

**Published:** 2023-01-17

**Authors:** Ahmed Elkamhawy, Na Kyoung Oh, Noha A. Gouda, Magda H. Abdellattif, Saud O. Alshammari, Mohammed A. S. Abourehab, Qamar A. Alshammari, Amany Belal, Minkyoung Kim, Ahmed A. Al-Karmalawy, Kyeong Lee

**Affiliations:** 1 BK21 FOUR Team and Integrated Research Institute for Drug Development, College of Pharmacy, Dongguk University—Seoul, Goyang 10326, Republic of Korea; 2 College of Pharmacy, Dongguk University—Seoul, Goyang 10326, Republic of Korea; 3 Department of Pharmaceutical Organic Chemistry, Faculty of Pharmacy, Mansoura University, Mansoura 35516, Egypt; 4 Department of Chemistry, College of Science, Taif University, Turaba Branch P.O. Box 11099, Taif 21944, Saudi Arabia; 5 Department of Plant Chemistry and Natural Products, Faculty of Pharmacy, Northern Border University, Arar 91431, Saudi Arabia; 6 Department of Pharmaceutics, College of Pharmacy, Umm Al-Qura University, Makkah 21955, Saudi Arabia; 7 Department of Pharmacology and Toxicology, Faculty of Pharmacy, Northern Border University, Arar 91431, Saudi Arabia; 8 Department of Pharmaceutical Chemistry, College of Pharmacy, Taif University, P.O. Box 11099, Taif 21944, Saudi Arabia; 9 Medicinal Chemistry Department, Faculty of Pharmacy, Beni-Suef University, Beni-Suef 62514, Egypt; 10 Pharmaceutical Chemistry Department, Faculty of Pharmacy, Ahram Canadian University, 6th of October City, Giza 12566, Egypt

**Keywords:** bioactive molecules, oxidative stress, antioxidant activity, indole, caffeic acid, DPPH, ABTS, ORAC, FRAP, spectroscopic characterization

## Abstract

Antioxidant small molecules can prevent or delay the oxidative damage caused by free radicals. Herein, a structure-based hybridization of two natural antioxidants (caffeic acid and melatonin) afforded a novel hybrid series of indole-based amide analogues which was synthesized with potential antioxidant properties. A multiple-step scheme of in vitro radical scavenging assays was carried out to evaluate the antioxidant activity of the synthesized compounds. The results of the DPPH assay demonstrated that the indole-based caffeic acid amides are more active free radical scavenging agents than their benzamide analogues. Compared to Trolox, a water-soluble analogue of vitamin E, compounds **3a**, **3f**, **3h**, **3j**, and **3m** were found to have excellent DPPH radical scavenging activities with IC_50_ values of 95.81 ± 1.01, 136.8 ± 1.04, 86.77 ± 1.03, 50.98 ± 1.05, and 67.64 ± 1.02 µM. Three compounds out of five (**3f**, **3j**, and **3m**) showed a higher capacity to neutralize the radical cation ABTS^•+^ more than Trolox with IC_50_ values of 14.48 ± 0.68, 19.49 ± 0.54, and 14.92 ± 0.30 µM, respectively. Compound **3j** presented the highest antioxidant activity with a FRAP value of 4774.37 ± 137.20 μM Trolox eq/mM sample. In a similar way to the FRAP assay, the best antioxidant activity against the peroxyl radicals was demonstrated by compound **3j** (10,714.21 ± 817.76 μM Trolox eq/mM sample). Taken together, compound **3j** was validated as a lead hybrid molecule that could be optimized to maximize its antioxidant potency for the treatment of oxidative stress-related diseases.

## 1. Introduction

Since the majority of our biological activities only take place in the presence of oxygen, it is necessary for life. However, the oxidation reaction may lead to cell damage, leading to the degradation of various oxygen substrates, proteins, lipids, and DNA. This may cause numerous diseases including inflammation, obesity, diabetes, arthritis, etc. [1,2]. The oxygen paradox contributes to the generation of free radicals which possess a single electron on an oxygen or nitrogen atom, known as reactive oxygen species (ROS) or reactive nitrogen species (RNS) [3,4]. Free radicals are highly unstable. They could be valuable when they are involved in physiological functions or harmful if there is no balance between the defense systems and ROS/RNS, or when the organism is incapable of restricting the destruction triggered by the free radicals, which is known by the oxidative stress [5].

**Figure 1:**
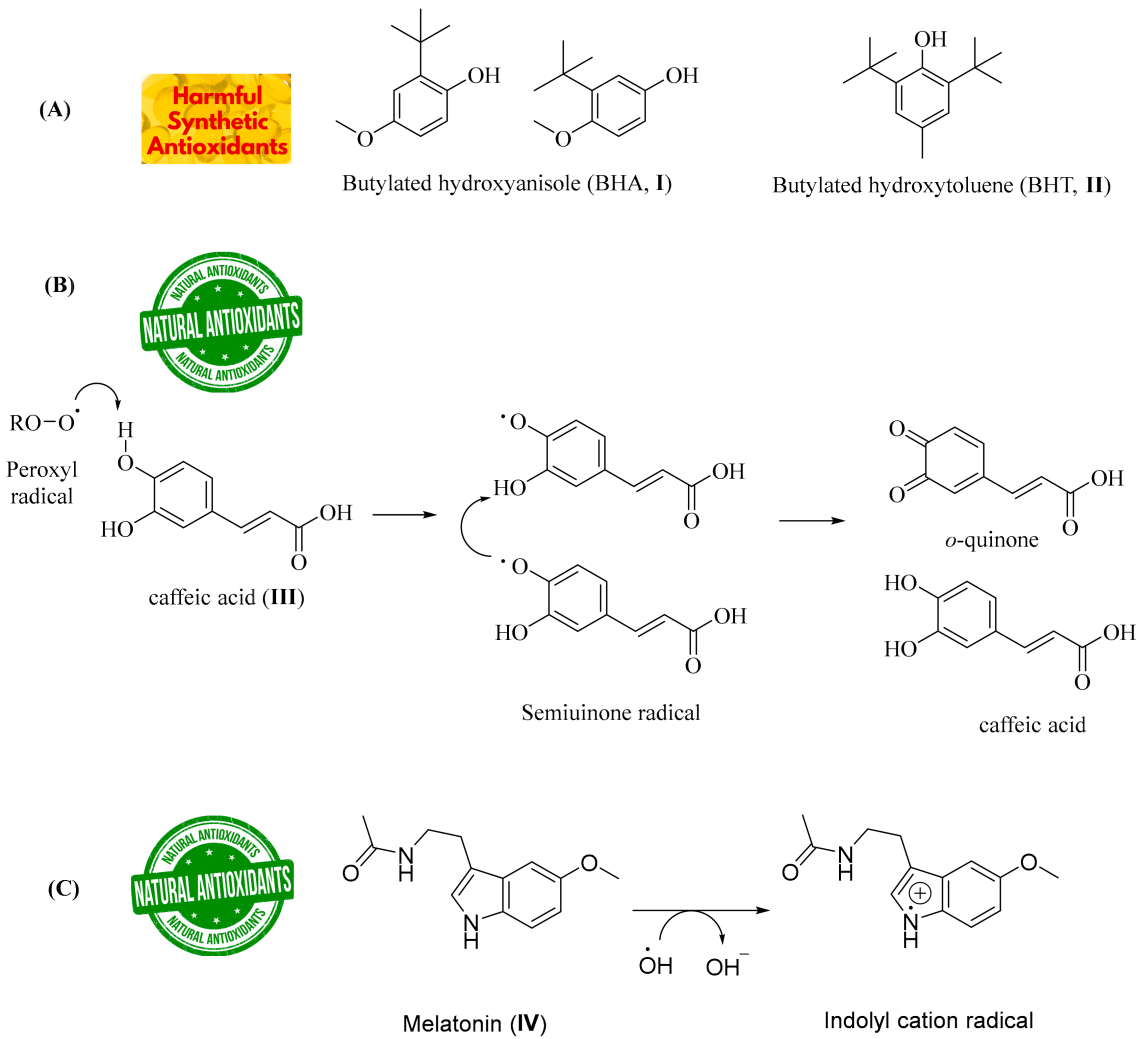
(**A**) Chemical structures of some well-known synthetic antioxidants (BHA; **I** and BHT; **II)**; (**B**) Chemical structure of the natural antioxidant caffeic acid (**III**) and a proposed mechanism for caffeic acid antioxidant activity; (**C**) Chemical structure of the natural antioxidant melatonin (**IV**) and the proposed mechanism of how the electron-rich aromatic indole ring system of melatonin scavenges the hydroxyl radical (HO^•^).

Antioxidants are compounds able to inhibit or postpone the oxidation process via neutralizing free radicals. Some synthetic antioxidants including butylated hydroxyanisole (BHA; **I**, Figure 1A) and butylated hydroxytoluene (BHT; **II**, Figure 1A) have recently been reported to be toxic to the environment and the human health [6,7]. Recently, numerous natural antioxidants’ effectiveness has been reported [8]. Antioxidants, whether they are produced naturally or artificially, reduce the severity of oxidative damage via scavenging ROS or stopping ROS-mediated chain reactions. As a result, finding naturally occurring compounds with antioxidant activity followed by hybridizing these natural antioxidant chemical scaffolds becomes a significant scientific issue with several socioeconomic interests. Despite being found in nature with many health advantages [9], the research being done on hybridizing essential antioxidant pharmacophores is still very important today [10,11,12,13,14]. 

Caffeic acid ((*E*)-3-(3,4-dihydroxyphenyl)prop-2-enoic acid, **III**, Figure 1B) is one of the main hydroxycinnamic acids possessing active antioxidant activity, as sketched in Figure 1B. It was reported that caffeic acid has potent free radical scavenging activities [15]. Melatonin (**IV**, Figure 1C), which is also a remarkable antioxidant natural compound [16,17], can scavenge oxygen free radicals, such as super-oxide radicals, hydroxyl radicals, and others. Along with its antioxidant and neuroprotective activities [18,19,20,21,22,23], melatonin was reported to have a therapeutic potential for inflammation [24], cancer [25], pain [26], cardiovascular disorders, etc. [27,28,29,30,31,32]. Thus, numerous derivatives of melatonin were reported with several biological activities [33,34,35,36,37,38,39,40]. The potent activity of melatonin as an ROS-scavenging agent including ^1^O_2_, O_2_^•−^, H_2_O_2_, hydroxyl radical (HO^•^), and peroxyl radical (ROO^•^) is due to the electron-rich aromatic indole chemical scaffold (Figure 1C), which enables indoleamine to serve as an electron donor, forming an indolyl cation [23,41,42,43]. In this context, various indole derivatives with promising activities against oxidative stress and monoamine oxidase B enzyme (MAO-B) were recently reported by our research group [44,45,46]. 

**Figure 2:**
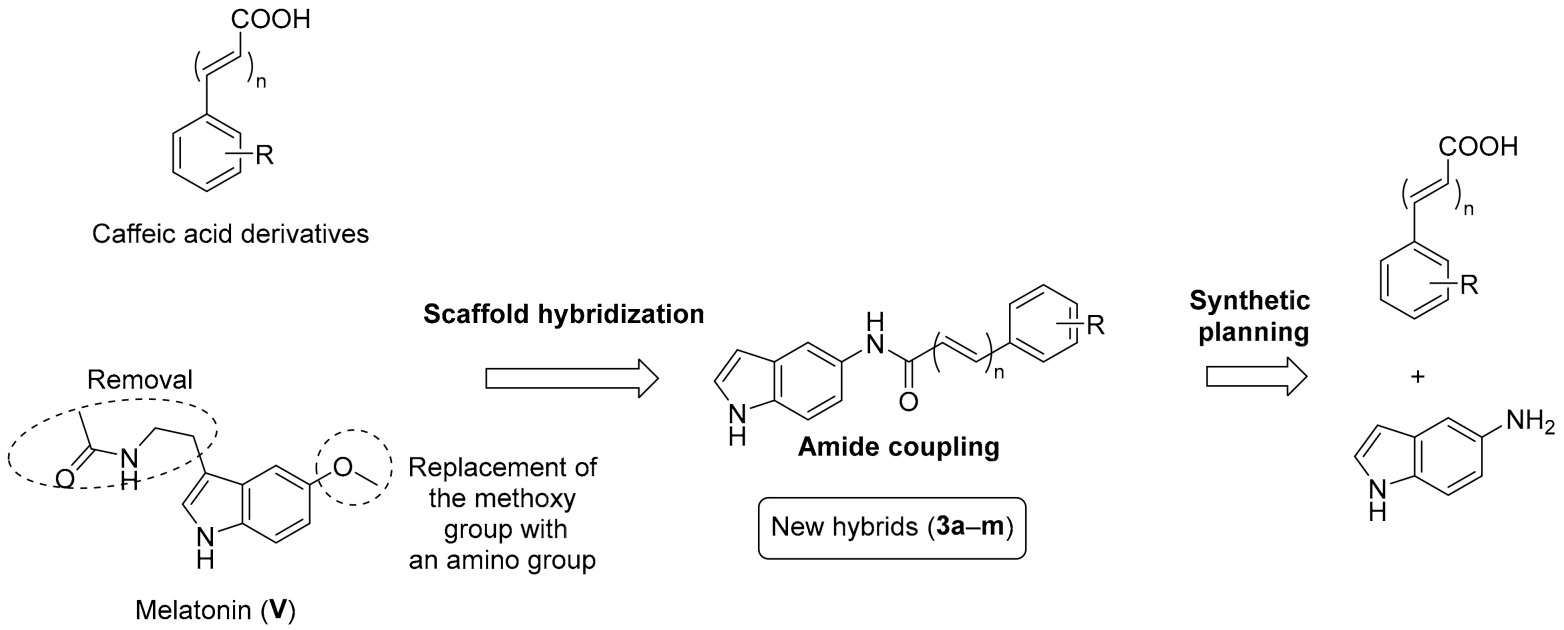
Design of structural hybridization of the caffeic acid derivatives with melatonin scaffold, in addition to the synthetic planning, to obtain the novel indole–caffeic amide analogues **3a**–**m**.

The potent antioxidant activity of both chemical scaffolds (indole and caffeic acid) as shown in Figure 1 encourages our team to design a hybrid scaffold that could have potential antioxidant power. Consequently, development of an efficient indole–caffeic pharmacophore could be enormously important. As illustrated in Figure 2, the methoxy group of melatonin was substituted with an amino group, followed by amide formation by reacting with various caffeic acid analogues to generate the desired amide derivatives (**3a**–**m**). The selection process of the functional groups in compounds **3a**–**m** was inspired by their existence in neuroprotectant compounds which showed powerful activities against oxidative stress [47,48,49]. For this objective, a straightforward synthetic approach was used to synthesize the new hybrid indole–caffeic amide analogues **3a**–**m**, and their therapeutic potential against ROS was preliminary assessed (Figure 2).

## 2. Materials and Methods

### 2.1. Chemical Reagents, Purification, and Instrumentation

The general protocols utilized for the chemical synthesis, structure elucidation, and purity of the newly synthesized indole–caffeic acid hybrids are provided in the Supplementary File. 

### 2.2. Synthesis of Indole–caffeic Amide Analogues **3a**–**m**

5-Aminoindole (**1**, 0.1 g, 0.75 mmol), 1-ethyl-3-(3-dimethylaminopropyl)carbodiimide (EDCI, 0.21 g, 1.1 mmol), and hydroxybenzotriazole (HOBt, 0.14 g, 1.1 mmol) were mixed in the presence of *N*,*N*-diisopropylethylamine (DIPEA, 0.19 mL, 1.1 mmol) and acetonitrile solvent (5 mL). The appropriate carboxylic acid reagent (**2**, 0.75 mmol, 1 eq.) was then added. The reaction was carried out at room temperature (25 °C) for 2 h. The excess acetonitrile was evaporated. Work-up was performed using ethyl acetate (EA) and water. The organic solution was evaporated, dried, and purified via flash column chromatography (SiO_2_, *n*-hexane:EA = 10:1) to obtain the indole–caffeic amide derivatives in suitable yields (Table 1).

#### 2.2.1. 4-Amino-3-hydroxy-*N*-(1*H*-indol-5-yl)benzamide (**3a**)

Yellowish-white solid. M.p.: 182–183 °C. HPLC purity: 5.614 min, 96.71%. ^1^H NMR (400 MHz, DMSO-*d_6_*) *δ* 10.96 (s, 1H), 9.58 (s, 1H), 9.24 (s, 1H), 7.93 (s, 1H), 7.36–7.29 (m, 5H), 6.62 (d, *J* = 8.00 Hz, 1H), 6.37 (s, 1H), 5.09 (br, 2H). ^13^C NMR (100 MHz, DMSO-*d*_6_) *δ* 165.59, 143.47, 140.75, 133.08, 131.98, 127.80, 126.06, 123.17, 120.17, 116.54, 114.44, 112.88, 112.27, 111.23, 102.00. HRMS (ESI) *m/z* calculated for C_15_H_14_N_3_O_2_ [M+H]^+^: 268.1086, found: 268.1076. 

#### 2.2.2. 2-Bromo-*N*-(1*H*-indol-5-yl)-4,5-dimethoxybenzamide (**3b**)

White solid. M.p.: 256–257 °C. HPLC purity: 11.235 min, 99.73%. ^1^H NMR (400 MHz, DMSO-*d_6_*) *δ* 11.02 (s, 1H), 10.09 (s, 1H), 7.99 (s, 1H), 7.33–7.31 (m, 3H), 7.21 (s, 1H), 7.14 (s, 1H), 6.60 (m, 1H), 3.82 (s, 6H). ^13^C NMR (100 MHz, DMSO-*d*_6_) *δ* 165.55, 150.40, 148.38, 133.33, 132.04, 131.51, 127.82, 126.35, 116.90, 115.60, 112.65, 111.59, 111.49, 110.08, 101.57, 55.57. HRMS (ESI) *m/z* calculated for C_17_H_16_BrN_2_O_3_ [M+H]^+^: 375.0344, found: 375.0331.

#### 2.2.3. 4-Bromo-3,5-dihydroxy-*N*-(1*H*-indol-5-yl)benzamide (**3c**)

Yellow solid. M.p.: 211–212 °C. HPLC purity: 7.414 min, 99.60%. ^1^H NMR (400 MHz, DMSO-*d_6_*) *δ* 11.01 (s, 1H), 10.29 (br, 2H), 10.03 (s, 1H), 7.95 (s, 1H), 7.35–7.32 (m, 3H), 6.93 (s, 2H), 6.40 (s, 1H). ^13^C NMR (100 MHz, DMSO-*d*_6_) *δ* 165.57, 155.60, 136.38, 133.35, 131.41, 127.80, 126.32, 116.28, 112.36, 111.44, 106.41, 101.57, 101.37. HRMS (ESI) *m/z* calculated for C_15_H_12_BrN_2_O_3_ [M+H]^+^: 347.0031, found: 347.0018. 

#### 2.2.4. 3-Amino-4-hydroxy-*N*-(1*H*-indol-5-yl)benzamide (**3d**)

Orange solid. M.p.: 182–183 °C. HPLC purity: 12.966 min, 99.87%. ^1^H NMR (400 MHz, DMSO-*d_6_*) *δ* 11.63 (br, 1H), 11.04 (s, 1H), 10.15 (s, 1H), 8.57 (s, 1H), 8.17 (d, *J* = 8.00 Hz, 1H), 7.95 (s, 1H), 7.36–7.32 (m, 3H), 7.24 (d, *J* = 8.00 Hz, 1H), 6.41 (s, 1H). ^13^C NMR (100 MHz, DMSO-*d*_6_) *δ* 163.15, 154.68, 136.93, 134.60, 133.48, 131.08, 127.82, 126.45, 126.37, 125.25, 119.31, 116.54, 112.78, 111.47, 101.59. HRMS (ESI) *m/z* calculated for C_15_H_14_N_3_O_2_ [M+H]^+^: 268.1086, found: 268.1094.

#### 2.2.5. (*E*)-3-(4-Hydroxyphenyl)-*N*-(1*H*-indol-5-yl)acrylamide (**3e**)

White solid. M.p.: 153–154 °C. HPLC purity: 8.983 min, 96.06%. ^1^H NMR (400 MHz, DMSO-*d_6_*) *δ* 10.98 (s, 1H), 9.85 (br, 2H), 7.99 (s, 1H), 7.47–7.43 (m, 3H), 7.33–7.27 (m, 3H), 6.82 (d, *J* = 8.00 Hz, 2H), 6.64 (d, *J* = 16.00 Hz, 1H), 6.38 (s, 1H). ^13^C NMR (100 MHz, DMSO-*d*_6_) *δ* 163.98, 159.32, 139.72, 133.10, 131.92, 129.77, 127.98, 126.38, 126.30, 119.73, 116.22, 115.15, 111.65, 110.93, 101.57. HRMS (ESI) *m/z* calculated for C_17_H_15_N_2_O_2_ [M+H]^+^: 279.1134, found: 279.1126.

#### 2.2.6. (*E*)-3-(4-Hydroxy-3-methoxyphenyl)-*N*-(1*H*-indol-5-yl)acrylamide (**3f**)

Yellowish-white solid. M.p.: 177–178 °C. HPLC purity: 9.259 min, 97.06%. ^1^H NMR (400 MHz, DMSO-*d_6_*) *δ* 10.98 (s, 1H), 9.86 (s, 1H), 9.44 (s, 1H), 8.00 (s, 1H), 7.45 (d, *J* = 12.00 Hz, 1H), 7.33–7.30 (m, 3H), 7.18 (s, 1H), 7.05 (d, *J* = 8.00 Hz, 1H), 6.82 (d, *J* = 8.00 Hz, 1H), 6.67 (d, *J* = 12.00 Hz, 1H), 6.38 (s, 1H), 3.83 (s, 3H). ^13^C NMR (100 MHz, DMSO-*d*_6_) *δ* 163.92, 148.82, 148.29, 140.02, 133.09, 131.95, 127.94, 126.88, 126.30, 122.11, 120.01, 116.17, 115.09, 111.65, 111.22, 110.93, 101.36, 55.93. HRMS (ESI) *m/z* calculated for C_18_H_17_N_2_O_3_ [M+H]^+^: 309.1239, found: 309.1234.

#### 2.2.7. 4-Hydroxy-*N*-(1*H*-indol-5-yl)-3,5-dimethoxybenzamide (**3g**)

White solid. M.p.: 198–199 °C. HPLC purity: 7.354 min, 97.64%. ^1^H NMR (400 MHz, DMSO-*d_6_*) *δ* 11.01 (s, 1H), 9.82 (s, 1H), 8.93 (s, 1H), 7.89 (s, 1H), 7.35–7.31 (m, 5H), 6.40 (s, 1H), 3.85 (s, 6H). ^13^C NMR (100 MHz, DMSO-*d*_6_) *δ* 165.01, 147.86, 139.08, 133.39, 131.36, 127.84, 126.29, 125.32, 116.94, 113.03, 111.38, 105.83, 101.52, 56.55. HRMS (ESI) *m/z* calculated for C_17_H_17_N_2_O_4_ [M+H]^+^: 313.1188, found: 313.1178.

#### 2.2.8. 3,4-Dihydroxy-*N*-(1*H*-indol-5-yl)benzamide (**3h**)

White solid. M.p.: 145–146 °C. HPLC purity: 5.485 min, 99.53%. ^1^H NMR (400 MHz, DMSO-*d_6_*) *δ* 10.98 (s, 1H), 9.75 (s, 1H), 9.35 (br, 2H), 7.94 (s, 1H), 7.40–7.30 (m, 5H), 6.81 (d, *J* = 8.00 Hz, 1H), 5.38 (s, 1H). ^13^C NMR (100 MHz, DMSO-*d*_6_) *δ* 165.31, 148.86, 145.29, 133.22, 131.71, 127.80, 126.94, 126.18, 119.78, 116.56, 115.81, 115.28, 112.46, 111.31, 101.50. HRMS (ESI) *m/z* calculated for C_15_H_13_N_2_O_3_ [M+H]^+^: 269.0926, found: 269.0913. 

#### 2.2.9. 4-Hydroxy-*N*-(1*H*-indol-5-yl)-2-methylbenzamide (**3i**)

White solid. M.p.: 180–181 °C. HPLC purity: 7.529 min, 98.76%. ^1^H NMR (400 MHz, DMSO-*d_6_*) *δ* 10.97 (s, 1H), 9.82 (s, 1H), 9.65 (s, 1H), 7.97 (s, 1H), 7.34–7.29 (m, 4H), 6.66–6.64 (m, 2H), 6.37 (s, 1H), 2.34 (s, 3H). ^13^C NMR (100 MHz, DMSO-*d*_6_) *δ* 167.78, 158.70, 138.10, 133.15, 131.96, 129.63, 128.97, 127.82, 126.20, 117.59, 115.79, 112.52, 111.56, 111.38, 101.50, 20.29. HRMS (ESI) *m/z* calculated for C_16_H_15_N_2_O_2_ [M+H]^+^: 267.1134, found: 267.1121.

#### 2.2.10. (*E*)-3-(3,4-Dihydroxyphenyl)-*N*-(1*H*-indol-5-yl)acrylamide (**3j**)

Yellow solid. M.p.: 171–172 °C. HPLC purity: 7.262 min, 99.59%. ^1^H NMR (400 MHz, DMSO-*d_6_*) *δ* 10.99 (s, 1H), 9.87 (s, 1H), 9.28 (br, 2H), 8.00 (s, 1H), 7.39–7.30 (m, 4H), 7.01 (s, 1H), 6.90 (d, *J* = 8.00 Hz, 1H), 6.78 (d, *J* = 8.00 Hz, 1H), 6.58 (d, *J* = 12.00 Hz, 1H), 6.38 (d, *J* = 4.00 Hz, 1H). ^13^C NMR (100 MHz, DMSO-*d*_6_) *δ* 163.95, 147.88, 146.01, 140.11, 133.08, 131.92, 127.93, 126.87, 126.28, 121.08, 119.51, 116.27, 115.13, 114.26, 111.64, 110.91, 101.56. HRMS (ESI) *m/z* calculated for C_17_H_15_N_2_O_3_ [M+H]^+^: 295.1083, found: 295.1070.

#### 2.2.11. 4-Hydroxy-*N*-(1*H*-indol-5-yl)benzamide (**3k**)

White solid. M.p.: 168–169 °C. HPLC purity: 6.908 min, 98.94%. ^1^H NMR (400 MHz, DMSO-*d_6_*) *δ* 10.99 (s, 1H), 10.01 (s, 1H), 9.81 (s, 1H), 7.95 (s, 1H), 7.87 (d, *J* = 8.00 Hz, 2H), 7.36–7.31 (m, 3H), 6.86 (d, *J* = 8.00 Hz, 2H), 6.39 (s, 1H). ^13^C NMR (100 MHz, DMSO-*d*_6_) *δ* 165.17, 160.61, 133.28, 131.62, 129.92, 127.83, 126.39, 126.19, 116.64, 115.23, 112.57, 111.35, 101.54. HRMS (ESI) *m/z* calculated for C_15_H_13_N_2_O_2_ [M+H]^+^: 253.0977, found: 253.0966.

#### 2.2.12. 4-Hydroxy-*N*-(1*H*-indol-5-yl)-3-methoxybenzamide (**3l**)

White solid. M.p.: 190–191 °C. HPLC purity: 7.301 min, 99.75%. ^1^H NMR (400 MHz, DMSO-*d_6_*) *δ* 11.01 (s, 1H), 9.83 (s, 1H), 9.60 (s, 1H), 7.93 (s, 1H), 7.56 (d, *J* = 4.00 Hz, 1H), 7.51 (dd, *J* = 8.0, 4.0 Hz, 1H), 7.35–7.31 (m, 3H), 6.87 (d, *J* = 8.00 Hz, 1H), 6.40 (s, 1H), 3.86 (s, 3H). ^13^C NMR (100 MHz, DMSO-*d*_6_) *δ* 165.10, 150.00, 147.58, 133.33, 131.51, 127.84, 126.64, 126.22, 121.56, 116.80, 115.22, 122.78, 112.03, 111.37, 101.53, 56.12. HRMS (ESI) *m/z* calculated for C_16_H_15_N_2_O_3_ [M+H]^+^: 283.1083, found: 283.1073.

#### 2.2.13. (*E*)-3-(4-Hydroxy-3,5-dimethoxyphenyl)-*N*-(1*H*-indol-5-yl)acrylamide (**3m**)

Yellow solid. M.p.: 199–200 °C. HPLC purity: 8.923 min, 99.88%. ^1^H NMR (400 MHz, DMSO-*d_6_*) *δ* 11.00 (s, 1H), 9.90 (s, 1H), 8.84 (s, 1H), 8.03 (s, 1H), 7.48 (d, *J* = 16.00 Hz, 1H), 7.35–7.30 (m, 3H), 6.92 (s, 2H), 6.71 (d, *J* = 16.00 Hz, 1H), 6.39 (s, 1H), 3.83 (s, 6H). ^13^C NMR (100 MHz, DMSO-*d*_6_) *δ* 163.84, 148.52, 140.30, 137.82, 133.09, 131.98, 127.94, 126.31, 125.74, 120.36, 115.04, 111.66, 110.82, 105.67, 101.56, 56.37. HRMS (ESI) *m/z* calculated for C_19_H_19_N_2_O_4_ [M+H]^+^: 339.1345, found: 339.1340.

### 2.3. In Vitro Antioxidant Assays

#### 2.3.1. 2,2-Diphenyl-1-picrylhydrazyl Radical-Scavenging Activity (DPPH Assay) 

Final concentrations of 20 mM of the tested compounds and 0.1 mM DMSO were prepared to determine the range of the inhibitory concentration 50 (EC_50_). A solution of Trolox (a water-soluble analogue of vitamin E, 1000 μM) was prepared in DMSO, from which 5 final concentrations were prepared including 5, 10, 20, 40, and 80 μM. DPPH (2,2-diphenyl-1-picryl-hydrazyl-hydrate) free radical assay was performed as reported [50]. Further details were provided in the Supplementary Materials.

#### 2.3.2. 2,2’-Azino-bis(3-ethylbenzothiazoline-6-sulfonate (ABTS) Assay 

The assay was performed as reported [51], with minor modifications. For details, please refer to the Supplementary Materials.

#### 2.3.3. Ferric Reducing Power (FRAP) Assay

A stock solution of Trolox (3 mM in methanol) was made, and the following dilutions were prepared at the concentrations of 1500, 1000, 800, 400, 200, 100, and 50 μM. Samples were initially dissolved in DMSO to obtain a 40 mM concentration (depending on the molecular weight of each compound). Then, they were diluted to reach the concentration of 0.2 mM with methanol. The assay was performed as reported [52], with minor modifications. For details, please refer to the Supplementary Materials.

#### 2.3.4. Oxygen Radical Absorbance Capacity (ORAC Assay)

A stock solution of Trolox (2 mM in MeOH) was prepared, and the following dilutions were prepared: 1200, 900, 600, 500, 400, 300, 200, 100, and 50 μM. Samples were initially dissolved in DMSO at concentrations of 40 mM according to the provided molecular weights. Then, samples were diluted with methanol until reaching the concentration of 0.1 mM. The assay was carried out as reported [53], with modifications. Further details are provided in the Supplementary Materials.

## 3. Results and Discussion

### 3.1. Chemical Synthesis

**Scheme 1:**
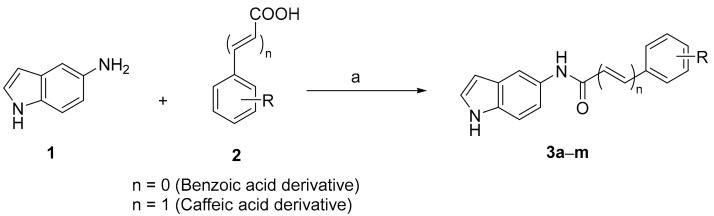
(a) Appropriate carboxylic acid derivative (0.75 mmol), EDCI (1.1 mmol), HOBt (1.1 mmol), DIPEA (1.1 mmol), acetonitrile (5 mL), 25 °C, 12 h. (Full description is indicated in the Materials and Methods section).

As sketched in Scheme 1, a series of indole-based benzamide and caffeic acid amide analogues **3a**–**m** was synthesized. The amide formation reaction was accomplished via reacting 5-aminoindole (**1**) in acetonitrile solvent with a variety of commercially available benzoic or caffeic acid derivatives (**2**). The coupling reagents EDCI and HOBt were used, in addition to DIPEA as an organic base. As shown in Table 1, a variety of indole-based benzamide and caffeic acid amide analogues possessing different chemical substituents were acquired in acceptable yields.

### 3.2. Structure Elucidation of the Newly Synthesized Amide Derivatives **3a–m**

The chemical structures of the newly synthesized indole-based benzamide and caffeic acid amide analogues (**3a**–**m**) were elucidated using different spectroscopic techniques. The purity of compounds **3a**–**m** was found to be more than 96%. The ^1^H NMR spectra of all the synthesized analogues were characterized by two major singlet peaks with high chemical shifts (>9.00 ppm); the proton of the NH group of the indole scaffold and the proton of the amide group (CONH). 

As provided in the Supplementary File, the ^1^H NMR spectrum of the indole-based benzamide analogue **3a** displayed the free para NH_2_ group at 5.09 ppm as a broad peak (br) representing the two protons of the amino group. In addition, the amide carbon (CO) appeared clearly in the ^13^C NMR spectrum of compound **3a** at 165.59 ppm, which confirmed the formation of the amide group. Indeed, ^13^C NMR spectra of all the newly synthesized indole-based benzamide and caffeic acid amide analogues (**3a**–**m**) showed signals resonating around 163.00–168.00 ppm (CO group of the amide moiety). For compound **3b**, the two methoxy groups were found at 3.82 and 55.57 ppm in the ^1^H and ^13^ C NMR, respectively. The ^1^H NMR chart of compound **3c** was characterized by a singlet long aromatic peak at 6.93 ppm representing the two para phenyl protons of the 4-bromo-3,5-dihydroxy benzoyl moiety. In addition, the protons of the two hydroxyl moieties were detected as a singlet broad peak at 10.29 ppm. In its ^13^C NMR chart, compound **3c** showed a long peak at 155.60 attributable to the two carbons carrying the two hydroxyl moieties. The ^1^H NMR spectrum of the benzamide derivative **3d** was characterized presence of three singlet peaks with chemical shifts higher than 9.00 ppm (the indole NH, the amide NH, and the free phenolic OH groups). 

The first synthesized caffeic acid amide ((*E*)-3-(4-hydroxyphenyl)-*N*-(1*H*-indol-5-yl)acrylamide, **3e**) showed these three protons in the range of 9.85–10.98 ppm. In the meantime, one vinylic proton was successfully detected with its characteristic trans *J* coupling constant of 16.00 Hz at 6.64 ppm. The carbon of the amide moiety appeared at 163.98 in the ^13^C NMR spectrum of the caffeic acid amide **3e**, while the carbon holding the free phenolic OH group was detected at 159.32 ppm. Similarly, the ^1^H NMR spectrum confirmed the synthesis and the final chemical structure of the second caffeic acid amide derivative in this series (**3f**) by the presence of three singlet peaks in the range of 9.44–10.98 ppm representing the indole NH, amide NH, and phenolic OH groups, in addition to the three protons of the meta methoxy group (*m*-OCH_3_) in the aliphatic region (3.83 ppm). Its ^13^C NMR chart showed the amide carbon peak at 163.92 ppm, the two carbons bearing the free OH and the methoxy groups at 148.82 and 148.29 ppm, in addition to the aliphatic carbon of the methoxy group at 55.93 ppm. 

The ^1^H NMR spectrum of compound **3g** was characterized by a long peak in the aliphatic region at 3.85 representing the six protons of the two methoxy groups. Meanwhile, its ^13^C NMR chart showed the amide carbon chemical shift at 165.01 ppm, a long peak at 147.86 ppm attributable to the two carbons that hold the two methoxy groups, and the characteristic peak of the carbon bearing the free OH at 139.08 ppm. Similarly, 3,4-dihydroxy-*N*-(1*H*-indol-5-yl)benzamide (**3h**) was characterized by the two common singlet peaks at 10.98 and 9.75 ppm representing the NH protons of the amide linkage and the indole ring. In addition, a broad singlet peak of 2H was found at 9.35 ppm, attributable to the two free OH phenolic moieties. The two carbons bearing these phenolic hydroxyl groups were detected in its ^13^C NMR spectrum at 148.82 and 145.20 ppm. The methyl group (CH_3_) of analogue **3i** was represented by a singlet peak (3H) in the aliphatic region at 2.34 ppm and a peak at 20.29 ppm (^13^C NMR).

In addition to the two common singlet peaks of the NH groups of the amide linker and the indole ring at 10.99 and 9.87 ppm, the third caffeic amide analogue **3j** also showed a broad peak of 2H representing the protons of the two hydroxyl groups at 9.28 ppm and a doublet peak at 6.58 with a coupling constant of 12.00 Hz attributable to a vinylic proton. The amide CO group was represented at 163.95 ppm (^13^C NMR), while the two carbons bearing the two hydroxyl groups were represented by two peaks at 147.88 and 146.01 ppm. A vinylic carbon of compound **3j** was also successfully detected at 140.11 ppm. In the ^13^C NMR spectrum of compound **3k**, the amide carbon and the carbon atom holding the free OH group were displayed above 160.00 ppm. In compound **3l**, the amide carbon appeared at 165.10 ppm, while the two carbons bearing the free OH and the methoxy groups were displayed at 150.00 and 147.58 ppm. The chemical shifts of the methoxy group in **3l** were represented by a singlet peak in the aliphatic region at 56.12 ppm (^13^C NMR) and 3.86 ppm (^1^H NMR). 

Finally, the chemical structure of the final indole-based caffeic acid amide analogue **3m** was confirmed by detecting three singlet peaks with chemical shifts of more than 8.00 ppm representing the three protons of the free OH, the amide NH, and the indole NH. Moreover, the two vinylic protons were clearly identified as two doublet peaks with *J* values of 16.00 Hz at 7.48 and 6.71 ppm. In addition, the six protons of the two methoxy groups were found as a long singlet peak at 3.83 ppm. The amide carbon appeared at 163.84 ppm, the two carbons bearing the two methoxy groups appeared as a long peak at 148.52 ppm, and the two aliphatic carbons of the methoxy groups showed a singlet peak at 56.37 ppm. These data proved and confirmed the formation and purity of the desired amide derivatives **3a**–**m**.

### 3.3. In Silico Druggability Studies of the Newly Synthesized Amide Derivatives **3a–m**

There is no guarantee that a small molecule that possesses a potent interaction with its target protein could be a successful therapeutic candidate. Poor absorption, distribution, metabolism, and excretion (ADME) characteristics may be the reason for this failure. Thus, many promising small molecules fail during the drug discovery process. Moreover, the drug development process is expensive. Accordingly, the pharmacokinetic (PK) characteristics of the indole-based benzamide and caffeic acid amide analogues (**3a**–**m**) were evaluated via the SwissADME platform by using distance/pharmacophore models coded as graph-based marks [54]. Using this platform, numerous crucial characteristics can be anticipated including the solubility of the final compounds, their gastrointestinal absorption, and brain entry abilities. During the different steps of the new drug development, these PK factors would constitute the foundation stone of the outcome’s anticipation [55]. 

**Figure 3:**
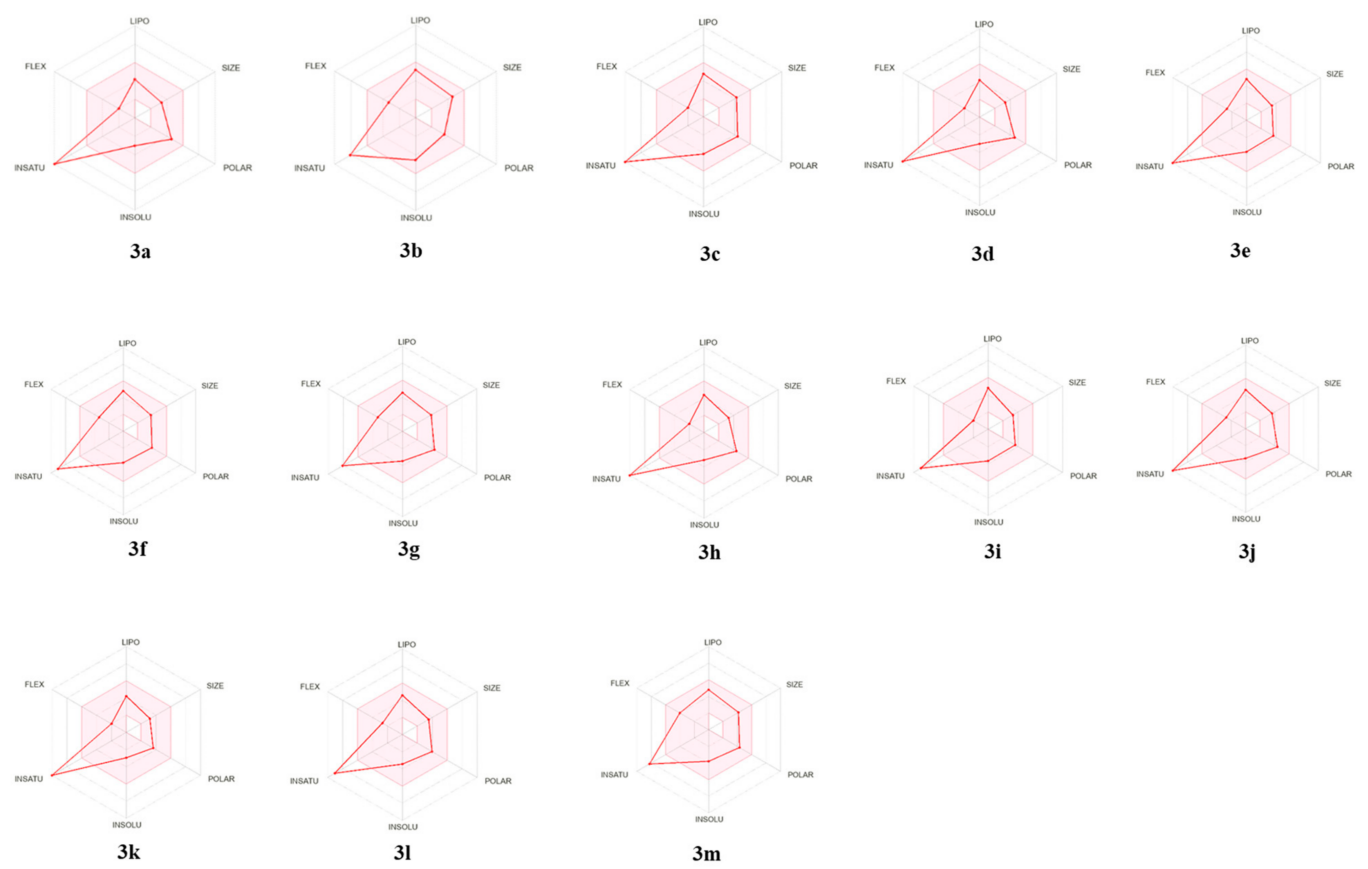
PK properties of the newly synthesized amides (**3a**–**m**) predicted by the SwissADME platform.

Another major PK property is the topological polar surface area (TPSA) of a compound which refers to the surface sum over the entire polar atoms, mainly nitrogen and oxygen, together with their associated hydrogen atoms. The TPSA is obtained by subtracting from the molecular surface the area of carbon atoms, halogens, and hydrogen atoms bonded to carbon atoms (i.e., nonpolar hydrogen atoms). TPSA is considered a great metric to improve the ability of a drug to penetrate the cells, which could enhance the efficacy of the synthesized drug candidate. Molecules possessing TPSA > 140 Å^2^ are predicted to not be able to cross cell membranes. On the other hand, a TPSA value < 90 Å^2^ was found to be essential for a drug candidate to cross the blood–brain barrier (BBB) [56]. Furthermore, compliance with the Lipinski rule of five [57] is another important guide on whether a compound can be taken orally. The outcomes of the in silico PK study are presented in Table 2 and Figure 3.

The predicted PK properties of all the newly synthesized indole-based benzamide and caffeic acid amide analogues (**3a**–**m**) revealed that all compounds would have high gastrointestinal absorption. In addition, all compounds displayed compliance to the Lipinski rule of five, indicating their great potential to be promising drug candidates with acceptable PK characteristics. Most compounds showed TPSA < 90 Å^2^, suggesting a potential antioxidant effect also in the brain to battle different neurodegenerative diseases, as hypothesized. These results suggest the PK stability of the indole-based benzamide and caffeic acid amide series.

### 3.4. In Vitro Antioxidant Assays (Free Radical Scavenging Effects)

#### 3.4.1. DPPH Radical Scavenging Activity

First, all the synthesized analogues (**3a**–**m**) were initially screened for their scavenging effects on the DPPH radical. Trolox was used as a reference (IC_50_ = 33.84 ± 1.01 µM). As illustrated in Table 3, our goal was to discover the antioxidant properties of incorporating the phenolic OH group(s) in addition to some other moieties such as methoxy, bromo, and amino groups in different positions on the phenyl ring, which is attached to position 5 of the antioxidant indole core via two different linkers (depending on the amide type, benzamide or caffeic acid amide). While compounds **3b**–**e**, **3g**, **3i**, **3k**, and **3l** showed moderate to weak activity as compared to Trolox, compounds **3a**, **3f**, **3h**, **3j**, and **3m** were found to have excellent radical scavenging activities with IC_50_ values of 95.81 ± 1.01, 136.8 ± 1.04, 86.77 ± 1.03, 50.98 ± 1.05, and 67.64 ± 1.02 µM. It was noted that compounds possessing the 3,4-dihydroxyphenyl moiety exhibited promising activities (the benzamide derivative **3h** and the caffeic acid amide derivative **3j**). On the other hand, the 4-amino-3-hydroxyphenyl moiety was only able to demonstrate its free radical scavenging effect in compound **3a,** which has the 4-amino-3-hydroxy phenyl moiety. The 4-hydroxy-3-methoxyphenyl and 4-hydroxy-3,5-dimethoxyphenyl moieties were only able to show their activities in the caffeic acid amide analogues **3f** and **3m**, respectively. It was also noticed that the majority of the synthesized caffeic acid amide derivatives (three out of four) were able to show higher free radical scavenging activity compared to their benzamide analogues. It could be the double-bond moiety in these caffeic acid amide derivatives that may increase the capacity of the molecule to interact with the free radicals via enhancing the electron conjugation effect in the whole chemical structure so that they do not engage in a destructive biochemical reaction. Based on this primary screening, the position and nature of substitutions on the phenyl moiety and the presence of the double bond in the middle of the structure were found to be essential factors directly affecting the free radical scavenging activity of these new indole-based amides. Consequently, the most potent derivatives (**3a**, **3f**, **3h, 3j**, and **3m**) were further evaluated.

#### 3.4.2. ABTS^•+^ Radical Cation Scavenging Assay

ABTS activity was measured in terms of percentage inhibition (%) of the ABTS^•+^ radical cation by each of the five most active compounds (**3a**, **3f**, **3h**, **3j**, and **3m**). The ABTS values of the five samples are presented in Table 3. While compound **3a** (with the 4-amino-3-hydroxy phenyl moiety) was able to scavenge the radical cation ABTS^•+^ with an IC_50_ value of 33.33 ± 1.96 µM, which is almost the similar potency of the standard Trolox (29.62 ± 1.86 µM), compound **3h** possessing 3,4-dihydroxy phenyl moiety showed a higher IC_50_ value of 39.98 ± 0.92 µM. Interestingly, three compounds out of the five (**3f**, **3j**, and **3m**) showed higher capacities to neutralize the radical cation ABTS^•+^ than Trolox with IC_50_ values of 14.48 ± 0.68, 19.49 ± 0.54, and 14.92 ± 0.30 µM, respectively.

#### 3.4.3. FRAP Assay

The three highly potent analogues (**3f**, **3j**, and **3m**) were considered for FRAP and ORAC assays. The FRAP assay assesses the antioxidant properties of the tested compound based on its reducing ability. The values obtained, shown in Table 3, were consistent with the DPPH and ABTS assays. In this study, compound **3j** (the caffeic acid derivative possessing 3,4-dihydroxyphenyl moiety) presented the highest antioxidant capacity with a FRAP value of 4774.37 ± 137.20 μM Trolox eq/mM sample, followed by compounds **3m** (4-hydroxy-3,5-dimethoxyphenyl moiety containing caffeic acid derivative) and **3f** (4-hydroxy-3-methoxyphenyl moiety containing caffeic acid derivative) with values of 2308.7 ± 73.73 and 1951.45 ± 75.97 μM Trolox eq/mM sample, respectively. Based on these findings, it could be concluded that caffeic amide analogue **3j** possessing the two phenolic OH groups not only offered the top free radical scavenging capability, but also the strongest reducing power among the tested compounds. Indeed, the antioxidant activity of a small molecule largely depends on both the chemical structure of the compound and the test system. Accordingly, it cannot be fully assessed by one single technique due to the various mechanisms of antioxidant action. As a result, the ORAC test was chosen to be the next further test for these three promising analogues (**3f**, **3j**, and **3m**). 

#### 3.4.4. ORAC Assay

Through the ORAC test, the antioxidant capacity was investigated of the three highly active compounds (**3f**, **3j**, and **3m**) that had demonstrated high antioxidant activity with the previous DPPH, ABTS, and FRAP tests. The ORAC test was intended to validate the results obtained with the previous approaches and extend the activity profile for each tested derivative. All tested compounds exhibited a dynamic ability to reduce the oxidative degradation of the fluorescent molecule, caused by peroxyl radicals. Compounds **3m** and **3f** showed very high ORAC antioxidant power (9253.47 ± 806.00 and 7293.46 ± 208.48 μM Trolox eq/mM sample, respectively). In a similar way to the previous assay (FRAP), the best antioxidant capacity against the peroxyl radicals was observed for compound **3j** (10,714.21 ± 817.76 μM Trolox eq/mM sample). 

## 4. Conclusions

As a step toward the development of novel free-radical scavenging hybrid agents for oxidative stress-related therapy, a new series of indole-based benzamide and caffeic acid amide analogues (**3a**–**m**) was successfully designed and synthesized. Among them, compounds **3a** (4-amino-3-hydroxy benzamide derivative), **3f** (4-hydroxy-3-methoxyphenyl containing caffeic acid derivative), **3h** (3,4-dihydroxy benzamide), **3j** (3,4-dihydroxyphenyl containing caffeic acid derivative), and **3m** (4-hydroxy-3,5-dimethoxyphenyl containing caffeic acid derivative) were able to show promising DPPH radical scavenging activities with IC_50_ values of 95.81 ± 1.01, 136.8 ± 1.04, 86.77 ± 1.03, 50.98 ± 1.05, and 67.64 ± 1.02 µM, respectively The three caffeic acid derivatives **3f**, **3j**, and **3m** neutralized the free radical cation ABTS^•+^ more than Trolox with IC_50_ values of 14.48 ± 0.68, 19.49 ± 0.54, and 14.92 ± 0.30 µM, respectively. Using FRAP and ORAC assays, compound **3j** was the most active antioxidant agent with values of 4774.37 ± 137.20 and 10,714.21 ± 817.76 μM Trolox eq/mM sample, respectively. Most small molecules were anticipated to be soluble and to penetrate the brain. No violations of the Lipinski rule of five were noticed, indicating a pharmacokinetically stable profile. Consequently, the hybrid compound **3j** is reported as a new antioxidant candidate with highly potent and promising free radical scavenging activities.

## Figures and Tables

**Table 1:** Yields and chemical structures of the synthesized analogues **3a**–**m**.

Comp.	Chemical Structure	Isolated Yield (%)
**3a**	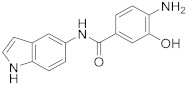	85
**3b**	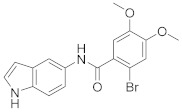	64
**3c**	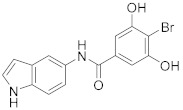	32
**3d**	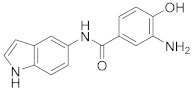	61
**3e**	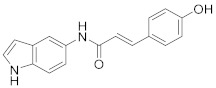	47
**3f**	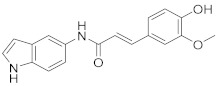	79
**3g**	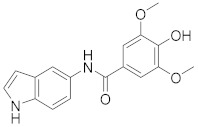	82
**3h**	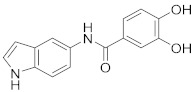	93
**3i**	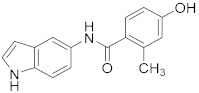	85
**3j**	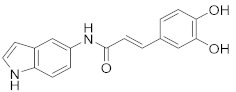	91
**3k**	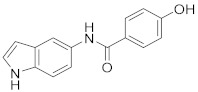	76
**3l**	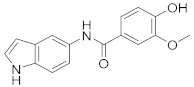	57
**3m**	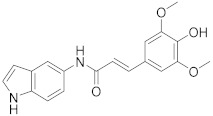	46

**Table 2:** Predicted lipophilicity, physicochemical, solubility (sol.), and drug-likeness properties of the newly synthesized indole-based benzamide and caffeic acid amide analogues **3a**–**m**.

Comp.	MW	TPSA	GTI Absorption	Lipinski #Violations	ESOL Log S	ESOL Class
**3a**	267.28	91.14	High	0	−3.02	Sol.
**3b**	375.22	63.35	High	0	−4.54	Moderately sol.
**3c**	347.16	85.35	High	0	−4.12	Moderately sol.
**3d**	267.28	91.14	High	0	−3.02	Sol.
**3e**	278.31	65.12	High	0	−3.69	Sol.
**3f**	308.33	74.35	High	0	−3.75	Sol.
**3g**	312.32	83.58	High	0	−3.49	Sol.
**3h**	268.27	85.35	High	0	−3.23	Sol.
**3i**	266.29	65.12	High	0	−3.67	Sol.
**3j**	294.30	85.35	High	0	−3.54	Sol.
**3k**	252.27	65.12	High	0	−2.96	Sol.
**3l**	282.29	74.35	High	0	−3.43	Sol.
**3m**	338.36	83.58	High	0	−3.81	Sol.

**Table 3:** Results of the in vitro radical scavenging activity assays (DPPH, ABTS, FRAP, and ORAC).

Comp.	DPPH Radical Scavenging Activity EC_50_ (Mean ± SD) µM	ABTS Radical Scavenging Activity IC_50_ (Mean ± SD) µM	FRAP Assay (μM Trolox eq/mM Sample)	ORAC Assay (μM Trolox eq/mM Sample)
**3a**	95.81 ± 1.01	33.33 ± 1.96	-	-
**3b**	12,527 ± 1.03	-	-	-
**3c**	15,422 ± 1.04	-	-	-
**3d**	14,451 ± 1.06	-	-	-
**3e**	2320 ± 1.01	-	-	-
**3f**	136.8 ± 1.04	14.48 ± 0.68	1951.45 ± 75.97	9253.47 ± 806.00
**3g**	5100 ± 1.03	-	-	-
**3h**	86.77 ± 1.03	39.98 ± 0.92	-	-
**3i**	>20,000	-	-	-
**3j**	50.98 ± 1.05	19.49 ± 0.54	4774.37 ± 137.20	10,714.21 ± 817.76
**3k**	>20,000	-	-	-
**3l**	8912 ± 1.02	-	-	-
**3m**	67.64 ± 1.02	14.92 ± 0.30	2308.7 ± 73.73	7293.46 ± 208.48
Trolox	33.84 ± 1.01	29.62 ± 1.86	-	-

## Supplementary Materials

The following are available online at https://www.mdpi.com/article/10.3390/metabo13020141/s1, chemical reagents, purification, and instrumentation details, ^1^HNMR, ^13^CNMR, purity, and HRMS data of the compounds reported in this study, in addition to the detailed calibration curves of Trolox used in RFAP and ORAC assays.

## References

[1] Kumar S., Gupta E., Kaushik S., Kumar Srivastava V., Mehta S.K., Jyoti A. (2018). Evaluation of oxidative stress and antioxidant status: Correlation with the severity of sepsis. Scand. J. Immunol..

[2] Darenskaya M.A., Kolesnikova L.I., Kolesnikov S.I. (2021). Oxidative Stress: Pathogenetic Role in Diabetes Mellitus and Its Complications and Therapeutic Approaches to Correction. Bull. Exp. Biol. Med..

[3] Pohanka M. (2013). Role of oxidative stress in infectious diseases. A review. Folia Microbiol..

[4] Sies H. (2015). Oxidative stress: A concept in redox biology and medicine. Redox Biol..

[5] Poprac P., Jomova K., Simunkova M., Kollar V., Rhodes C.J., Valko M. (2017). Targeting Free Radicals in Oxidative Stress-Related Human Diseases. Trends Pharmacol. Sci..

[6] Liu R., Mabury S.A. (2020). Synthetic Phenolic Antioxidants: A Review of Environmental Occurrence, Fate, Human Exposure, and Toxicity. Environ. Sci. Technol..

[7] Wang W., Xiong P., Zhang H., Zhu Q., Liao C., Jiang G. (2021). Analysis, occurrence, toxicity and environmental health risks of synthetic phenolic antioxidants: A review. Environ. Res..

[8] Neha K., Haider M.R., Pathak A., Yar M.S. (2019). Medicinal prospects of antioxidants: A review. Eur. J. Med. Chem..

[9] Kundu T., Pramanik A. (2020). Expeditious and eco-friendly synthesis of new multifunctionalized pyrrole derivatives and evaluation of their antioxidant property. Bioorganic Chem..

[10] Kundu T., Bhattacharjee B., Hazra S., Ghosh A.K., Bandyopadhyay D., Pramanik A. (2019). Synthesis and Biological Assessment of Pyrrolobenzoxazine Scaffold as a Potent Antioxidant. J. Med. Chem..

[11] Bandeira P.T., Dalmolin M.C., de Oliveira M.M., Nunes K.C., Garcia F.P., Nakamura C.V., de Oliveira A.R.M., Piovan L. (2019). Synthesis, Antioxidant Activity and Cytotoxicity of N-Functionalized Organotellurides. Bioorganic Med. Chem..

[12] Tailor N., Sharma M. (2013). Antioxidant hybrid compounds: A promising therapeutic intervention in oxidative stress induced diseases. Mini Rev. Med. Chem..

[13] Vavříková E., Křen V., Jezova-Kalachova L., Biler M., Chantemargue B., Pyszková M., Riva S., Kuzma M., Valentová K., Ulrichová J. (2017). Novel flavonolignan hybrid antioxidants: From enzymatic preparation to molecular rationalization. Eur. J. Med. Chem..

[14] Carullo G., Mazzotta S., Koch A., Hartmann K.M., Friedrich O., Gilbert D.F., Vega-Holm M., Schneider-Stock R., Aiello F. (2020). New Oleoyl Hybrids of Natural Antioxidants: Synthesis and In Vitro Evaluation as Inducers of Apoptosis in Colorectal Cancer Cells. Antioxidants.

[15] Gülçin I. (2006). Antioxidant activity of caffeic acid (3,4-dihydroxycinnamic acid). Toxicology.

[16] Sofic E., Rimpapa Z., Kundurovic Z., Sapcanin A., Tahirovic I., Rustembegovic A., Cao G.J. (2005). Antioxidant capacity of the neurohormone melatonin. J. Neural Transm..

[17] Cipolla-Neto J., Amaral F.G.D. (2018). Melatonin as a Hormone: New Physiological and Clinical Insights. Endocr. Rev..

[18] Reiter R.J., Mayo J.C., Tan D.X., Sainz R.M., Alatorre-Jimenez M., Qin L. (2016). Melatonin as an antioxidant: Under promises but over delivers. J. Pineal Res..

[19] Reiter R.J., Tan D.X., Cabrera J., D’Arpa D. (1999). Melatonin and tryptophan derivatives as free radical scavengers and antioxidants. Adv. Exp. Med. Biol..

[20] Reiter R.J., Tan D.X., Rosales-Corral S., Galano A., Zhou X.J., Xu B. (2018). Mitochondria: Central Organelles for Melatonin’s Antioxidant and Anti-Aging Actions. Molecules.

[21] Reiter R.J., Rosales-Corral S., Tan D.X., Jou M.J., Galano A., Xu B. (2017). Melatonin as a mitochondria-targeted antioxidant: One of evolution’s best ideas. Cell. Mol. Life Sci. CMLS.

[22] Velkov Z.A., Velkov Y.Z., Galunska B.T., Paskalev D.N., Tadjer A.V. (2009). Melatonin: Quantum-chemical and biochemical investigation of antioxidant activity. Eur. J. Med. Chem..

[23] Estevão M.S., Carvalho L.C., Ribeiro D., Couto D., Freitas M., Gomes A., Ferreira L.M., Fernandes E., Marques M.M.B. (2010). Antioxidant activity of unexplored indole derivatives: Synthesis and screening. Eur. J. Med. Chem..

[24] Esposito E., Cuzzocrea S. (2010). Antiinflammatory activity of melatonin in central nervous system. Curr. Neuropharmacol..

[25] Di Bella G., Mascia F., Gualano L., Di Bella L. (2013). Melatonin anticancer effects. Int. J. Mol. Sci..

[26] de Zanette S.A., Vercelino R., Laste G., Rozisky J.R., Schwertner A., Machado C.B., Xavier F., de Souza I.C.C., Deitos A., Torres I.L. (2014). Melatonin analgesia is associated with improvement of the descending endogenous pain-modulating system in fibromyalgia: A phase II, randomized, double-dummy, controlled trial. BMC Pharmacol. Toxicol..

[27] Lochner A., Marais E., Huisamen B. (2018). Melatonin and cardioprotection against ischaemia/reperfusion injury: What’s new? A review. J. Pineal Res..

[28] Chen J., Xia H., Zhang L., Zhang H., Wang D., Tao X. (2019). Protective effects of melatonin on sepsis-induced liver injury and dysregulation of gluconeogenesis in rats through activating SIRT1/STAT3 pathway. Biomed. Pharmacother..

[29] Rahman A., Hasan A.U., Kobori H. (2019). Melatonin in chronic kidney disease: A promising chronotherapy targeting the intrarenal renin–angiotensin system. Hypertens. Res..

[30] Tordjman S., Chokron S., Delorme R., Charrier A., Bellissant E., Jaafari N., Fougerou C. (2017). Melatonin: Pharmacology, Functions and Therapeutic Benefits. Curr. Neuropharmacol..

[31] Chitimus D.M., Popescu M.R., Voiculescu S.E., Panaitescu A.M., Pavel B., Zagrean L., Zagrean A.M. (2020). Melatonin’s Impact on Antioxidative and Anti-Inflammatory Reprogramming in Homeostasis and Disease. Biomolecules.

[32] Salehi B., Sharopov F., Fokou P.V.T., Kobylinska A., Jonge L., Tadio K., Sharifi-Rad J., Posmyk M.M., Martorell M., Martins N. (2019). Melatonin in Medicinal and Food Plants: Occurrence, Bioavailability, and Health Potential for Humans. Cells.

[33] Rodríguez-Franco M.I., Fernández-Bachiller M.I., Pérez C., Hernández-Ledesma B., Bartolomé B. (2006). Novel tacrine—Melatonin hybrids as dual-acting drugs for Alzheimer disease, with improved acetylcholinesterase inhibitory and antioxidant properties. J. Med. Chem..

[34] Luo X.-T., Wang C.-M., Liu Y., Huang Z.-G. (2015). New multifunctional melatonin-derived benzylpyridinium bromides with potent cholinergic, antioxidant, and neuroprotective properties as innovative drugs for Alzheimer’s disease. Eur. J. Med. Chem..

[35] Bautista-Aguilera O.M., Esteban G., Bolea I., Nikolic K., Agbaba D., Moraleda I., Iriepa I., Samadi A., Soriano E., Unzeta M. (2014). Design, synthesis, pharmacological evaluation, QSAR analysis, molecular modeling and ADMET of novel donepezil-indolyl hybrids as multipotent cholinesterase/monoamine oxidase inhibitors for the potential treatment of Alzheimer’s disease. Eur. J. Med. Chem..

[36] Rivara S., Pala D., Bedini A., Spadoni G. (2015). Therapeutic uses of melatonin and melatonin derivatives: A patent review (2012*–*2014). Expert Opin. Ther. Pat..

[37] Wang S.Y., Shi X.C., Laborda P. (2020). Indole-based melatonin analogues: Synthetic approaches and biological activity. Eur. J. Med. Chem..

[38] Mor M., Rivara S., Silva C., Bordi F., Plazzi P.V., Spadoni G., Diamantini G., Balsamini C., Tarzia G., Fraschini F. (1998). Melatonin receptor ligands: Synthesis of new melatonin derivatives and comprehensive comparative molecular field analysis (CoMFA) study. J. Med. Chem..

[39] Landagaray E., Ettaoussi M., Leclerc V., Traoré B., Perez V., Nosjean O., Boutin J.A., Caignard D.H., Delagrange P., Berthelot P. (2014). New melatonin (MT1/MT2) ligands: Design and synthesis of (8,9-dihydro-7H-furo[3,2-f]chromen-1-yl) derivatives. Bioorganic Med. Chem..

[40] Angelova V.T., Rangelov M., Todorova N., Dangalov M., Andreeva-Gateva P., Kondeva-Burdina M., Karabeliov V., Shivachev B., Tchekalarova J. (2019). Discovery of novel indole-based aroylhydrazones as anticonvulsants: Pharmacophore-based design. Bioorganic Chem..

[41] Tan D.X., Reiter R.J., Manchester L.C., Yan M.T., El-Sawi M., Sainz R.M., Mayo J.C., Kohen R., Allegra M., Hardeland R. (2002). Chemical and physical properties and potential mechanisms: Melatonin as a broad spectrum antioxidant and free radical scavenger. Curr. Top. Med. Chem..

[42] Stasica P., Ulanski P., Rosiak J.M. (1998). Melatonin as a hydroxyl radical scavenger. J. Pineal Res..

[43] Reiter R.J. (1998). Cytoprotective properties of melatonin: Presumed association with oxidative damage and aging. Nutrition.

[44] Elkamhawy A., Kim H.J., Elsherbeny M.H., Paik S., Park J.H., Gotina L., Abdellattif M.H., Gouda N.A., Cho J., Lee K. (2021). Discovery of 3,4-dichloro-N-(1H-indol-5-yl)benzamide: A highly potent, selective, and competitive hMAO-B inhibitor with high BBB permeability profile and neuroprotective action. Bioorganic Chem..

[45] Elsherbeny M.H., Kim J., Gouda N.A., Gotina L., Cho J., Pae A.N., Lee K., Park K.D., Elkamhawy A., Roh E.J. (2021). Highly Potent, Selective, and Competitive Indole-Based MAO-B Inhibitors Protect PC12 Cells against 6-Hydroxydopamine- and Rotenone-Induced Oxidative Stress. Antioxidants.

[46] Elkamhawy A., Paik S., Kim H.J., Park J.H., Londhe A.M., Lee K., Pae A.N., Park K.D., Roh E.J. (2020). Discovery of N-(1-(3-fluorobenzoyl)-1H-indol-5-yl)pyrazine-2-carboxamide: A novel, selective, and competitive indole-based lead inhibitor for human monoamine oxidase B. J. Enzym. Inhib. Med. Chem..

[47] Yeon S.K., Choi J.W., Park J.-H., Lee Y.R., Kim H.J., Shin S.J., Jang B.K., Kim S., Bahn Y.-S., Han G. (2018). Synthesis and evaluation of biaryl derivatives for structural characterization of selective monoamine oxidase B inhibitors toward Parkinson’s disease therapy. Bioorganic Med. Chem..

[48] Cores Á., Abril S., Michalska P., Duarte P., Olives A.I., Martín M.A., Villacampa M., León R., Menéndez J.C. (2021). Bisavenathramide Analogues as Nrf2 Inductors and Neuroprotectors in In Vitro Models of Oxidative Stress and Hyperphosphorylation. Antioxidants.

[49] Elkamhawy A., Park J.E., Hassan A.H.E., Pae A.N., Lee J., Park B.G., Roh E.J. (2018). Synthesis and evaluation of 2-(3-arylureido)pyridines and 2-(3-arylureido)pyrazines as potential modulators of Aβ-induced mitochondrial dysfunction in Alzheimer’s disease. Eur. J. Med. Chem..

[50] Boly R., Lamkami T., Lompo M., Dubois J., Guissou I. (2016). DPPH free radical scavenging activity of two extracts from Agelanthus dodoneifolius (Loranthaceae) leaves. Int. J. Toxicol. Pharmacol. Res..

[51] Arnao M.B., Cano A., Acosta M. (2001). The hydrophilic and lipophilic contribution to total antioxidant activity. Food Chem..

[52] Benzie I.F.F., Strain J.J. (1996). The Ferric Reducing Ability of Plasma (FRAP) as a Measure of “Antioxidant Power”: The FRAP Assay. Anal. Biochem..

[53] Liang Z., Cheng L., Zhong G.Y., Liu R.H. (2014). Antioxidant and antiproliferative activities of twenty-four Vitis vinifera grapes. PLoS ONE.

[54] Daina A., Michielin O., Zoete V. (2017). SwissADME: A free web tool to evaluate pharmacokinetics, drug-likeness and medicinal chemistry friendliness of small molecules. Sci. Rep..

[55] Alavijeh M.S., Chishty M., Qaiser M.Z., Palmer A.M. (2005). Drug metabolism and pharmacokinetics, the blood-brain barrier, and central nervous system drug discovery. NeuroRx J. Am. Soc. Exp. NeuroTherapeutics.

[56] Pajouhesh H., Lenz G.R. (2005). Medicinal chemical properties of successful central nervous system drugs. NeuroRx J. Am. Soc. Exp. NeuroTherapeutics.

[57] Lipinski C.A. (2004). Lead- and drug-like compounds: The rule-of-five revolution. Drug Discov. Today: Technol..

