# Supplementary material for: Novel Hybrid Indole-Based Caffeic Acid Amide Derivatives as Potent Free Radical Scavenging Agents: Rational Design, Synthesis, Spectroscopic Characterization, In Silico and In Vitro Investigations
Source: Metabolites. 2023 Jan 17;13(2):141. doi: 10.3390/metabo13020141 (PMC9966950; doi:10.3390/metabo13020141)
Supplement: Supplementary file 1 [file metabolites-13-00141-s001.zip › metabolites-2124504-supplementary.pdf]

Supplementary Material

Chemical Reagents, Purification, and Instrumentation

In a summary, all *reagents* and *solvents* were utilized *with no additional* purification. ^{1}H NMR *data* were obtained using Varian 400 MHz spectrometer (Varian Medical Systems, Inc., Palo Alto, CA, USA), with chemical shifts being determined in parts per million (ppm) and coupling constants in Hz. The high-resolution electrospray ionization mass spectrometry (HR-ESIMS) data were evaluated utilizing a JMS–700 mass spectrometer (Jeol, Japan) or by HR-ESIMS data obtained via a G2 QTOF mass spectrometer. Reaction monitoring was performed using TLC on 0.25 mm silica plates (E. Merck; silica gel 60 F_{254}). To verify the purity of the final target hybrids a reverse-phase high-performance liquid chromatography (RP-HPLC) was used and equipped with an ultraviolet (UV) detector set at 254 nm. The employed mobile phases were: (A) H_2O containing 0.05% trifluoroacetic acid; and (B) CH_3CN. HPLC employed a YMC Hydrosphere C_{18} (HS-302) column (5-µm particle size, 12-nm pore size) that was 4.6 mm in diameter × 150 mm in size with a flow rate of 1.0 mL/min. The melting points (M.p.) were assessed with a Fisherbrand digital M.p. apparatus.

DPPH Assay

In brief, 100µL of freshly prepared DPPH reagent (0.1% in methanol) was added to 100 µL of the sample in 96 wells plate (n=6), and the reaction was incubated at 25 °C for 30 min in dark. At the end of incubation time the resulting reduction in DPPH color intensity was calculated at 540 nm. Data are displayed as means ± SD according to the following equation: percentage inhibition=((Average absorbance of blank-average absorbance of the test)/(Average absorbance of blank))*100. The assay was carried out at Nawah Scientific Center for Research, Cairo, Egypt. The results were noted using a microplate reader FluoStar Omega. Data was analyzed using Microsoft Excel® and the EC_{50} value was processed using Graph pad Prism 6® by converting the concentrations to their logarithmic value and selecting the non-linear inhibitor regression equation (log (inhibitor) vs. normalized response – variable slope equation).

ABTS assay

In brief, 192 mg of ABTS were dissolved in distilled water and moved to a 50 mL volumetric flask then the volume was completed with distilled water. Then, 1 mL of the previous solution was added to 17 µL of 140 mM potassium persulphate and the mixture was left in the dark for 24 hours. A volume of 1 mL of the reaction mixture was completed to 50 mL with methanol to obtain the final ABTS dilution. The freshly prepared ABTS reagent (190 µL) was mixed with 10 µL of the sample/compound in 96 wells plate (n=6), and the reaction was incubated at 25 °C for 30 min in dark. After incubation, the decrease in ABTS color intensity was assessed at 734 nm. Data are displayed as means ± SD according to the following equation: percentage inhibition=((Average

absorbance of blank-average absorbance of the test)/(Average absorbance of blank))*100. The assay was performed at Nawah Scientific Center for Research, Cairo, Egypt. The results were recorded using microplate reader FluoStar Omega. Data were investigated using Microsoft Excel® and the IC_{50} value was calculated using Graph pad Prism 6® by changing the concentrations to their logarithmic value and choosing a non-linear inhibitor regression equation (log (inhibitor) vs. normalized response – variable slope equation).

FRAB Assay

A freshly prepared TPTZ reagent (300 mM acetate buffer (PH = 3.6), 10 mM TPTZ in 40 mM HCl, and 20 mM $FeCl_3$, in a ratio of 10:1:1 v/v/v, respectively). The freshly prepared TPTZ reagent (190 µL) was combined with 10 µL of the sample in 96 wells plate (n=3), and the reaction was incubated at 25 °C for 30 min in dark. After incubation, the resulting blue color was assessed at 593 nm. The assay was performed at Nawah Scientific Center for Research, Cairo, Egypt. Data are displayed as means ± SD. The results were noted using a microplate reader FluoStar Omega. The ferric reducing ability of the samples is described as µM TE/mg sample using the linear regression equation extracted from the calibration curve (linear dose-response curve of Trolox, Figure S1).

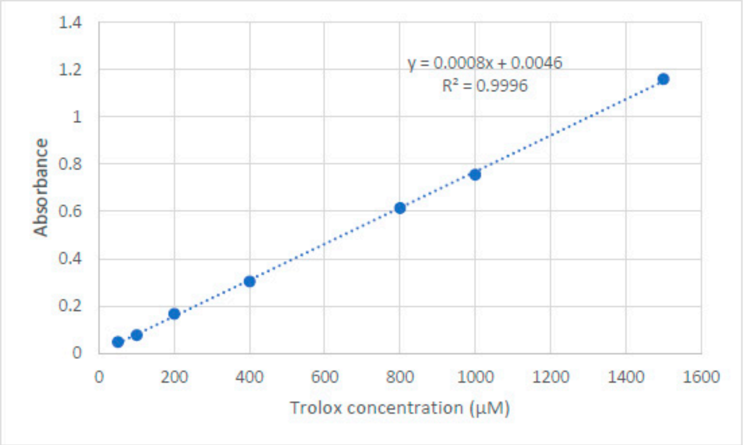

Concentration (µM)	Absorbance
50	0.048
100	0.077
200	0.167
400	0.304
800	0.615
1000	0.756
1500	1.161

Dose response line for solutions of Trolox.

*The average of the readings of the 3 replicates was taken after subtraction from the Blank (FRAP reagent+ solvent)

Figure S1: The calibration curve (linear dose-response curve of Trolox) used in FRAP assay

ORAC assay

In brief, 10 µL of the prepared sample(s) were incubated with 30 µL fluoresceine (100 nM) for 10 min at 37 °C. Fluorescence measurement (485 EX, 520 EM, nm) was performed for three cycles (cycle time, 90 sec.) for background measurement. Afterward, 70 µL of freshly prepared 2,2'-Azobis(2-amidinopropane) dihydrochloride (AAPH) (300 mM) were added to each well. Fluorescence measurement (485 EX, 520 EM, nm) was continued for 60 min (40 cycles, each 90 sec.). Data are displayed as means (n = 3) ± SD and the antioxidant effect of the compound/extract was determined as µM Trolox equivalents by substitution in the linear regression equation: y = 3000.2x - 67625. The assay was performed at Nawah Scientific Center for Research, Cairo, Egypt. The results were recorded using a microplate reader FluoStar Omega. Results of the samples were given as µM TE/ mg sample using the linear regression equation extracted from the calibration curve (linear dose-inhibition curve of Trolox, Figure S2).

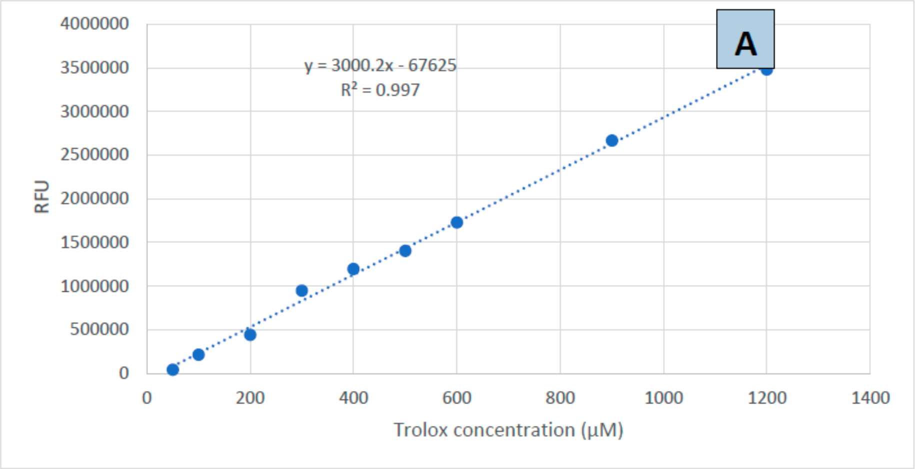

Figure S2. The calibration curve (linear dose-inhibition curve of Trolox) used in ORAC assay

Compound **3a** - (^{1}H NMR, ^{13} C NMR, HPLC, and HRMS data charts)

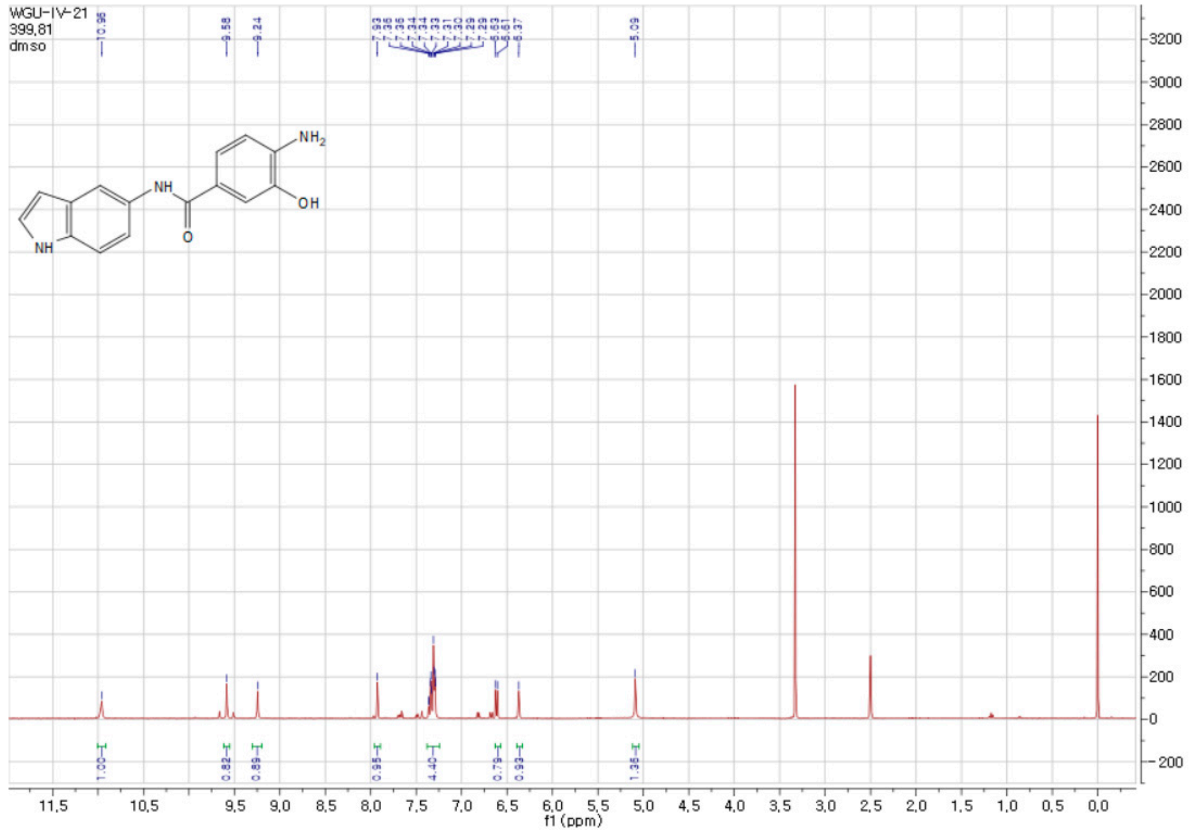

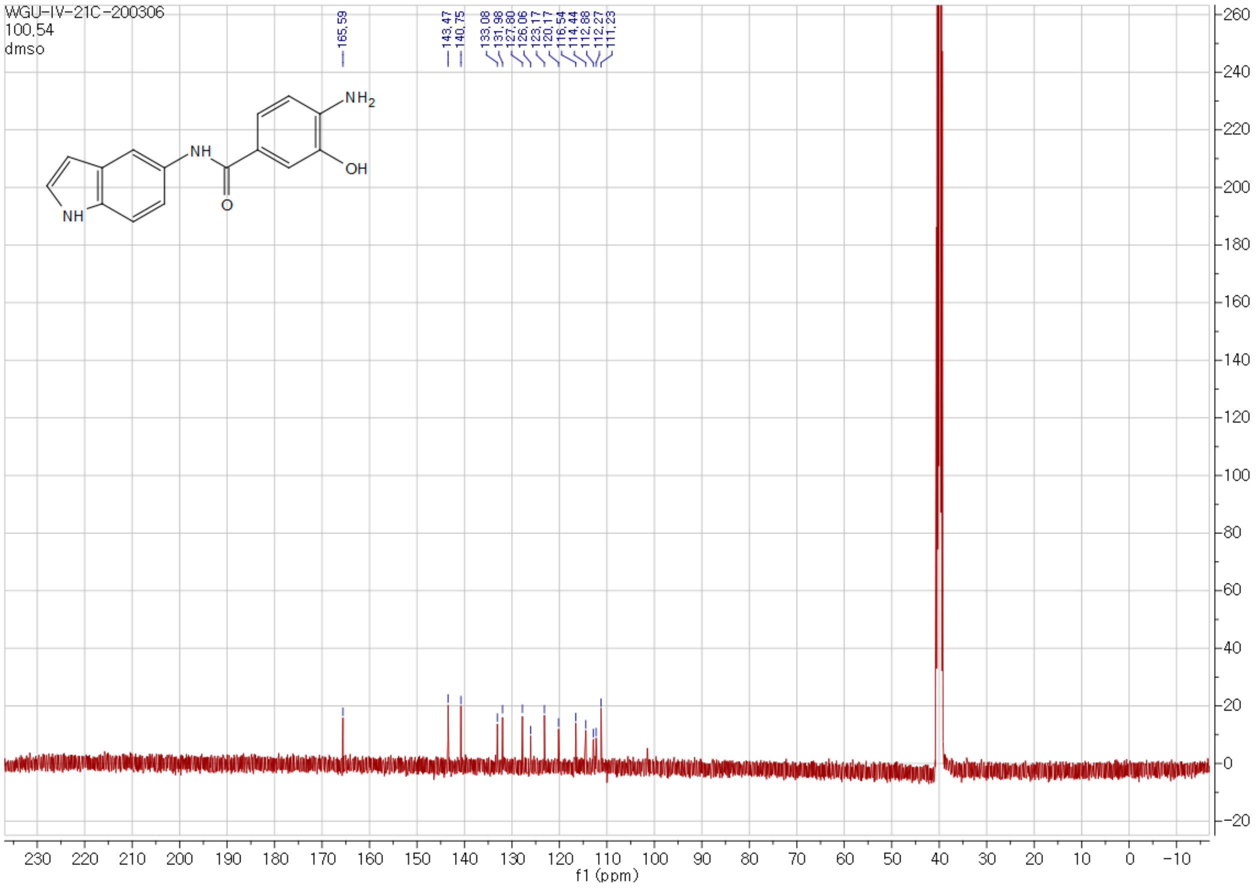
WGU-IV-21C-200306
100.54
dmso
165.59
143.47
140.75
133.08
131.98
127.80
126.06
123.17
120.17
116.54
114.44
112.88
112.27
111.23
NH
NH$_2$
OH
O
NH
f1 (ppm)

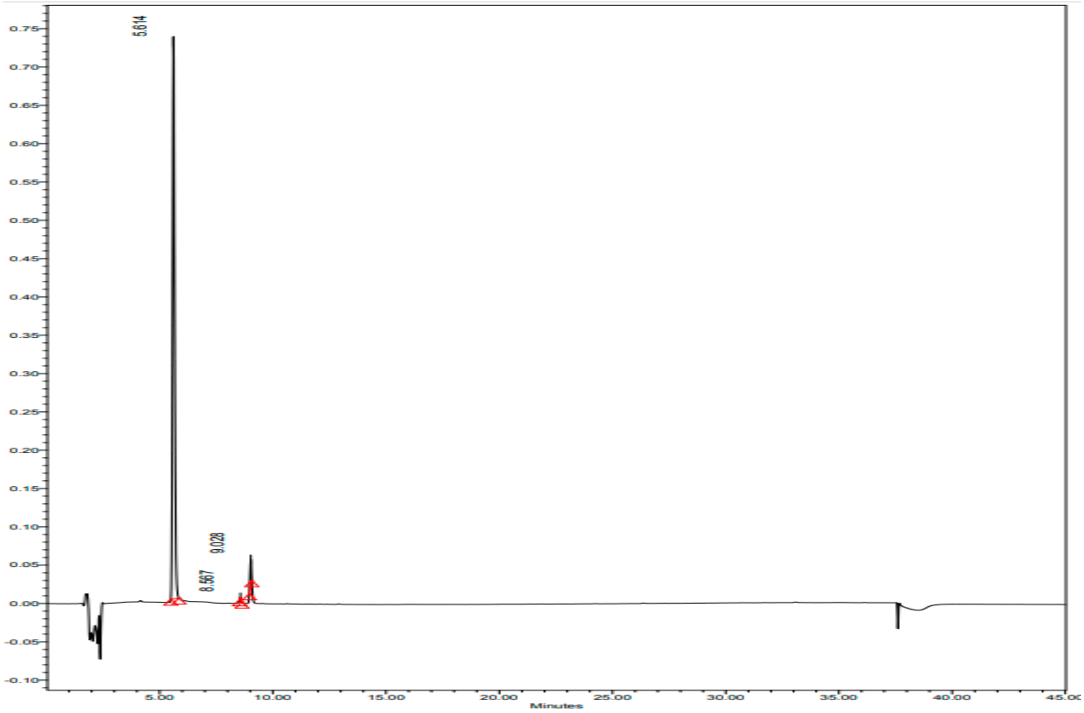
5.614
8.567
9.028
Minutes

	Name	Retention Time (min)	Purity1 Angle	Purity1 Threshold	PDA Match1 Spect. Name	PDA Match1 Angle	PDA Match1 Threshold	PDA Match1 Lib. Name	Area (µV*sec)	% Area	Height (µV)	Int Type	Amount	Units
1		5.614							4697962	96.71	732306	bb		
2		8.567							16715	0.34	4069	bb		
3		9.028							142913	2.94	34542	bb		

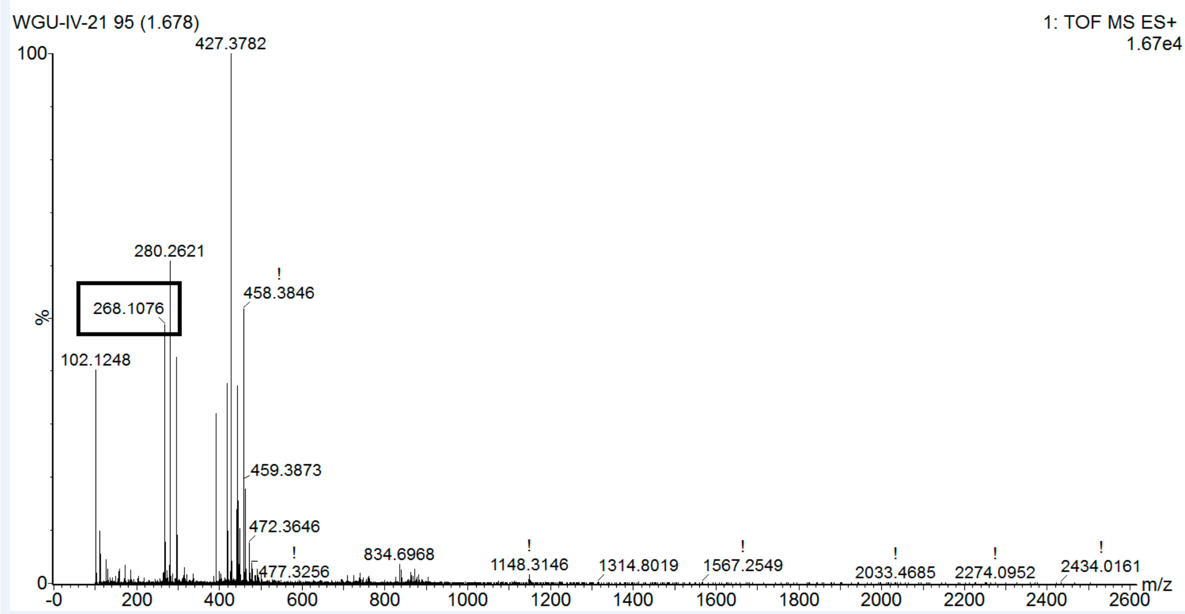

Compound **3b** - (^{1}H NMR, ^{13} C NMR, HPLC, and HRMS original data charts)

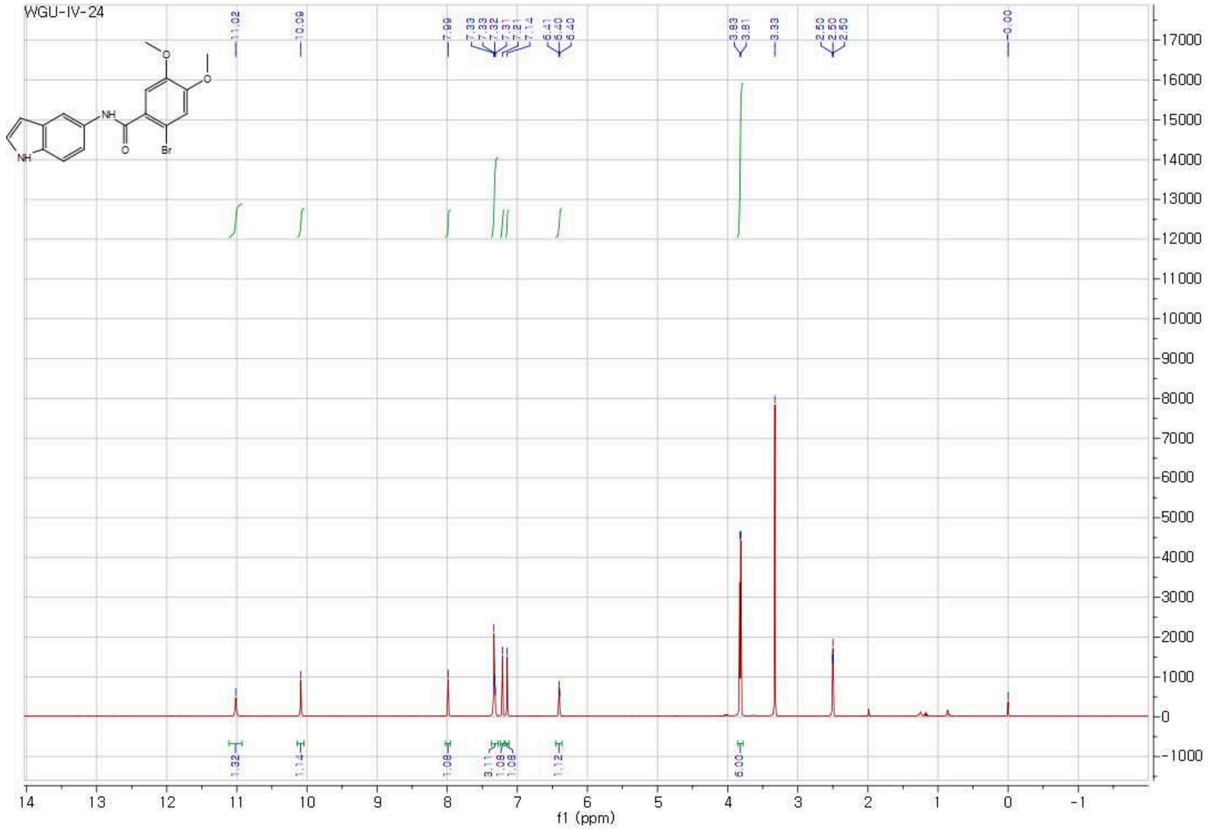

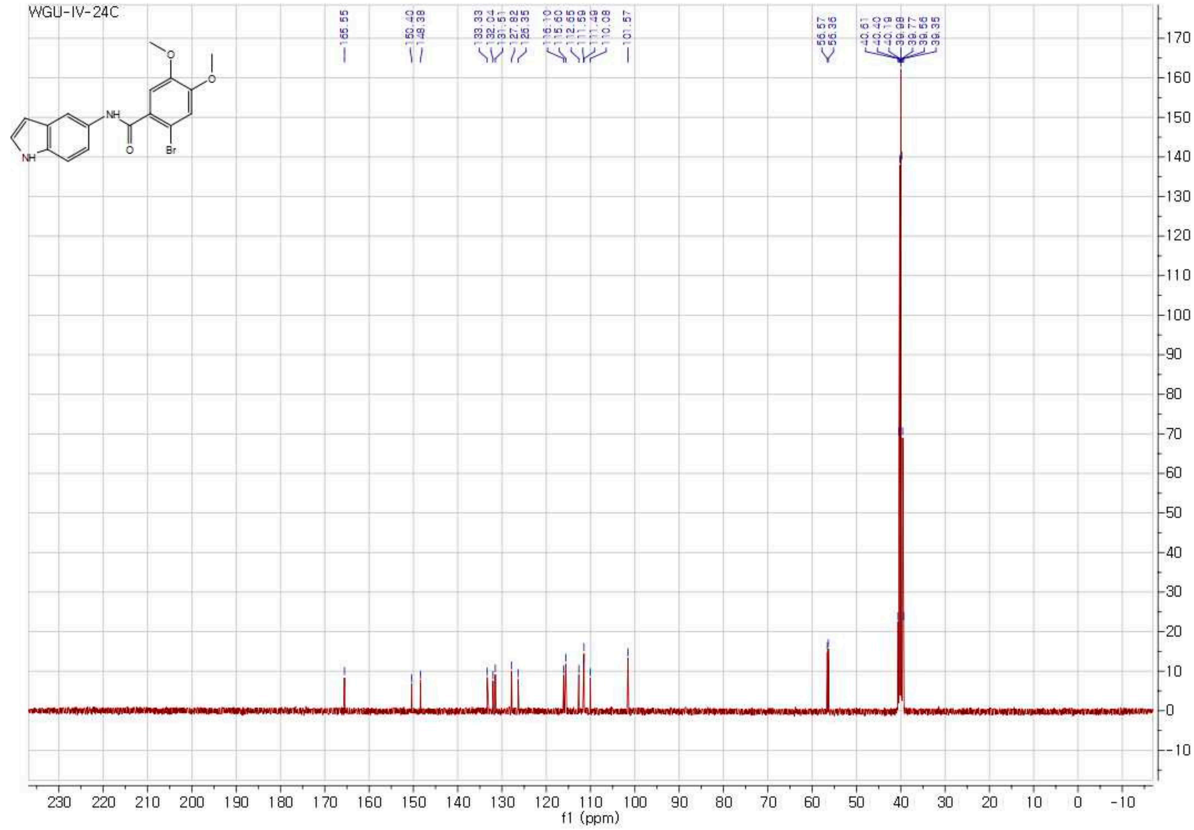
WGU-IV-24C
f1 (ppm)

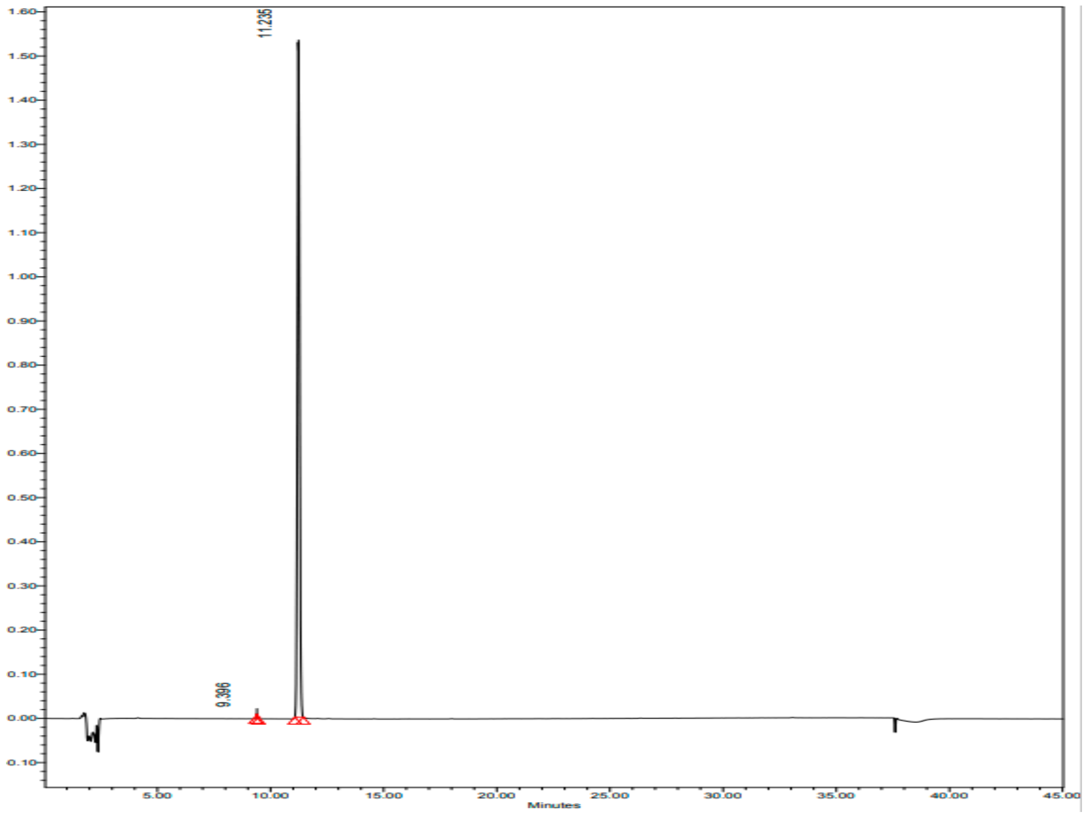
11.235
9.396
Minutes

	Name	Retention Time (min)	Purity1 Angle	Purity1 Threshold	PDA Match1 Spect. Name	PDA Match1 Angle	PDA Match1 Threshold	PDA Match1 Lib. Name	Area (µV*sec)	% Area	Height (µV)	Int Type	Amount
1		9.396							27679	0.27	6663	bb	
2		11.235							10377858	99.73	1536413	bb	

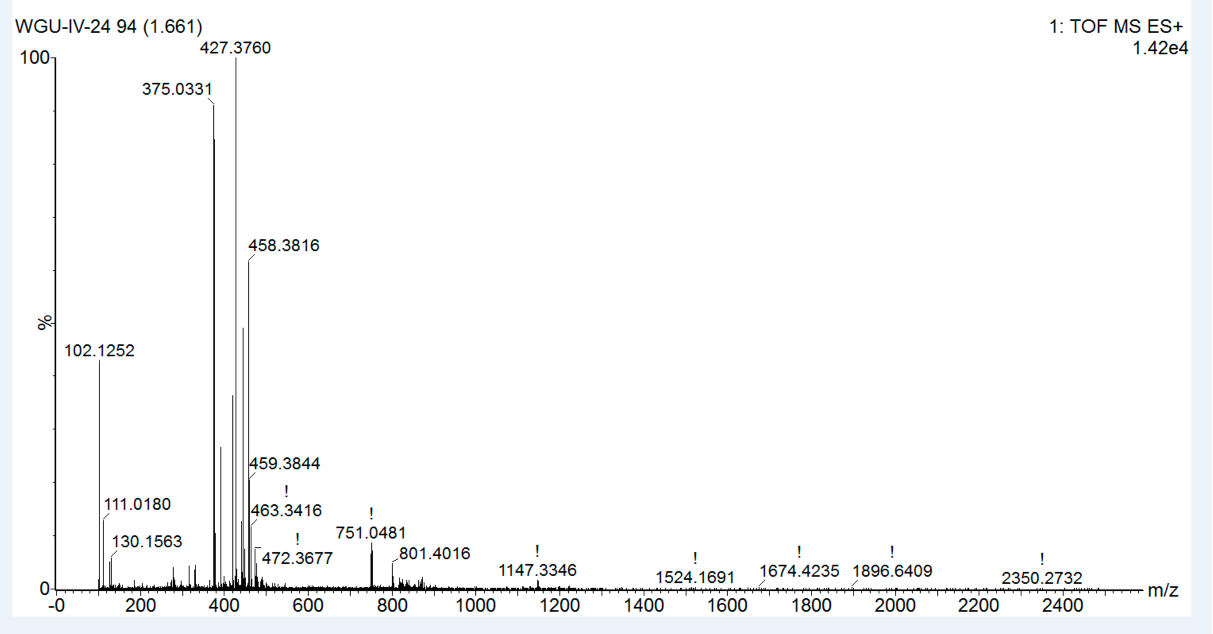

Compound **3c** - (^{1}H NMR, [13] C NMR, HPLC, and HRMS data charts)

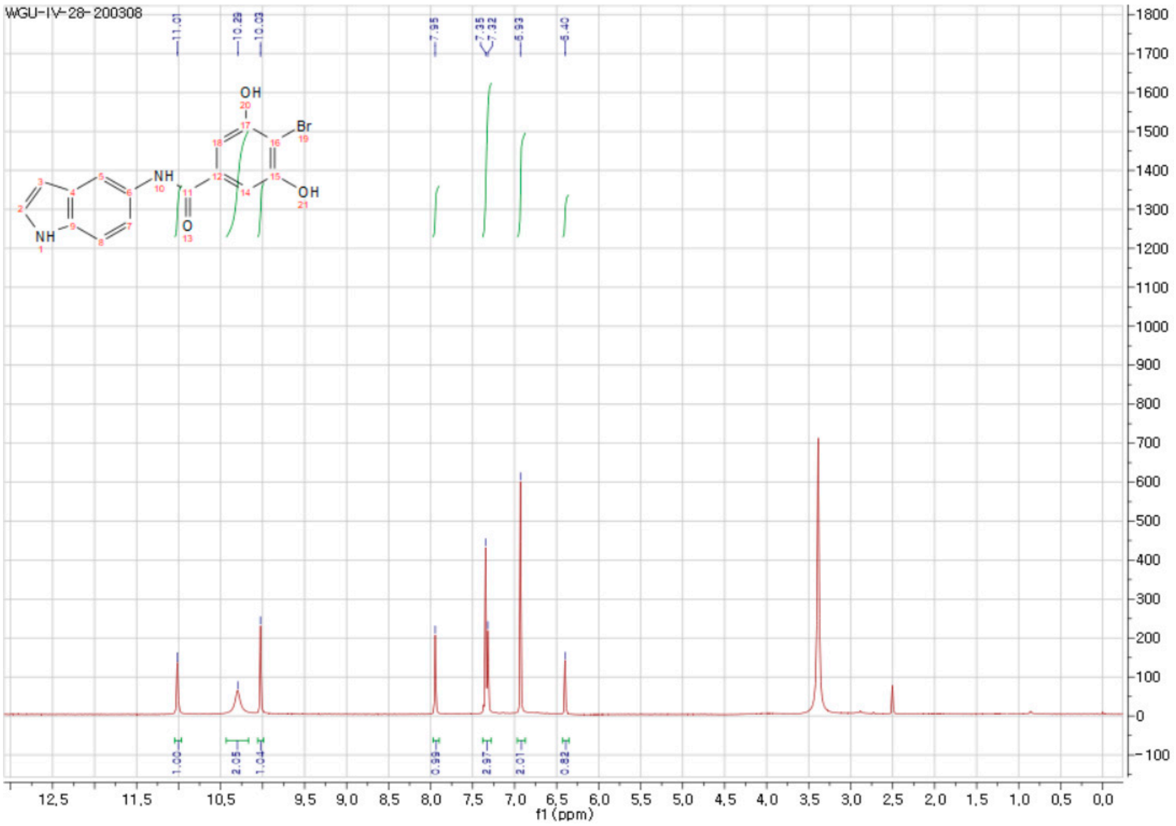

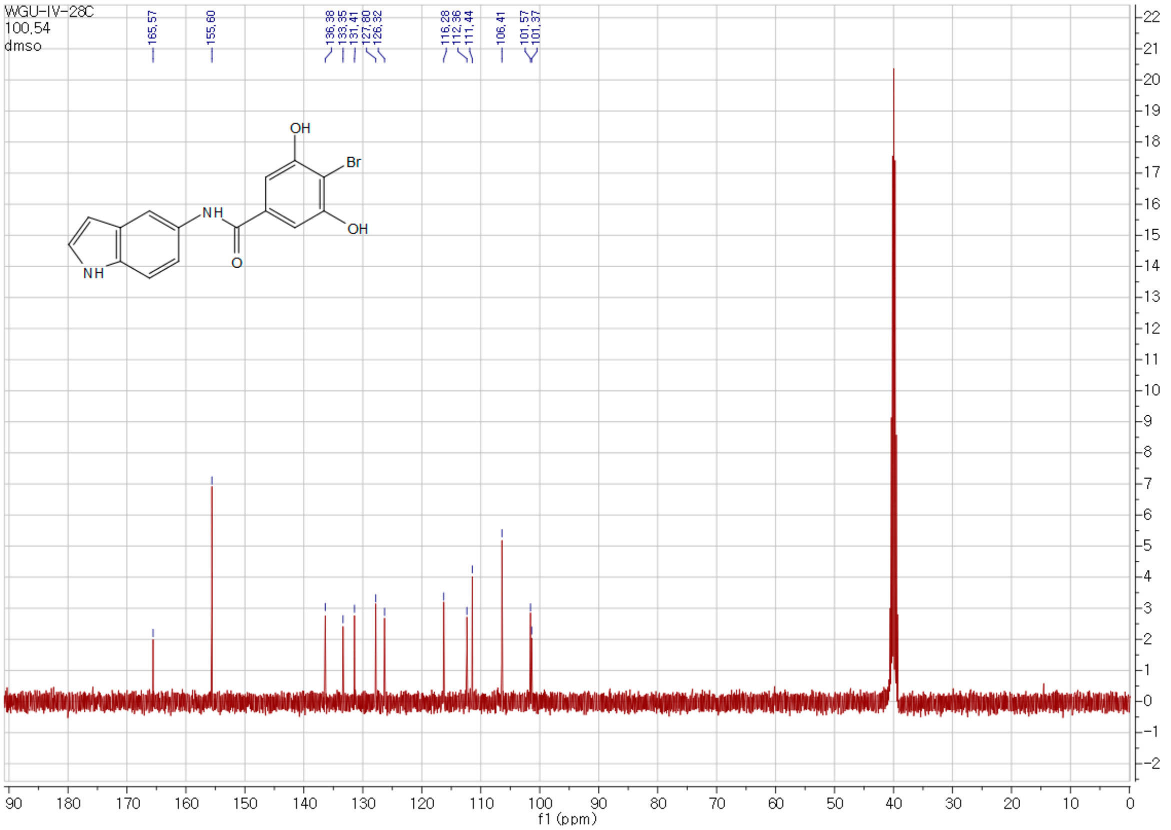
WGU-IV-28C
100.54
dmso
165.57
155.60
136.38
133.35
131.41
127.80
126.32
116.28
112.36
111.44
106.41
101.57
101.37
OH
Br
NH
OH
O
NH
f1 (ppm)

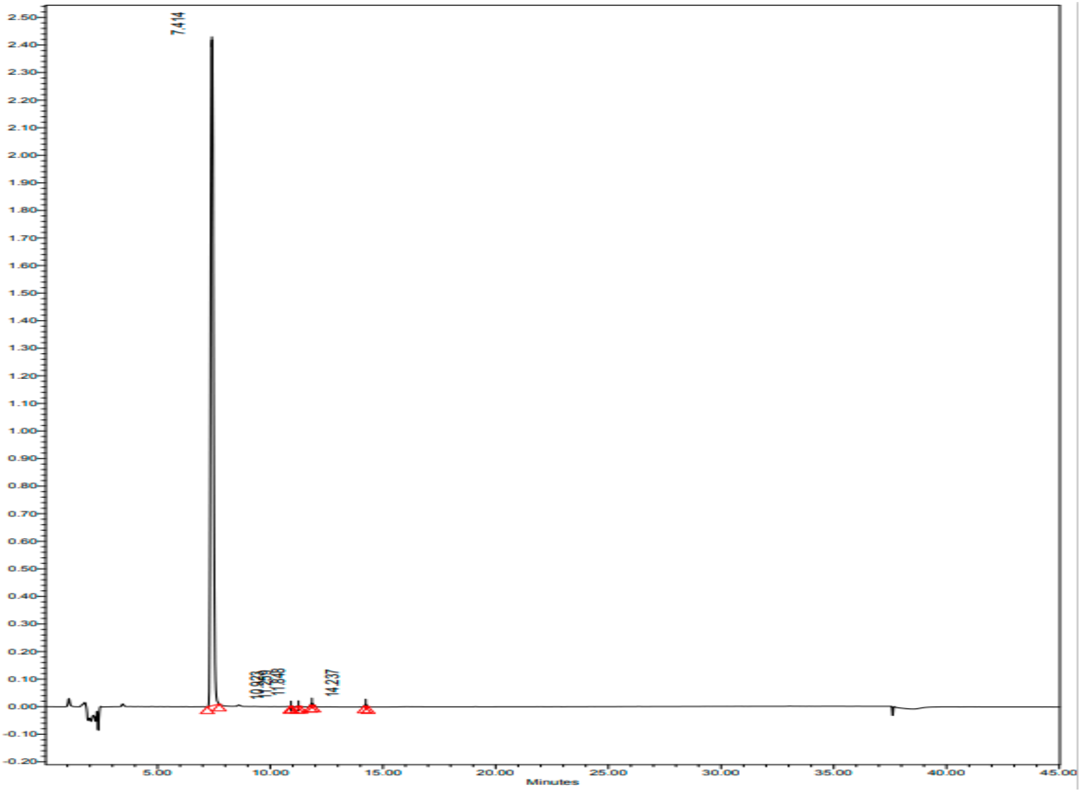
7.414
14.237
Minutes

	Name	Retention Time (min)	Purity1 Angle	Purity1 Threshold	PDA Match1 Spect. Name	PDA Match1 Angle	PDA Match1 Threshold	PDA Match1 Lib. Name	Area (µV*sec)	% Area	Height (µV)	Int Type	Amount
1		7.414							21479454	99.60	2437268	bb	
2		10.923							567	0.00	295	bb	
3		11.259							10194	0.05	1921	bb	
4		11.848							35220	0.16	7485	bb	
5		14.237							39324	0.18	7328	bb	

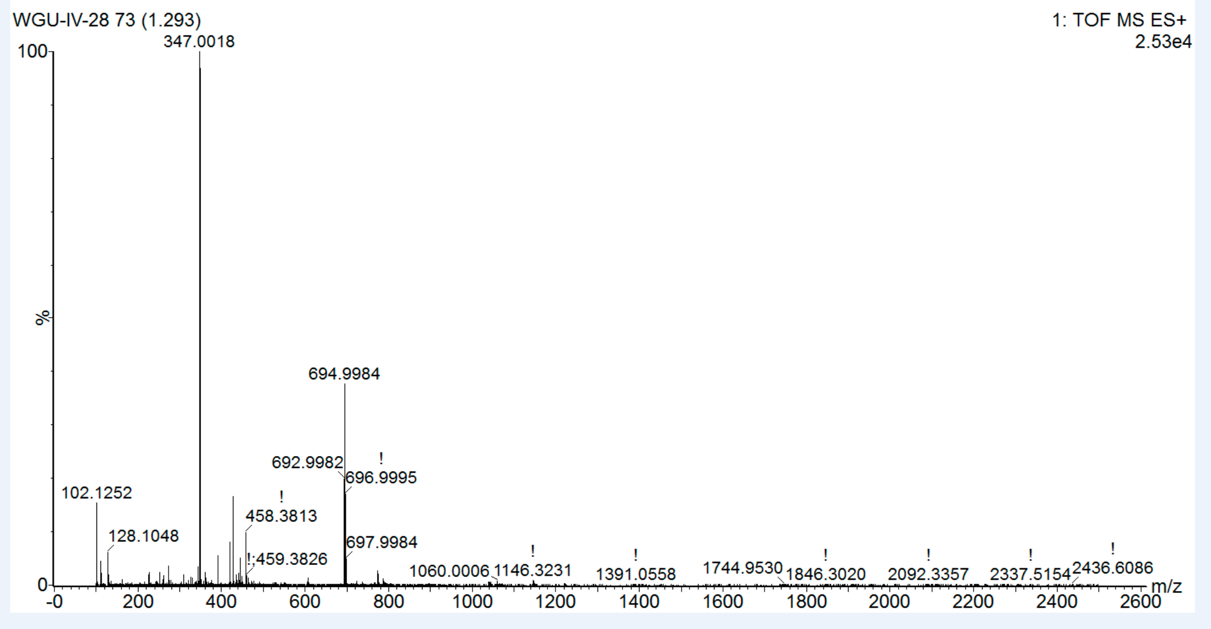

Compound **3d** - (^{1}H NMR, [13] C NMR, HPLC, and HRMS data charts)

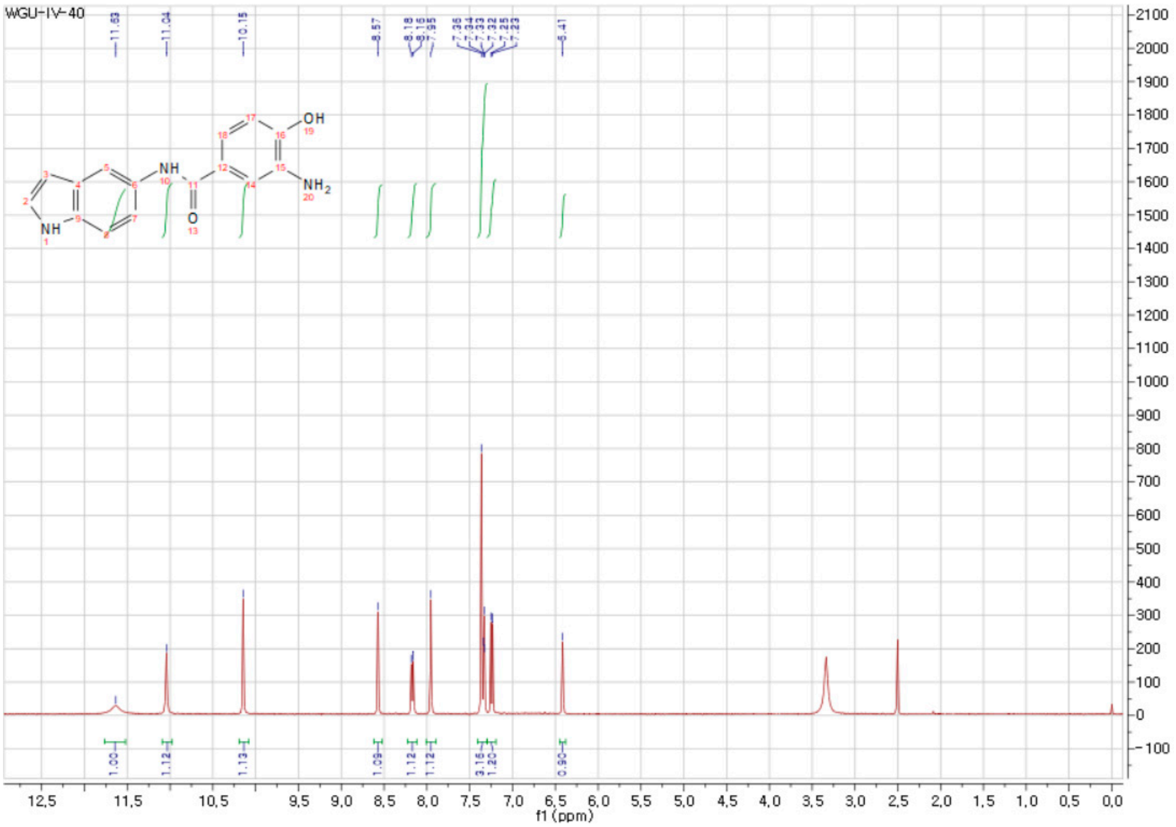

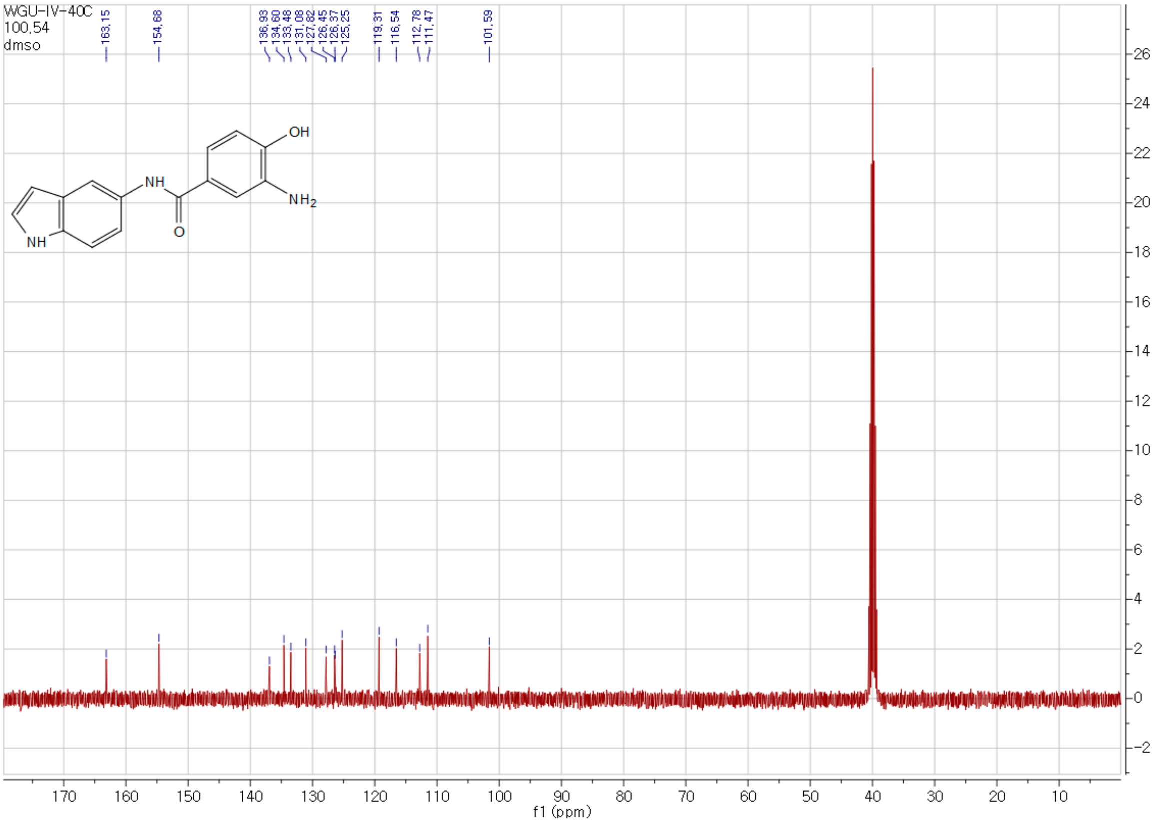

WGU-IV-40C
100.54
dmso
163.15
154.68
136.93
134.60
133.48
131.08
127.82
126.45
126.37
125.25
119.31
116.54
112.78
111.47
101.59
OH
NH
NH2
NH
O
f1 (ppm)

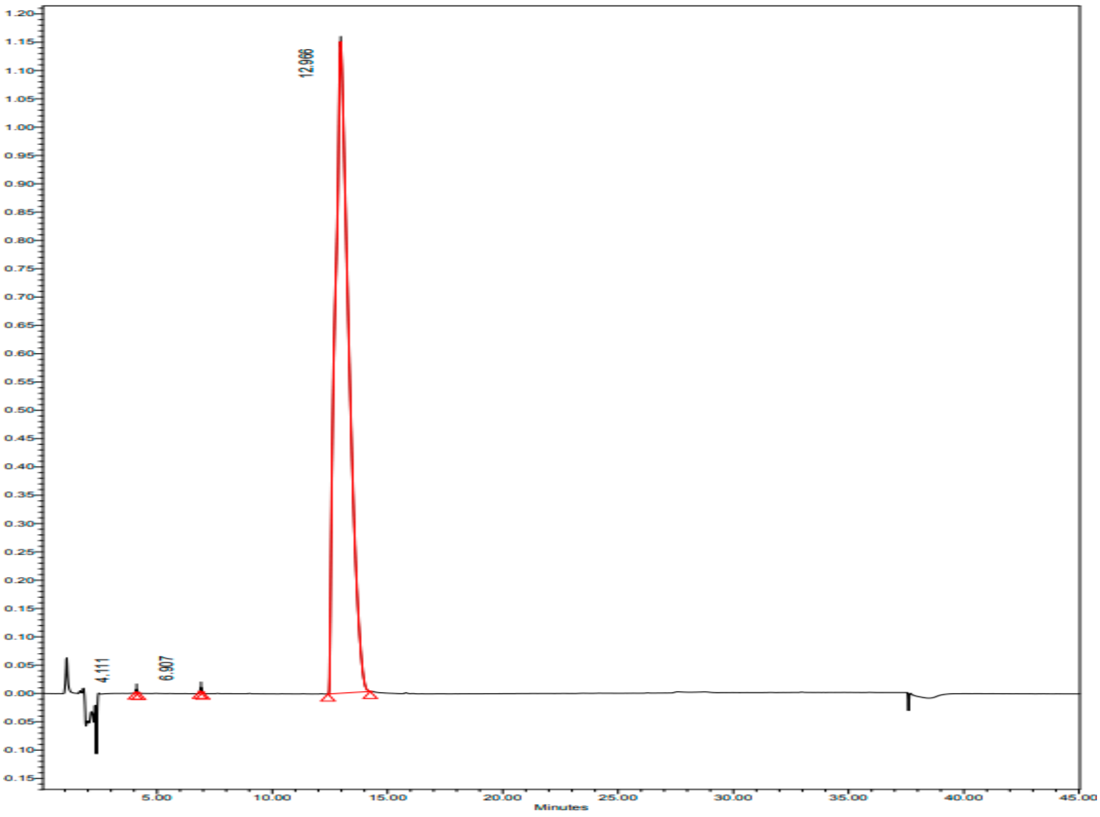

12.966
4.111
6.907
Minutes

	Name	Retention Time (min)	Purity1 Angle	Purity1 Threshold	PDA Match1 Spect. Name	PDA Match1 Angle	PDA Match1 Threshold	PDA Match1 Lib. Name	Area (µV*sec)	% Area	Height (µV)	Int Type	Amount
1		4.111							28208	0.06	5651	bb	
2		6.907							36475	0.08	7958	bb	
3		12.966							47974942	99.87	1150830	bb	

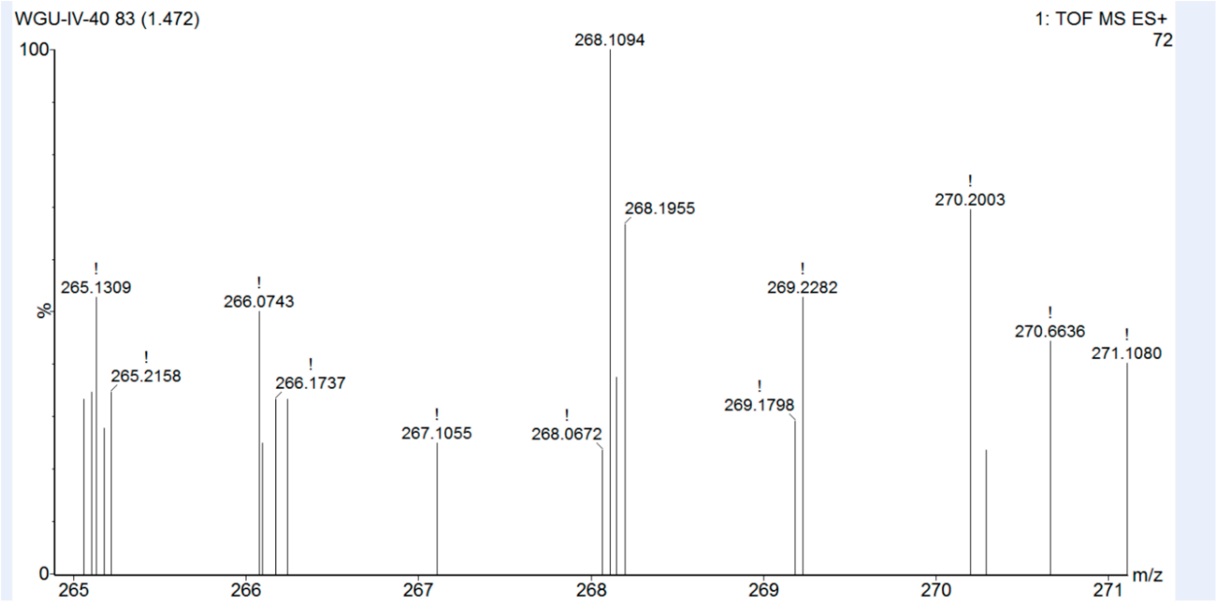

Compound **3e** - (^{1}H NMR, ^{13} C NMR, HPLC, and HRMS data charts)

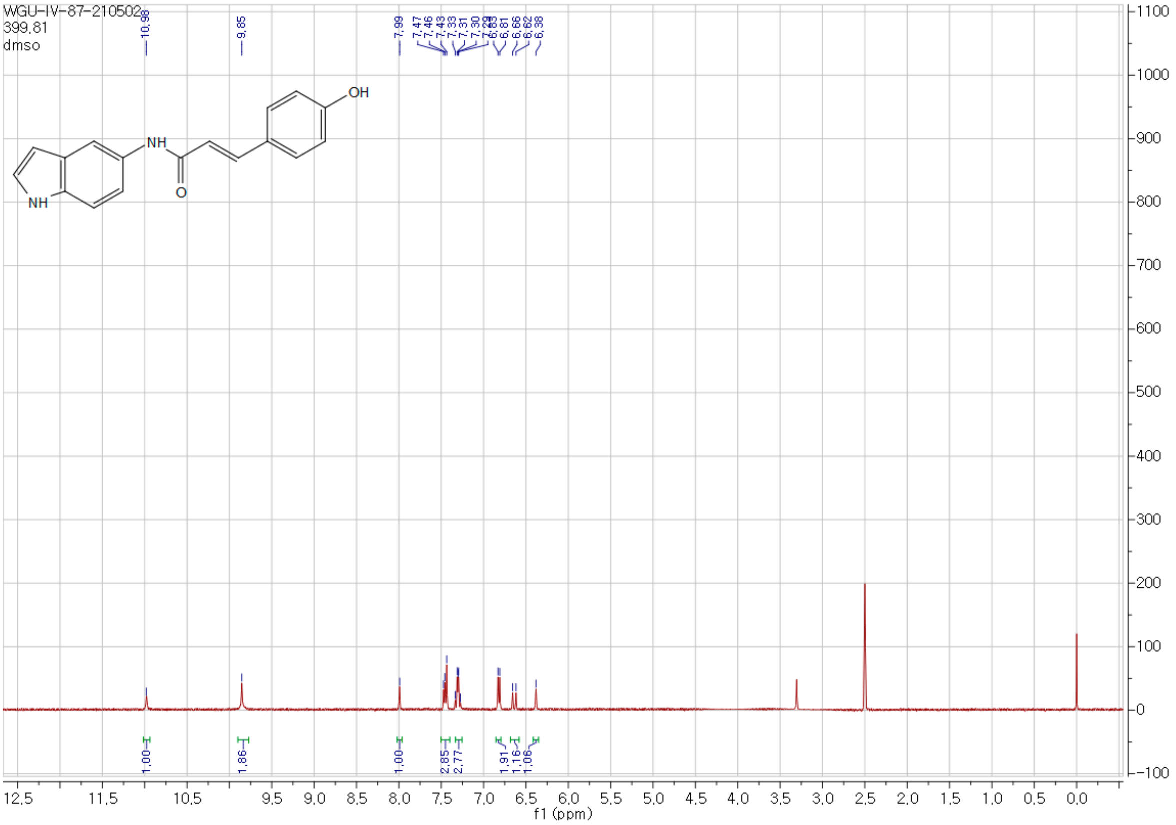

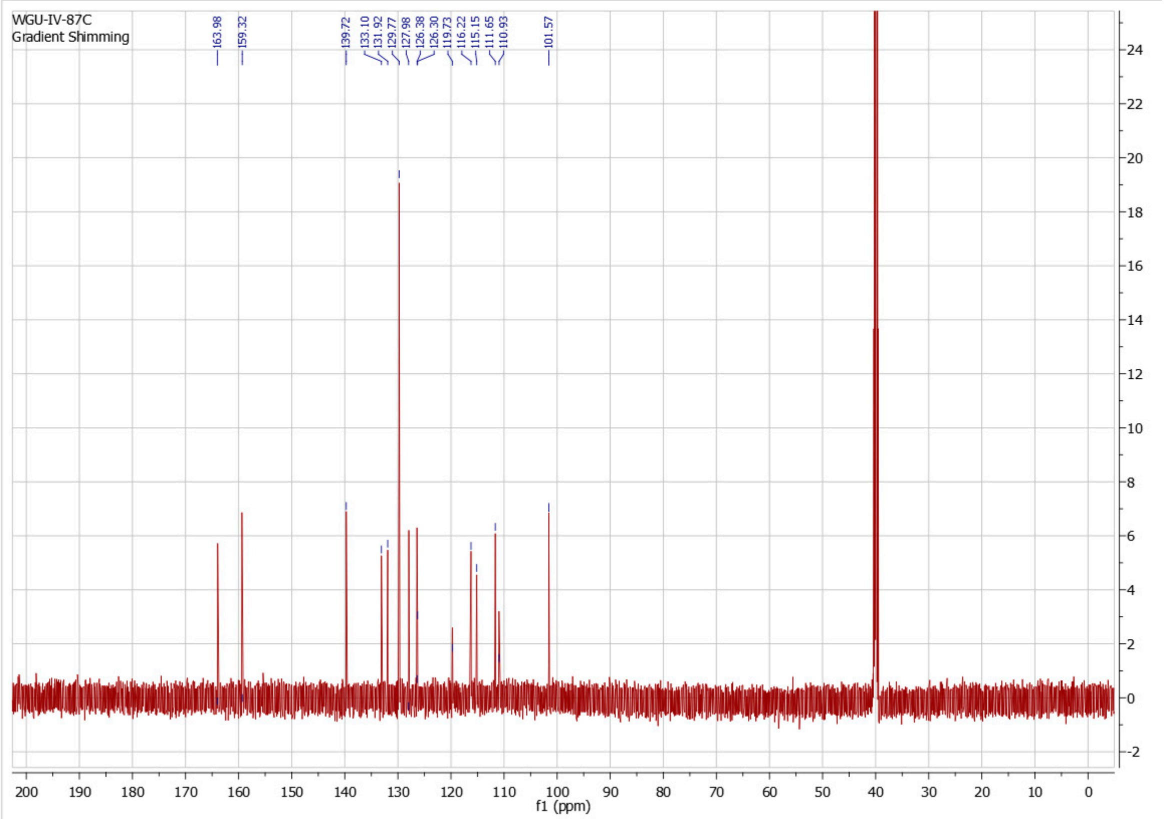
WGU-IV-87C
Gradient Shimming
163.98
159.32
139.72
133.10
131.92
129.77
127.98
126.38
126.30
119.73
116.22
115.15
111.65
110.93
101.57
f1 (ppm)

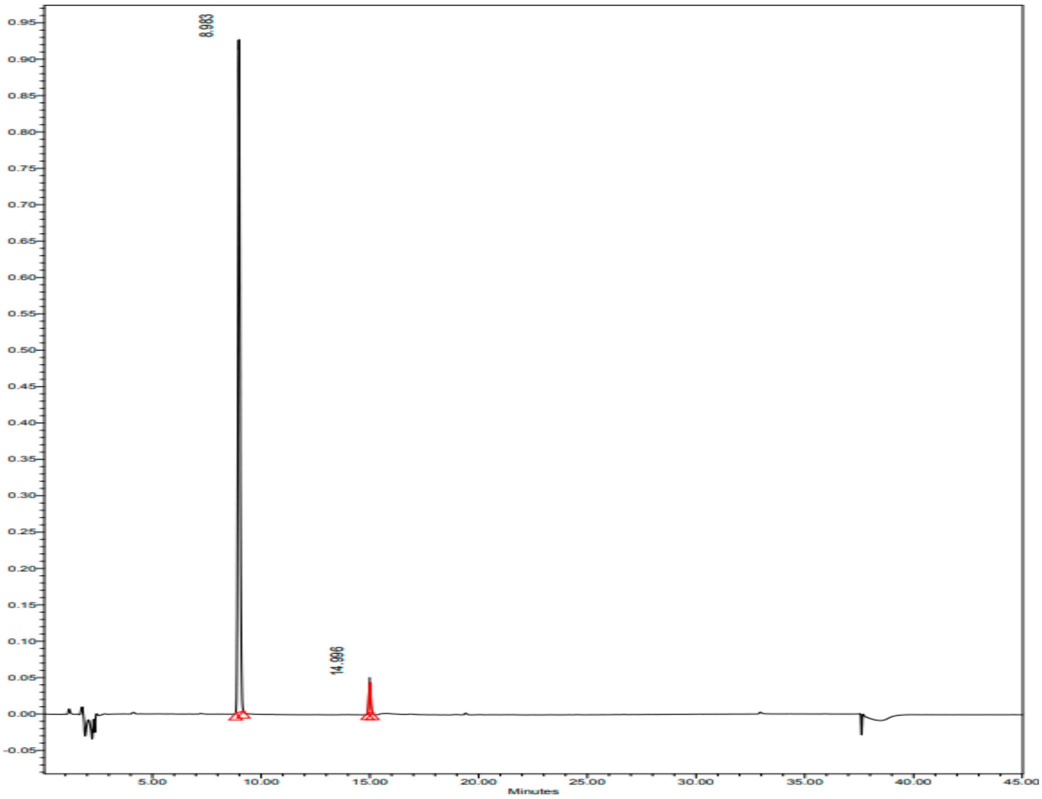
8.983
14.996
Minutes

	Name	Retention Time (min)	Purity1 Angle	Purity1 Threshold	PDA Match1 Spect. Name	PDA Match1 Angle	PDA Match1 Threshold	PDA Match1 Lib. Name	Area (µV*sec)	% Area	Height (µV)	Int Type	Amount	Units
1		8.983							6278950	96.06	920318	bb		
2		14.996							257420	3.94	41613	bb		

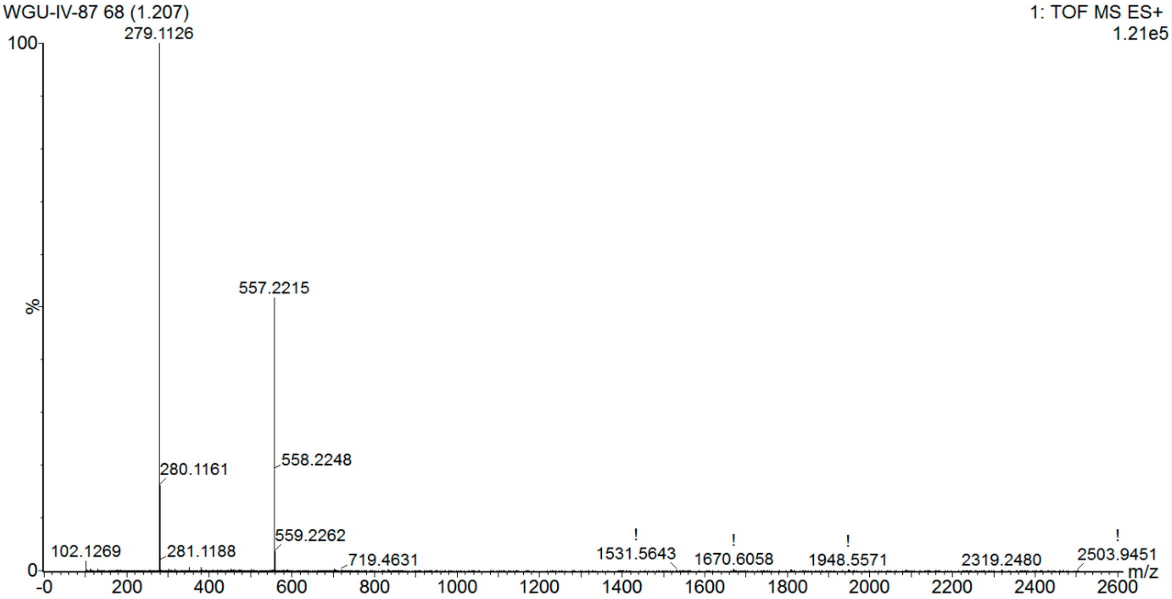

Monoisotopic Mass, Even Electron Ions
399 formula(e) evaluated with 8 results within limits (all results (up to 1000)) for each mass)
Elements Used:
C: 0-500 H: 0-1000 N: 0-10 O: 0-20

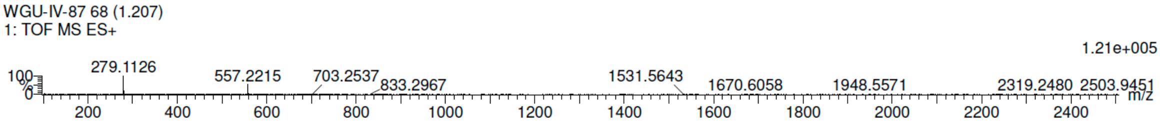

Minimum:				-1.5				
Maximum:		5.0	10.0	50.0				
Mass	Calc. Mass	mDa	PPM	DBE	i-FIT	Norm	Conf(%)	Formula
279.1126	279.1134	-0.8	-2.9	11.5	124.7	0.485	61.55	C17 H15 N2 O2
	279.1093	3.3	11.8	7.5	125.4	1.162	31.30	C12 H15 N4 O4
	279.1107	1.9	6.8	12.5	126.9	2.690	6.79	C13 H11 N8
	279.1080	4.6	16.5	2.5	130.1	5.910	0.27	C11 H19 O8
	279.1174	-4.8	-17.2	15.5	131.4	7.127	0.08	C22 H15
	279.1165	-3.9	-14.0	3.5	133.9	9.697	0.01	C6 H15 N8 O5
	279.1152	-2.6	-9.3	-1.5	134.1	9.911	0.00	C5 H19 N4 O9
	279.1125	0.1	0.4	-0.5	138.5	14.274	0.00	C H15 N10 O7

Compound **3f** - (^{1}H NMR, ^{13} C NMR, HPLC, and HRMS data charts)

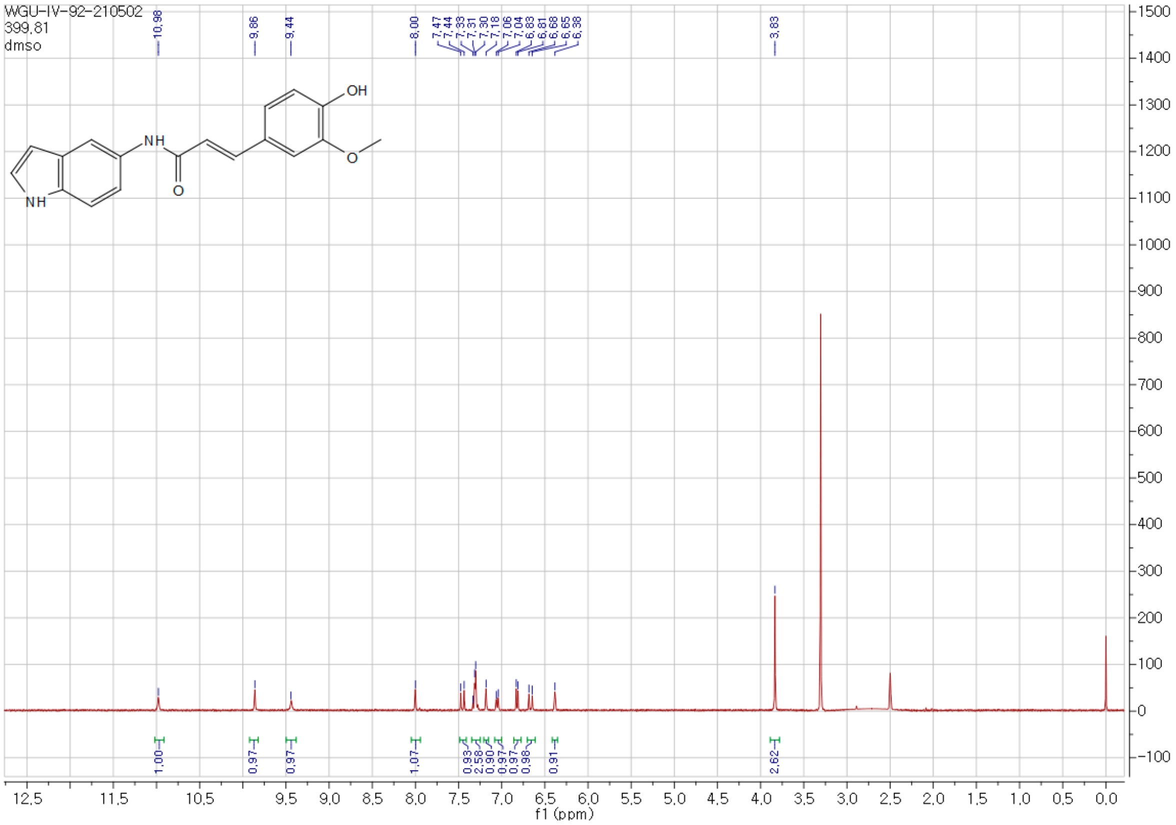

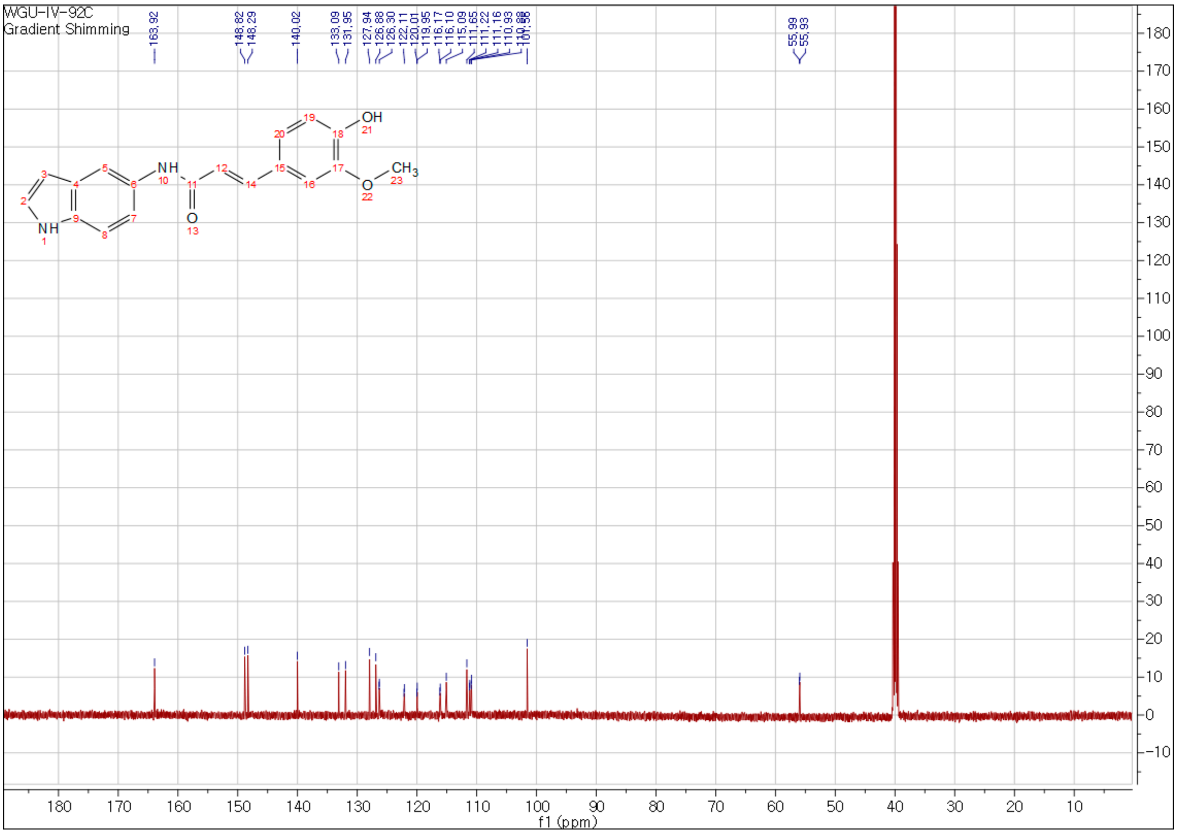
WGU-IV-92C
Gradient Shimming
163.92
148.82
148.29
140.02
133.09
131.95
127.94
126.88
126.30
122.11
120.01
119.95
116.17
116.10
115.09
111.65
111.22
111.16
110.93
55.99
55.93
f1 (ppm)

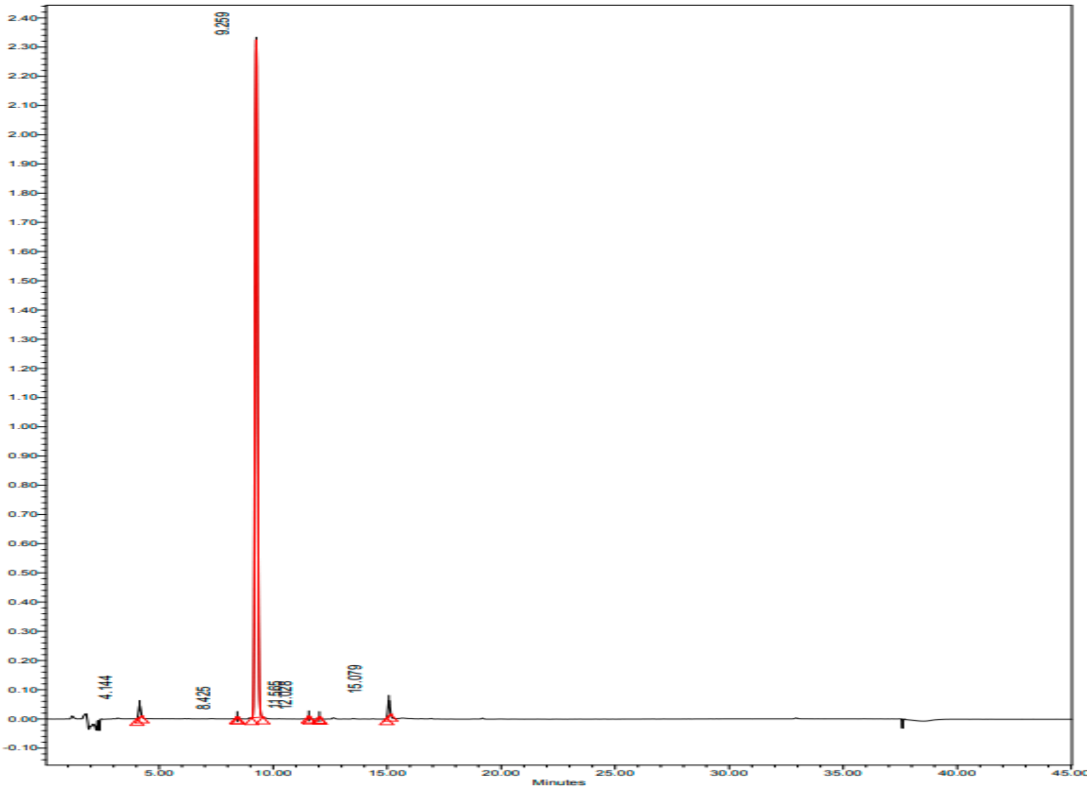
9.259
4.144
8.425
11.565
12.028
15.079
Minutes

	Name	Retention Time (min)	Purity1 Angle	Purity1 Threshold	PDA Match1 Spect. Name	PDA Match1 Angle	PDA Match1 Threshold	PDA Match1 Lib. Name	Area (µV*sec)	% Area	Height (µV)	Int Type	Amount
1		4.144							253536	1.32	39912	bb	
2		8.425							4607	0.02	1835	bb	
3		9.259							18631137	97.06	2328181	bb	
4		11.565							5300	0.03	1811	bb	
5		12.028							1556	0.01	863	bb	
6		15.079							298883	1.56	52927	bb	

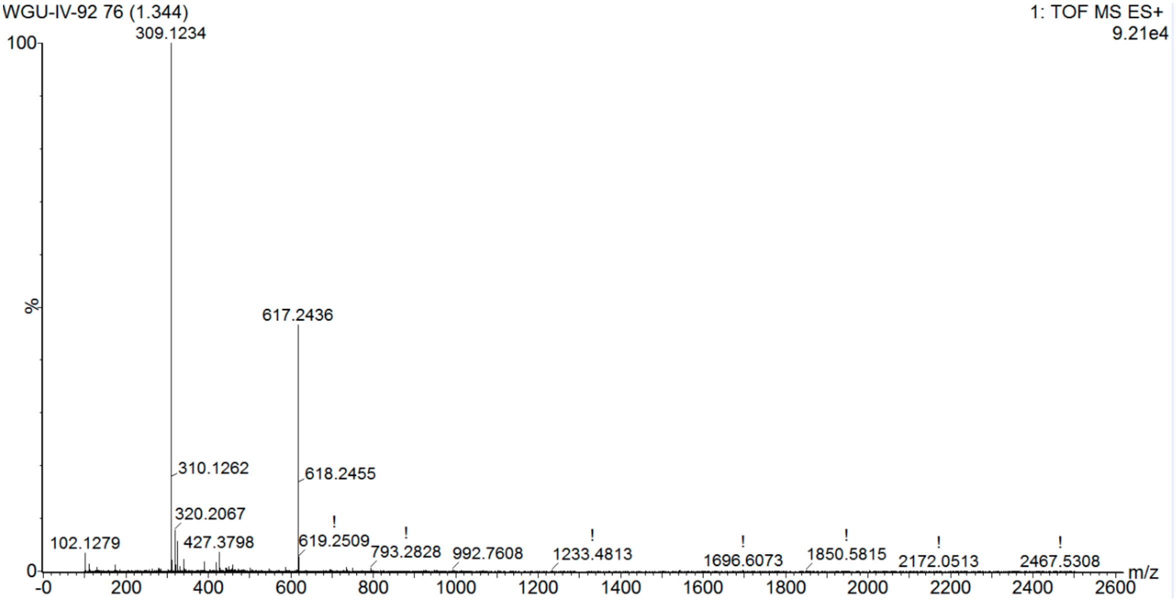

Monoisotopic Mass, Even Electron Ions
483 formula(e) evaluated with 8 results within limits (all results (up to 1000) for each mass)
Elements Used:
C: 0-500 H: 0-1000 N: 0-10 O: 0-20
WGU-IV-92 76 (1.344)
1: TOF MS ES+

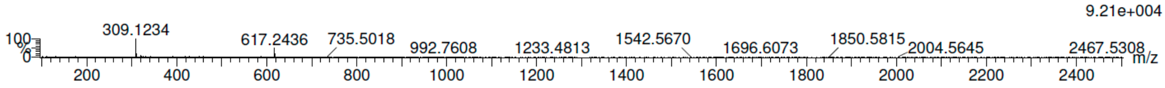

		mDa	PPM	DBE				
Minimum:				-1.5				
Maximum:		5.0	10.0	50.0				

Mass	Calc. Mass	mDa	PPM	DBE	i-FIT	Norm	Conf(%)	Formula
309.1234	309.1212	2.2	7.1	12.5	210.8	0.239	78.76	C14 H13 N8 O
	309.1239	-0.5	-1.6	11.5	212.6	2.028	13.16	C18 H17 N2 O3
	309.1199	3.5	11.3	7.5	213.1	2.523	8.02	C13 H17 N4 O5
	309.1186	4.8	15.5	2.5	218.3	7.703	0.05	C12 H21 O9
	309.1279	-4.5	-14.6	15.5	219.8	9.162	0.01	C23 H17 O
	309.1271	-3.7	-12.0	3.5	220.3	9.691	0.01	C7 H17 N8 O6
	309.1258	-2.4	-7.8	-1.5	220.5	9.933	0.00	C6 H21 N4 O10
	309.1231	0.3	1.0	-0.5	224.6	14.019	0.00	C2 H17 N10 O8

Compound **3g** - (^{1}H NMR, ^{13} C NMR, HPLC, and HRMS data charts)

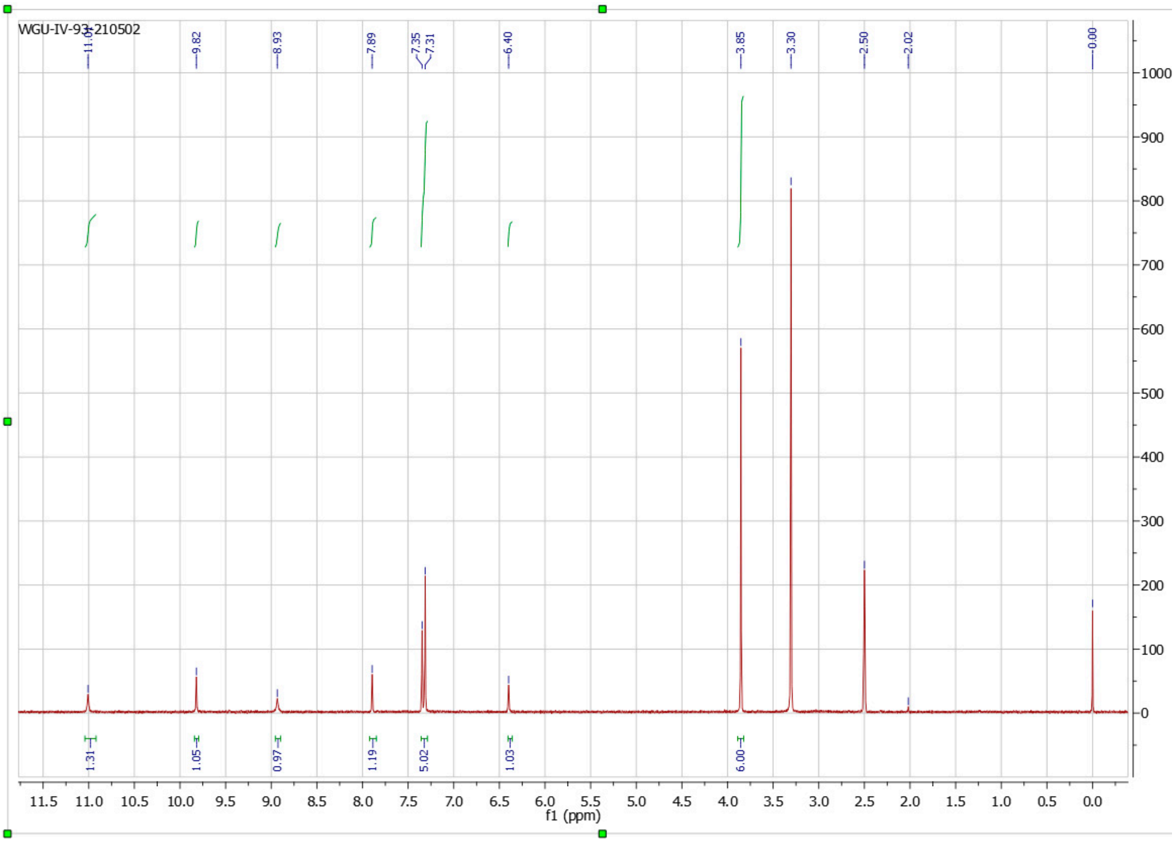

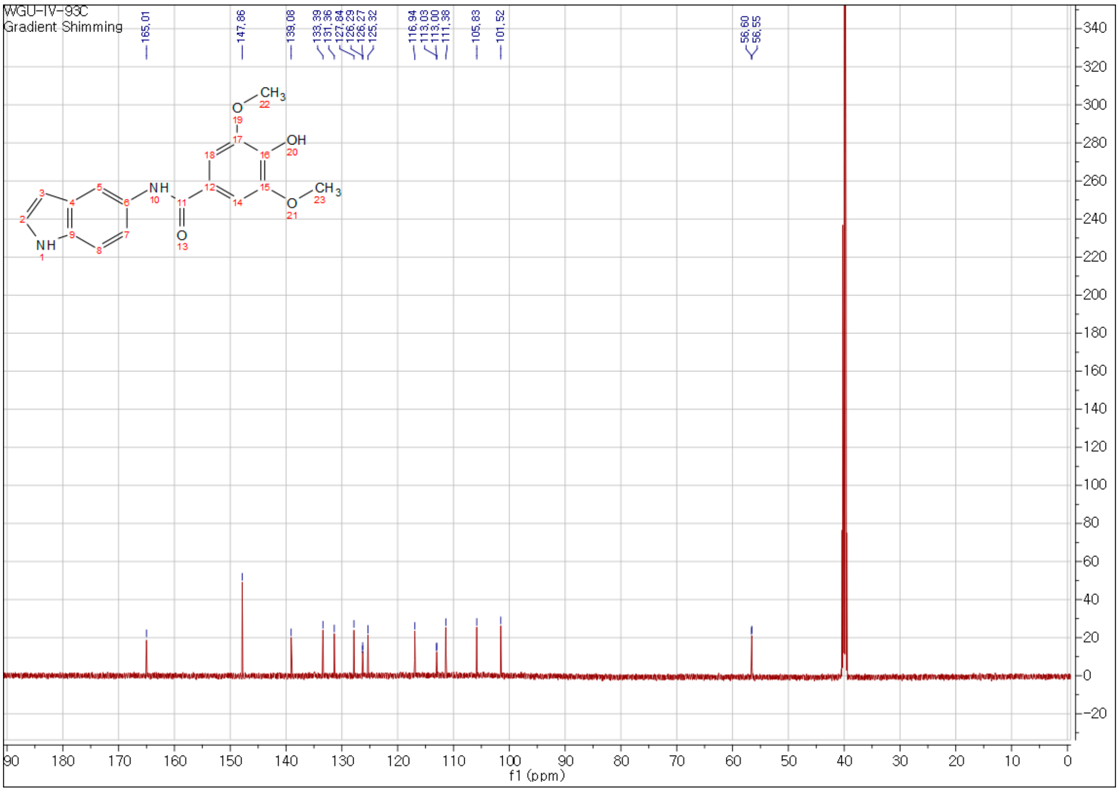

WGU-IV-93C
Gradient Shimming
165.01
147.86
139.08
133.39
131.36
127.84
126.29
126.27
125.32
116.94
113.03
113.00
111.38
105.83
101.52
56.60
56.55
f1 (ppm)

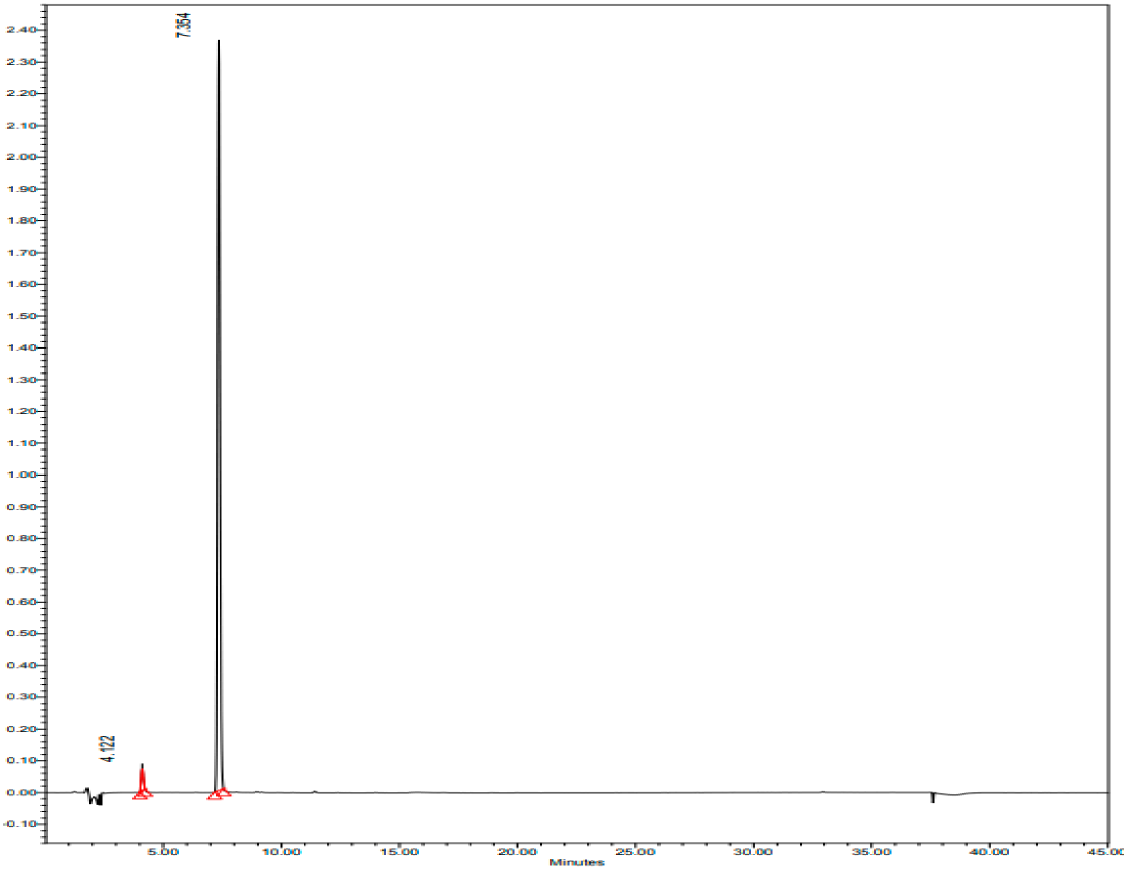

7.354
4.122
Minutes

	Name	Retention Time (min)	Purity1 Angle	Purity1 Threshold	PDA Match1 Spect. Name	PDA Match1 Angle	PDA Match1 Threshold	PDA Match1 Lib. Name	Area (µV*sec)	% Area	Height (µV)	Int Type	Amount
1		4.122							437452	2.36	66851	bb	
2		7.354							18098341	97.64	2358265	bb	

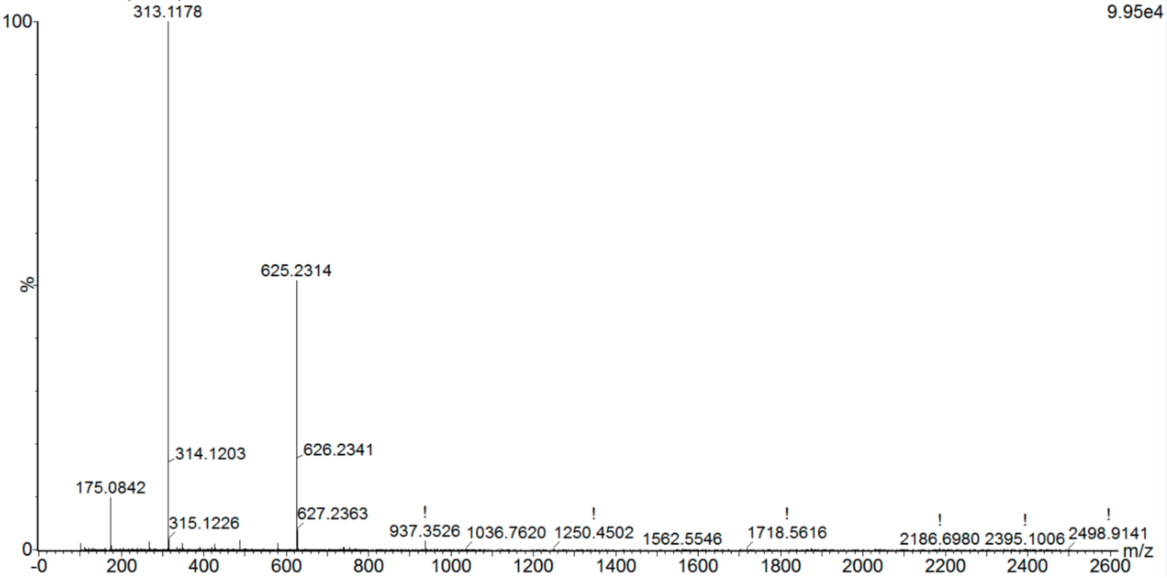

Monoisotopic Mass, Even Electron Ions
496 formula(e) evaluated with 7 results within limits (all results (up to 1000) for each mass)
Elements Used:
C: 0-500 H: 0-1000 N: 0-10 O: 0-20
WGU-IV-93 72 (1.275)
1: TOF MS ES+

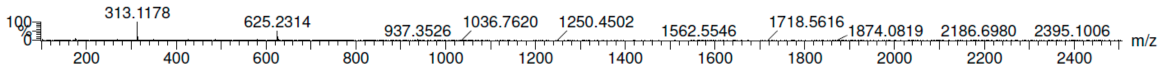

Minimum:				-1.5				
Maximum:		5.0	10.0	50.0				
Mass	Calc. Mass	mDa	PPM	DBE	i-FIT	Norm	Conf(%)	Formula
313.1178	313.1161	1.7	5.4	11.5	143.5	0.069	93.32	C13 H13 N8 O2
	313.1148	3.0	9.6	6.5	146.2	2.741	6.45	C12 H17 N4 O6
	313.1188	-1.0	-3.2	10.5	149.8	6.371	0.17	C17 H17 N2 O4
	313.1202	-2.4	-7.7	15.5	151.2	7.686	0.05	C18 H13 N6
	313.1135	4.3	13.7	1.5	152.5	9.043	0.01	C11 H21 O10
	313.1220	-4.2	-13.4	2.5	153.9	10.392	0.00	C6 H17 N8 O7
	313.1180	-0.2	-0.6	-1.5	160.0	16.504	0.00	C H17 N10 O9

Compound **3h** - (^{1}H NMR, ^{13} C NMR, HPLC, and HRMS data charts)

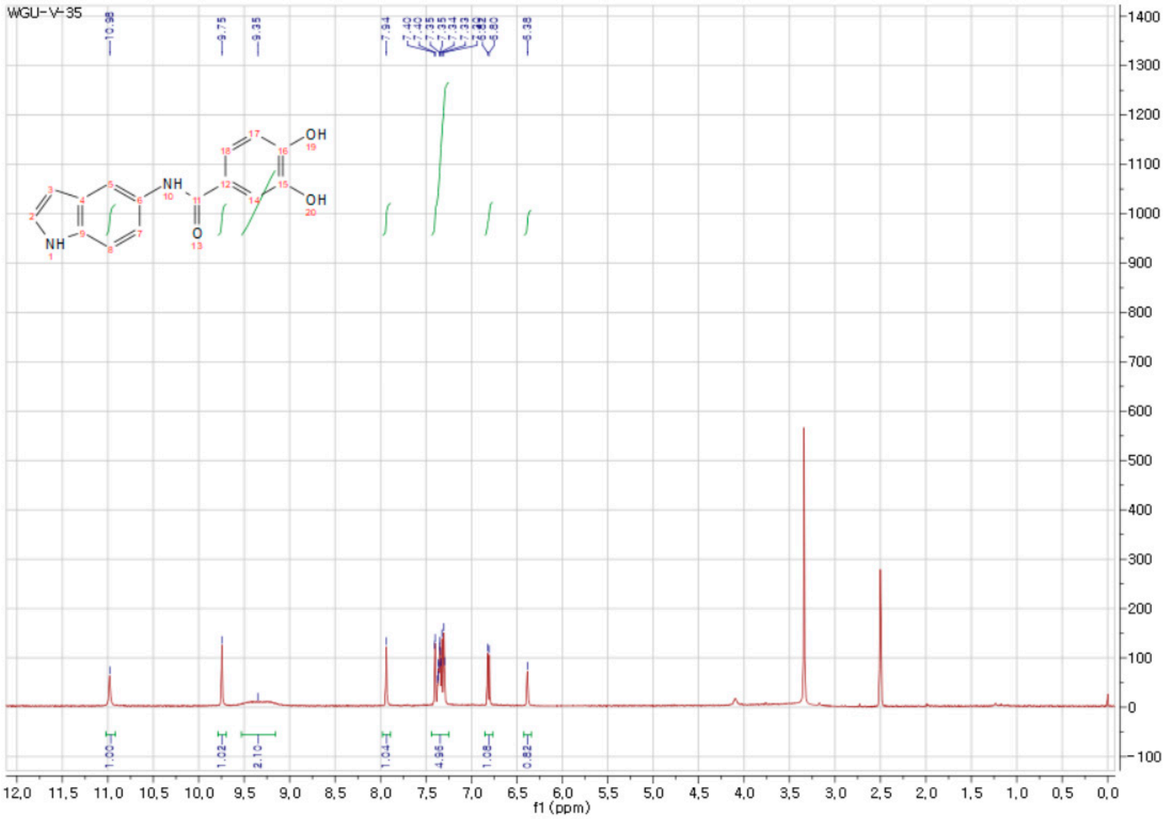

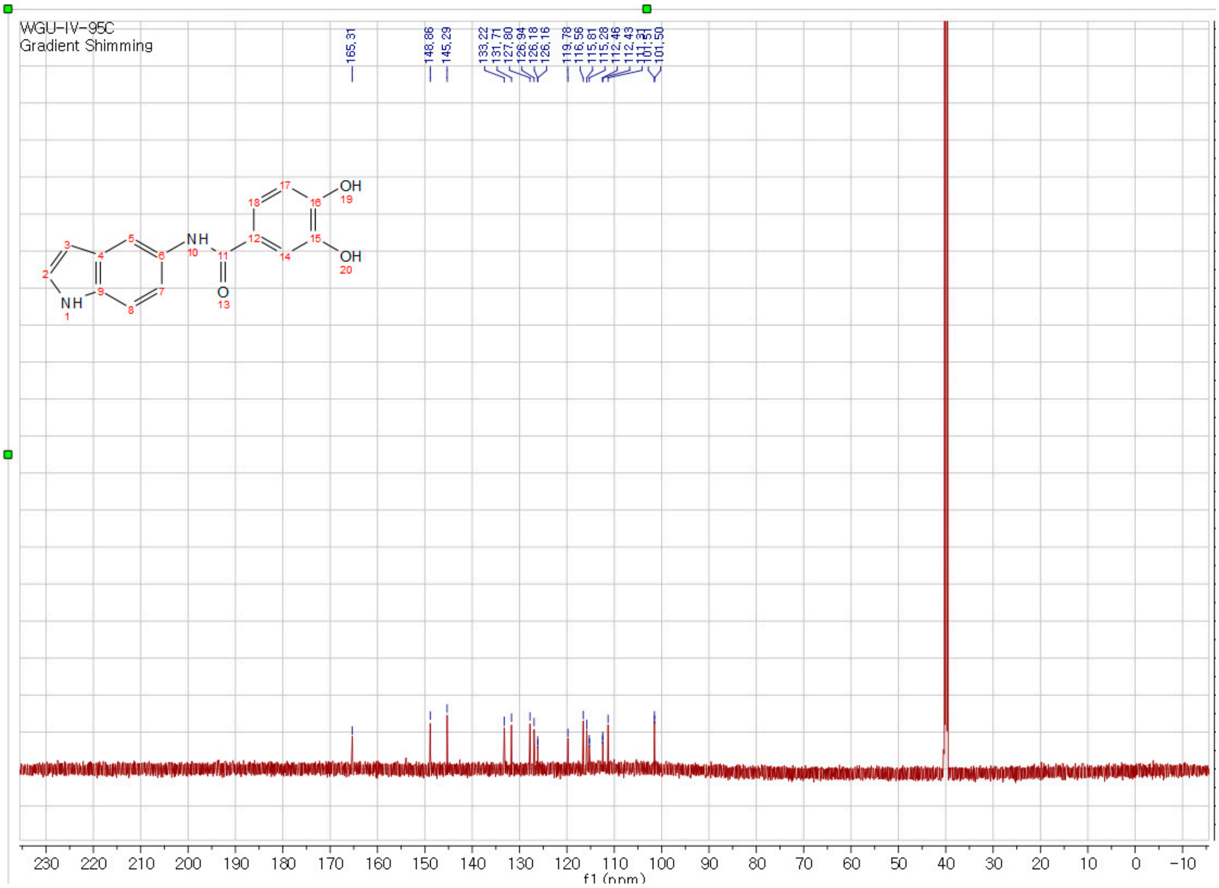
WGU-IV-95C
Gradient Shimming
165.31
148.86
145.29
133.22
131.71
127.80
126.94
126.18
126.16
119.78
116.56
115.81
115.28
112.46
112.43
101.51
101.50
f1 (ppm)

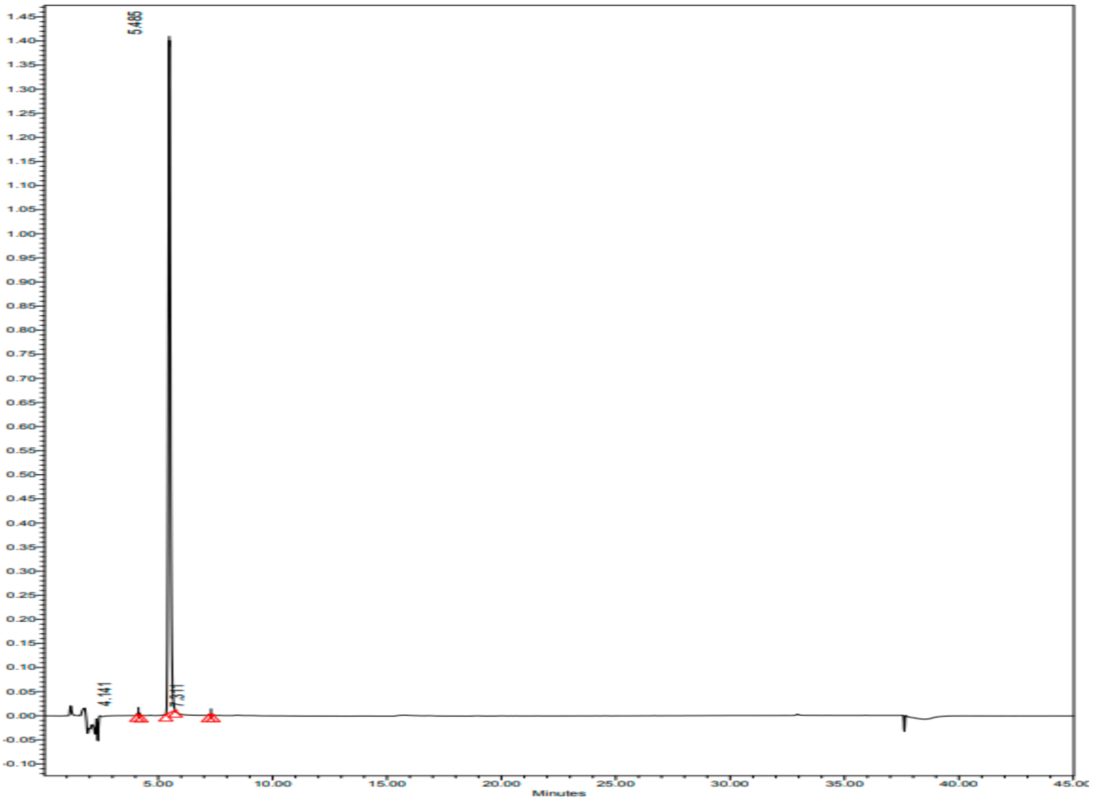

	Name	Retention Time (min)	Purity1 Angle	Purity1 Threshold	PDA Match1 Spect. Name	PDA Match1 Angle	PDA Match1 Threshold	PDA Match1 Lib. Name	Area (µV*sec)	% Area	Height (µV)	Int Type	Amount
1		4.141							28544	0.30	4740	bb	
2		5.485							9351070	99.53	1387600	bb	
3		7.311							15301	0.16	1925	bb	

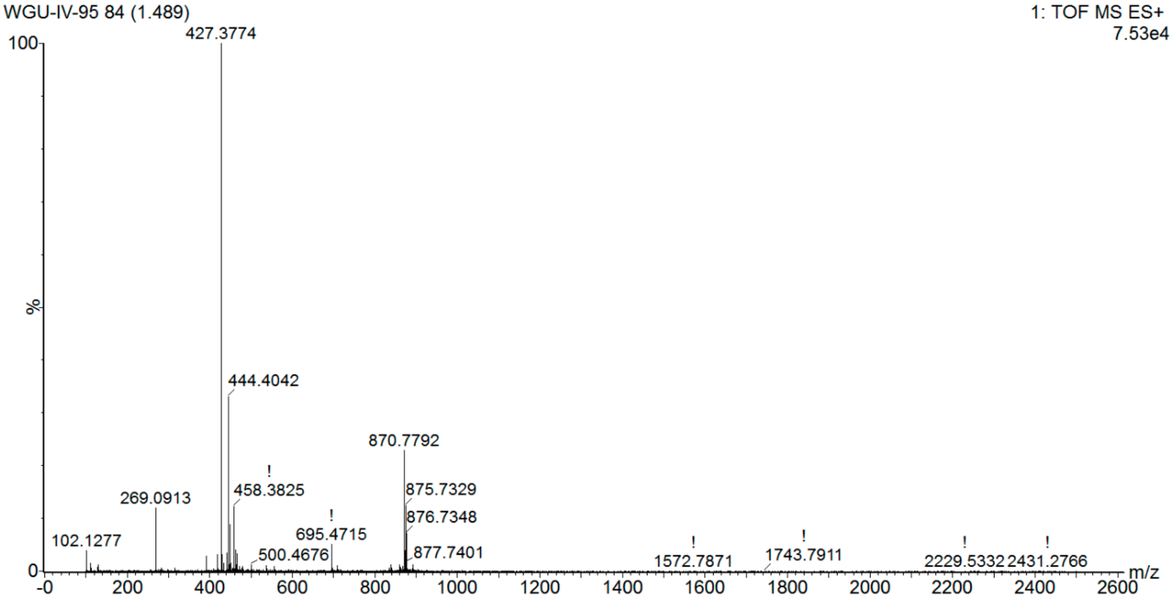

Monoisotopic Mass, Even Electron Ions
375 formula(e) evaluated with 5 results within limits (all results (up to 1000) for each mass)
Elements Used:
C: 0-500 H: 0-1000 N: 0-10 O: 0-20
WGU-IV-95 84 (1.489)
1: TOF MS ES+

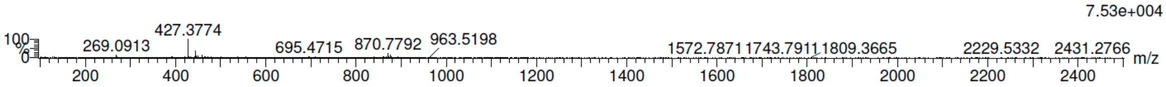

Minimum:				-1.5				
Maximum:		5.0	10.0	50.0				

Mass	Calc. Mass	mDa	PPM	DBE	i-FIT	Norm	Conf(%)	Formula
269.0913	269.0926	-1.3	-4.8	10.5	117.2	0.235	79.04	C15 H13 N2 O3
	269.0899	1.4	5.2	11.5	118.7	1.729	17.75	C11 H9 N8 O
	269.0886	2.7	10.0	6.5	120.4	3.523	2.95	C10 H13 N4 O5
	269.0873	4.0	14.9	1.5	122.9	5.973	0.25	C9 H17 O9
	269.0958	-4.5	-16.7	2.5	126.2	9.254	0.01	C4 H13 N8 O6

Compound **3i** - (^{1}H NMR, ^{13} C NMR, HPLC, and HRMS data charts)

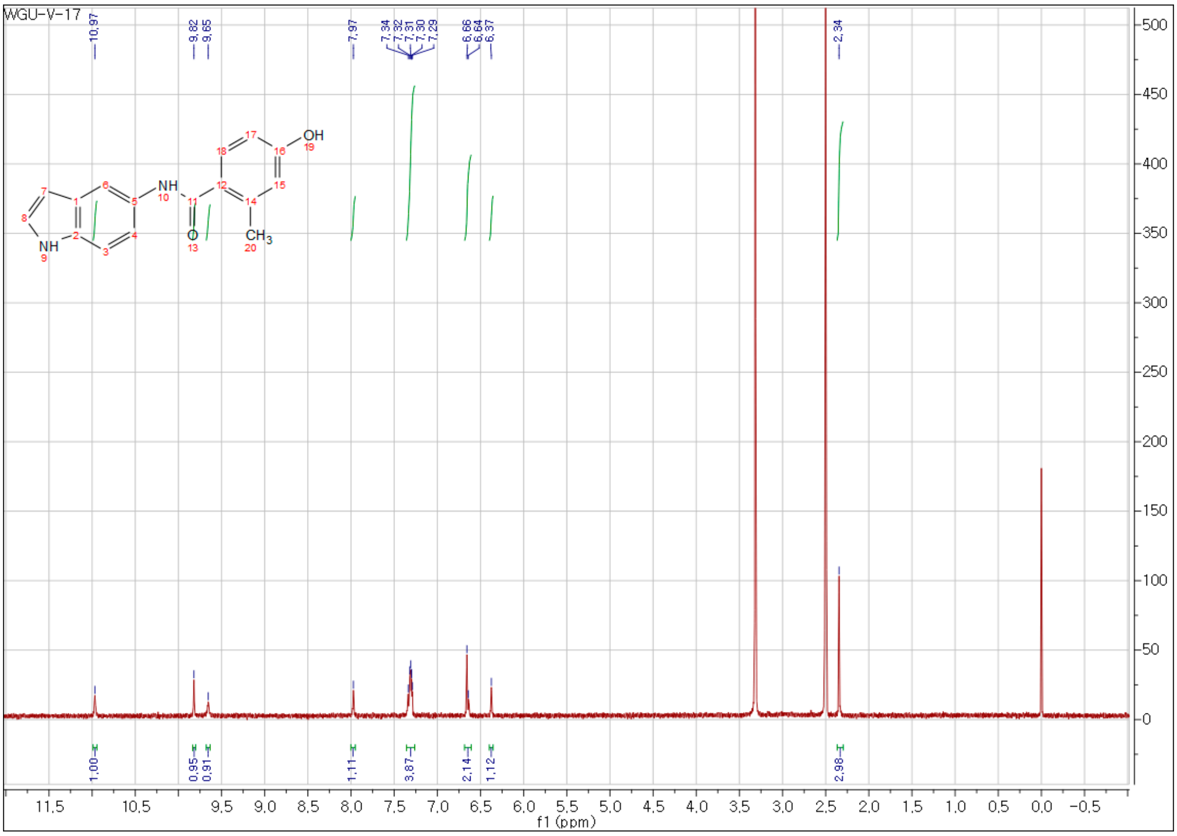

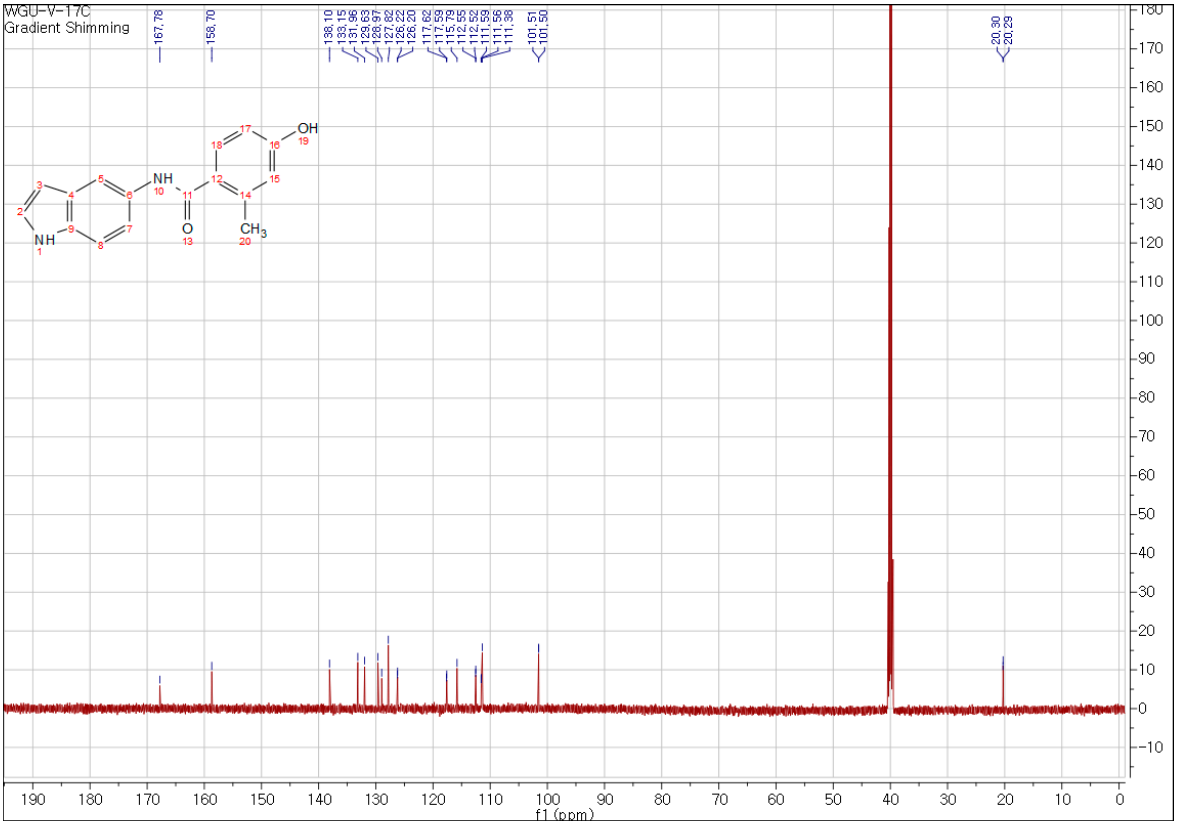
WGU-V-17C
Gradient Shimming
167.78
158.70
138.10
133.15
131.96
129.63
128.97
127.82
126.22
126.20
117.62
117.59
115.79
112.55
112.52
111.59
111.56
111.38
101.51
101.50
20.30
20.29
NH
OH
O
CH3
NH
f1 (ppm)

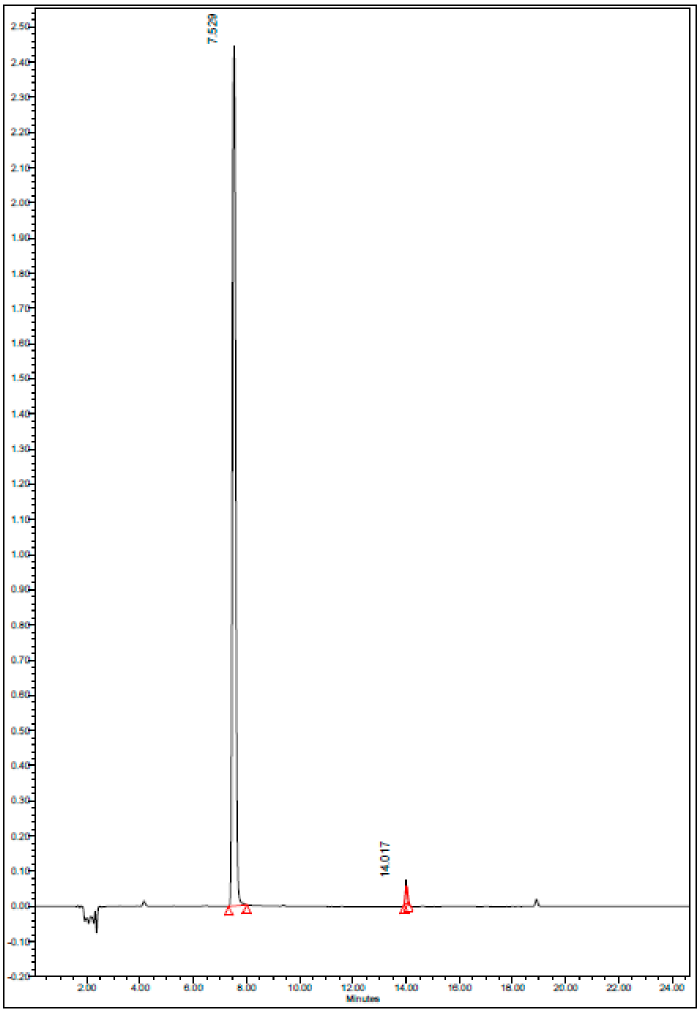

	Name	Retention Time (min)	Purity1 Angle	Purity1 Threshold	PDA Match1 Spect. Name	PDA Match1 Angle	PDA Match1 Threshold	PDA Match1 Lib. Name	Area (µV*sec)	% Area	Height (µV)	Int Type	Amount
1		7.529							20602686	98.76	2439751	bb	
2		14.017							257769	1.24	48605	bb	

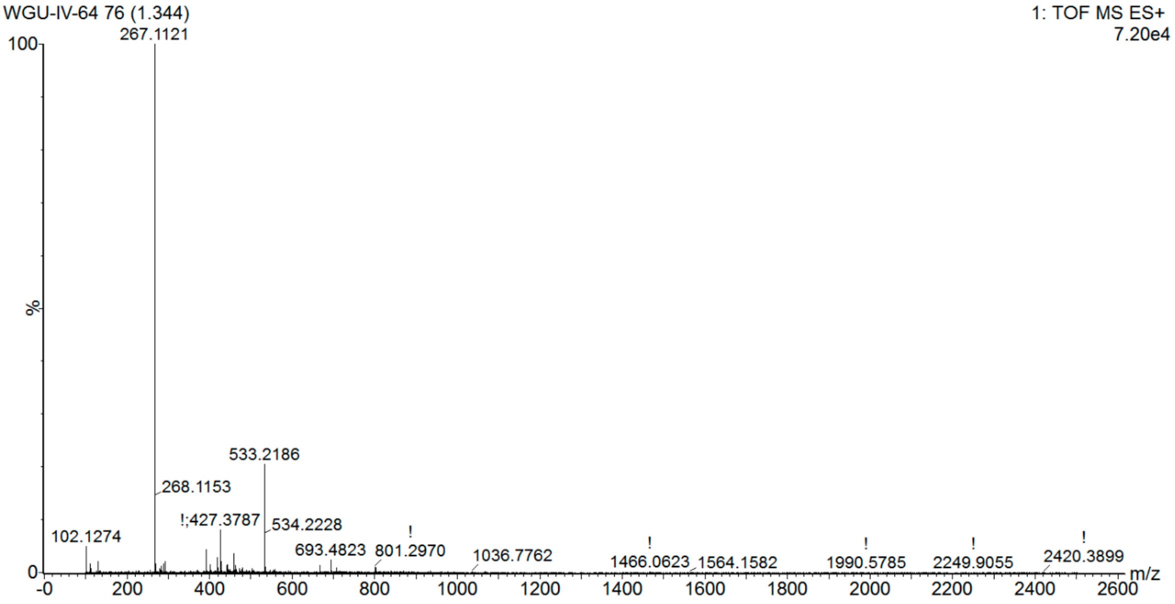

Monoisotopic Mass, Even Electron Ions
369 formula(e) evaluated with 6 results within limits (all results (up to 1000) for each mass)
Elements Used:
C: 0-500   H: 0-1000   N: 0-10   O: 0-20
WGU-IV-64 76 (1.344)
1: TOF MS ES+

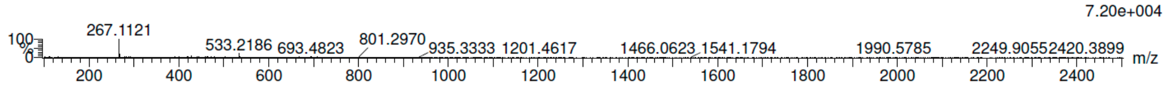

Minimum:				-1.5				
Maximum:		5.0	10.0	50.0				
Mass	Calc. Mass	mDa	PPM	DBE	i-FIT	Norm	Conf(%)	Formula
267.1121	267.1093	2.8	10.5	6.5	110.1	0.021	97.90	C11 H15 N4 O4
	267.1134	-1.3	-4.9	10.5	114.2	4.165	1.55	C16 H15 N2 O2
	267.1107	1.4	5.2	11.5	115.4	5.350	0.47	C12 H11 N8
	267.1080	4.1	15.3	1.5	117.3	7.243	0.07	C10 H19 O8
	267.1165	-4.4	-16.5	2.5	120.5	10.432	0.00	C5 H15 N8 O5
	267.1125	-0.4	-1.5	-1.5	125.4	15.389	0.00	H15 N10 O7

Compound **3j** - (^{1}H NMR, [13] C NMR, HPLC, and HRMS data charts)

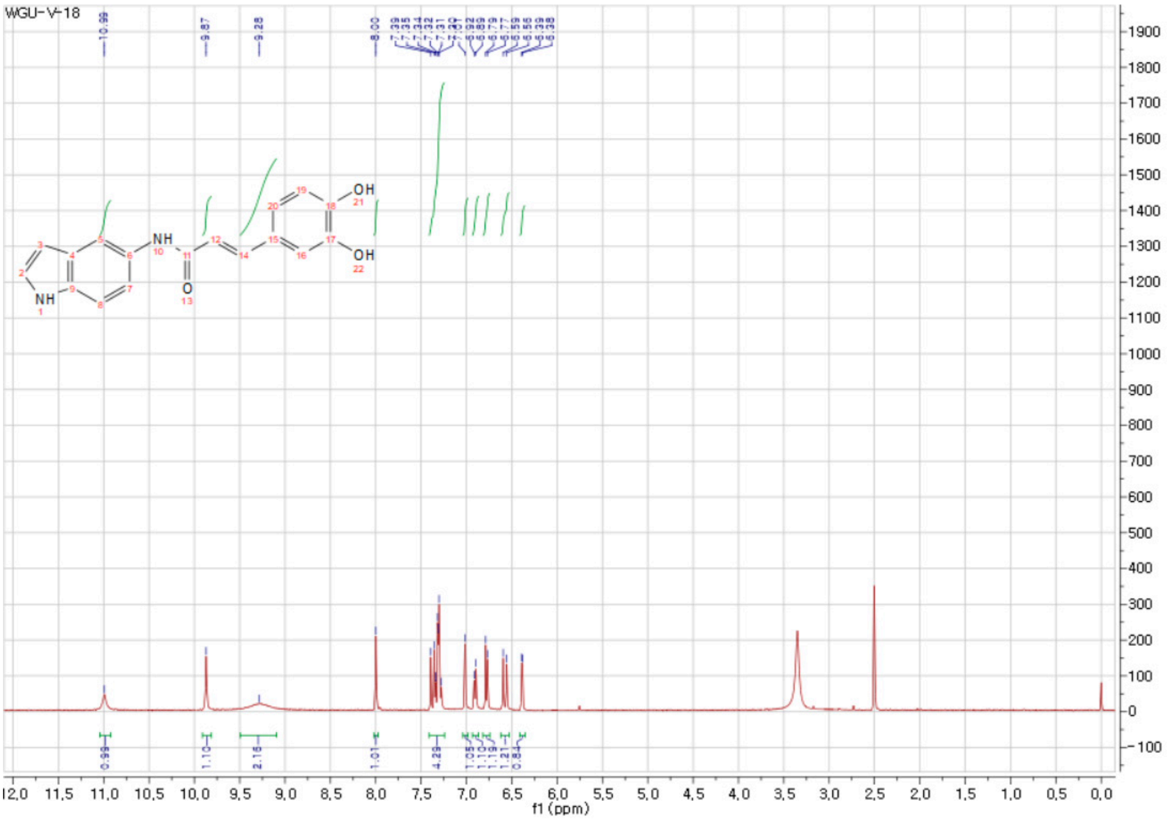

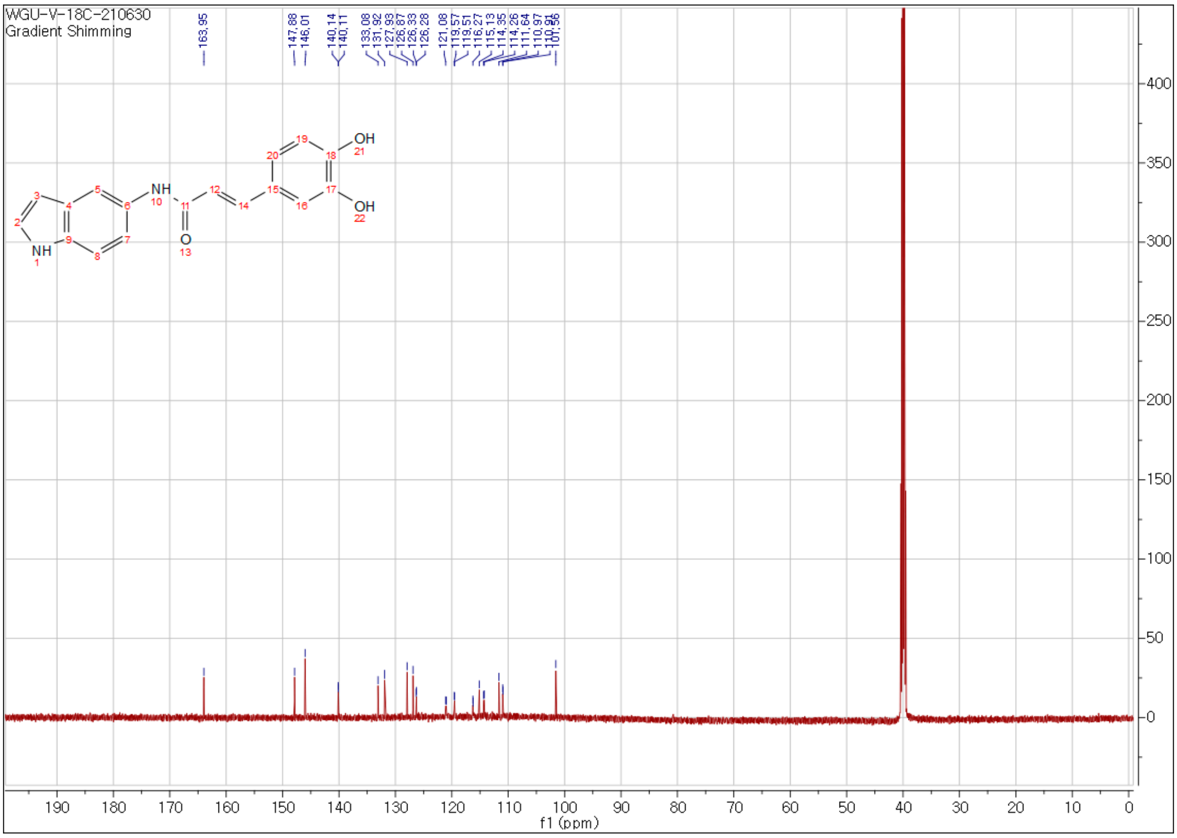

WGU-V-18C-210630
Gradient Shimming
163.95
147.88
146.01
140.14
140.11
133.08
131.92
127.93
126.87
126.33
126.28
121.08
119.57
119.51
116.27
115.13
114.35
114.26
111.64
110.97
110.91
101.56
NH
OH
OH
NH
O
f1 (ppm)

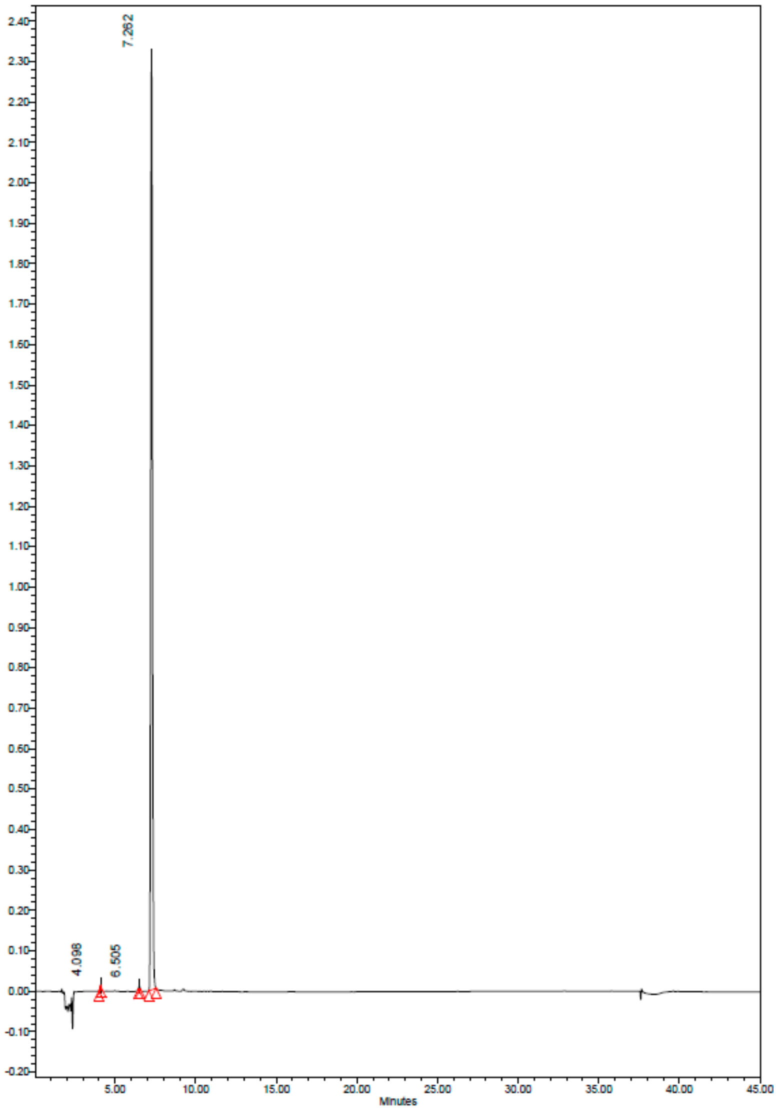

	Name	Retention Time (min)	Purity1 Angle	Purity1 Threshold	PDA Match1 Spect. Name	PDA Match1 Angle	PDA Match1 Threshold	PDA Match1 Lib. Name	Area (µV*sec)	% Area	Height (µV)	Int Type	Amount
1		4.098							41532	0.24	9318	bb	
2		6.505							30212	0.17	7076	bb	
3		7.262							17438940	99.59	2321530	bb	

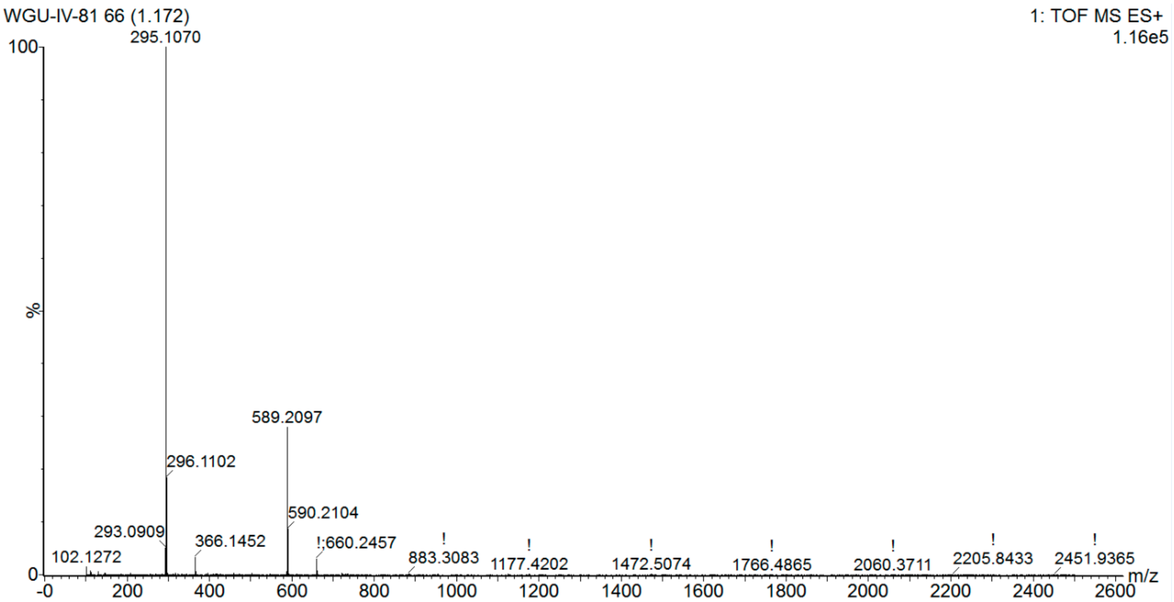

Monoisotopic Mass, Even Electron Ions
445 formula(e) evaluated with 7 results within limits (all results (up to 1000) for each mass)
Elements Used:
C: 0-500 H: 0-1000 N: 0-10 O: 0-20
WGU-IV-81 66 (1.172)
1: TOF MS ES+

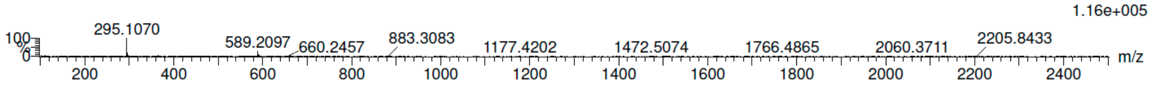

Minimum:				-1.5				
Maximum:		5.0	10.0	50.0				
Mass	Calc. Mass	mDa	PPM	DBE	i-FIT	Norm	Conf(%)	Formula
295.1070	295.1083	-1.3	-4.4	11.5	127.9	0.007	99.32	C17 H15 N2 O3
	295.1056	1.4	4.7	12.5	133.0	5.184	0.56	C13 H11 N8 O
	295.1042	2.8	9.5	7.5	134.7	6.803	0.11	C12 H15 N4 O5
	295.1029	4.1	13.9	2.5	137.8	9.931	0.00	C11 H19 O9
	295.1101	-3.1	-10.5	-1.5	140.8	12.957	0.00	C5 H19 N4 O10
	295.1115	-4.5	-15.2	3.5	141.2	13.314	0.00	C6 H15 N8 O6
	295.1074	-0.4	-1.4	-0.5	145.7	17.810	0.00	C H15 N10 O8

Compound **3k** - (^{1}H NMR, ^{13} C NMR, HPLC, and HRMS data charts)

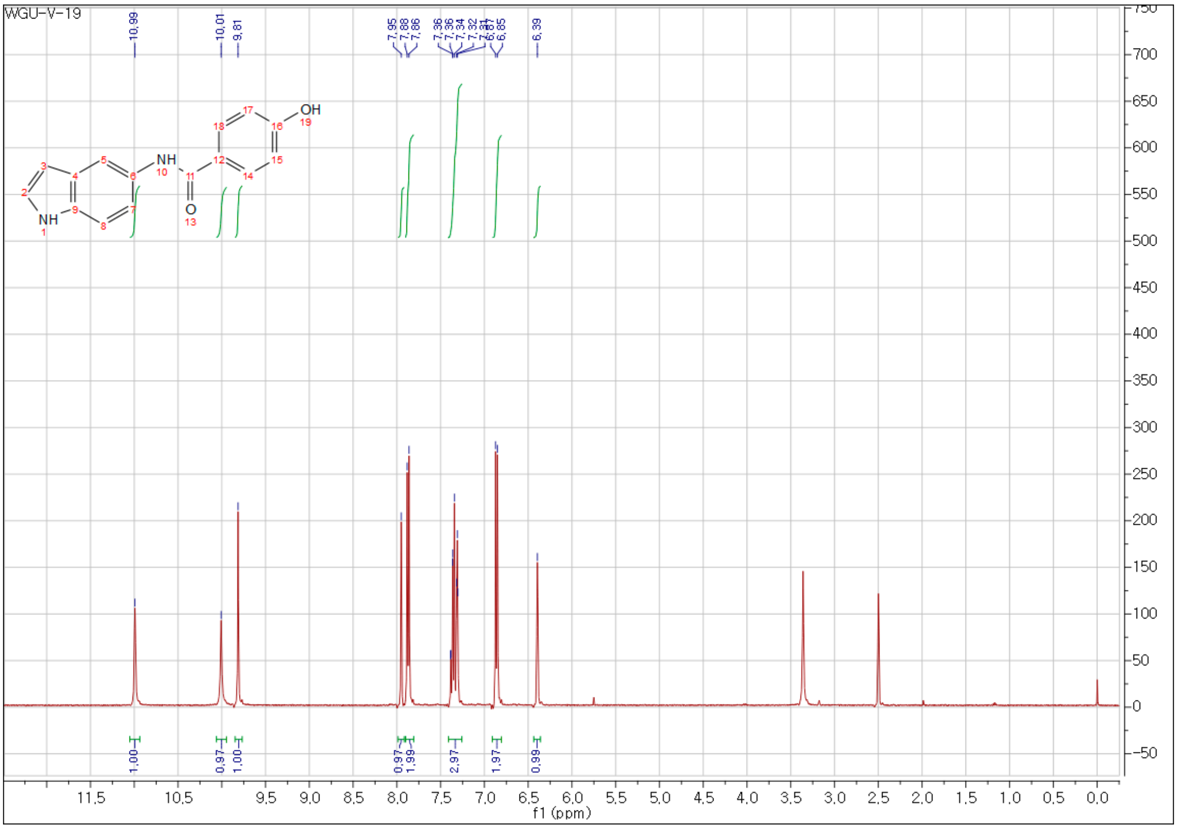

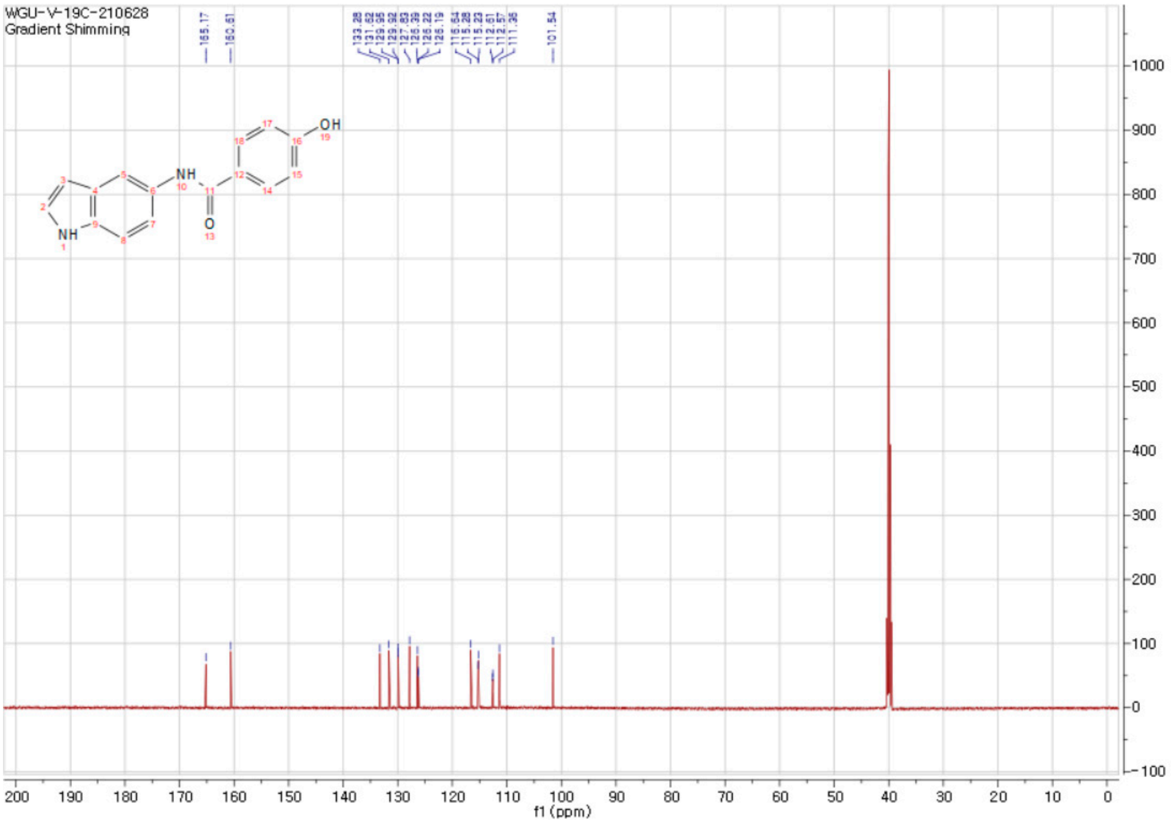
WGU-V-19C-210628
Gradient Shimming
165.17
160.61
133.28
131.62
129.95
129.92
127.83
126.39
126.22
126.19
116.54
115.28
115.23
112.61
112.57
111.36
101.54
OH
NH
O
f1 (ppm)

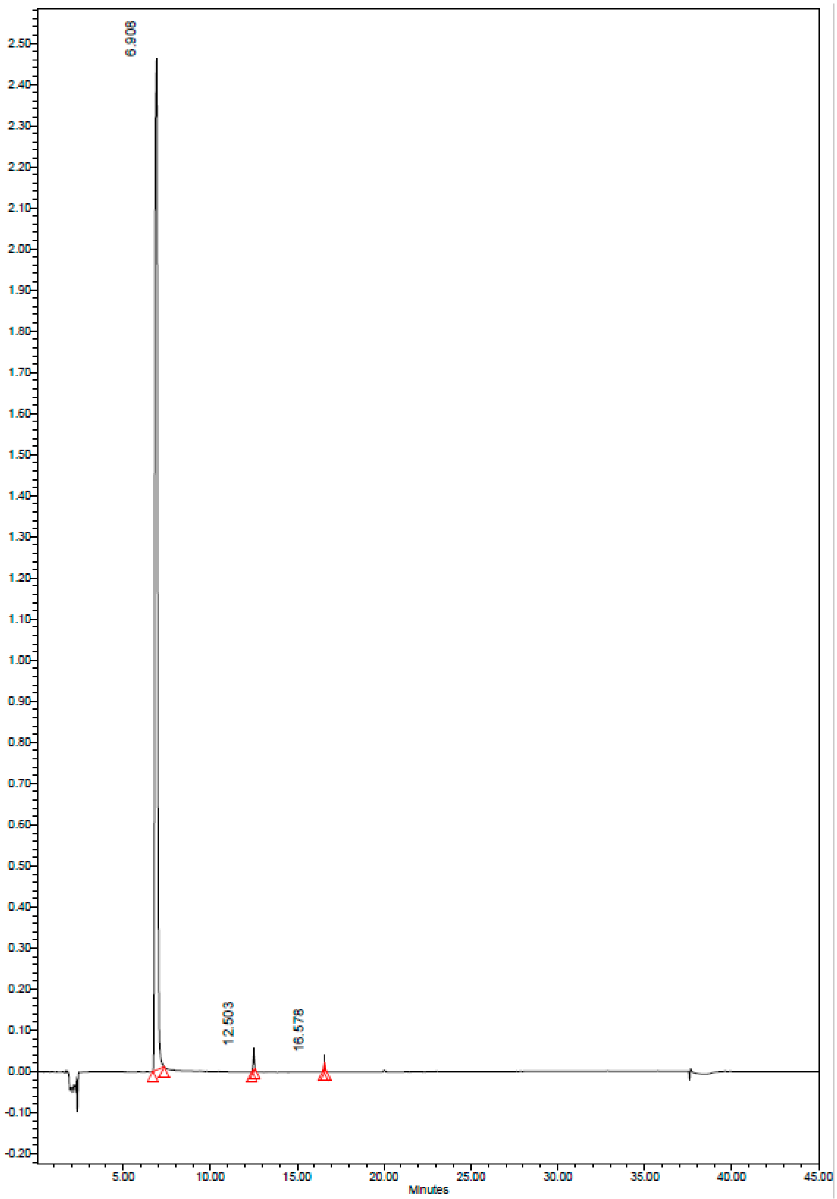

	Name	Retention Time (min)	Purity1 Angle	Purity1 Threshold	PDA Match1 Spect. Name	PDA Match1 Angle	PDA Match1 Threshold	PDA Match1 Lib. Name	Area (µV*sec)	% Area	Height (µV)	Int Type	Amount
1		6.908							26532338	98.94	2471560	bb	
2		12.503							192085	0.72	35261	bb	
3		16.578							91631	0.34	18319	bb	

WGU-V-19 102 (1.806)
1: TOF MS ES+

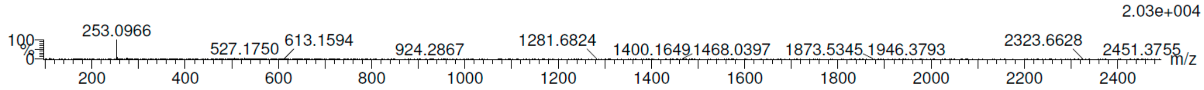

Minimum:				-1.5				
Maximum:		5.0	10.0	50.0				
Mass	Calc. Mass	mDa	PPM	DBE	i-FIT	Norm	Conf(%)	Formula
253.0966	253.0977	-1.1	-4.3	10.5	41.5	0.660	51.71	C15 H13 N2 O2
	253.0950	1.6	6.3	11.5	41.6	0.728	48.29	C11 H9 N8

Compound **3l** - (^{1}H NMR, ^{13} C NMR, HPLC, and HRMS data charts)

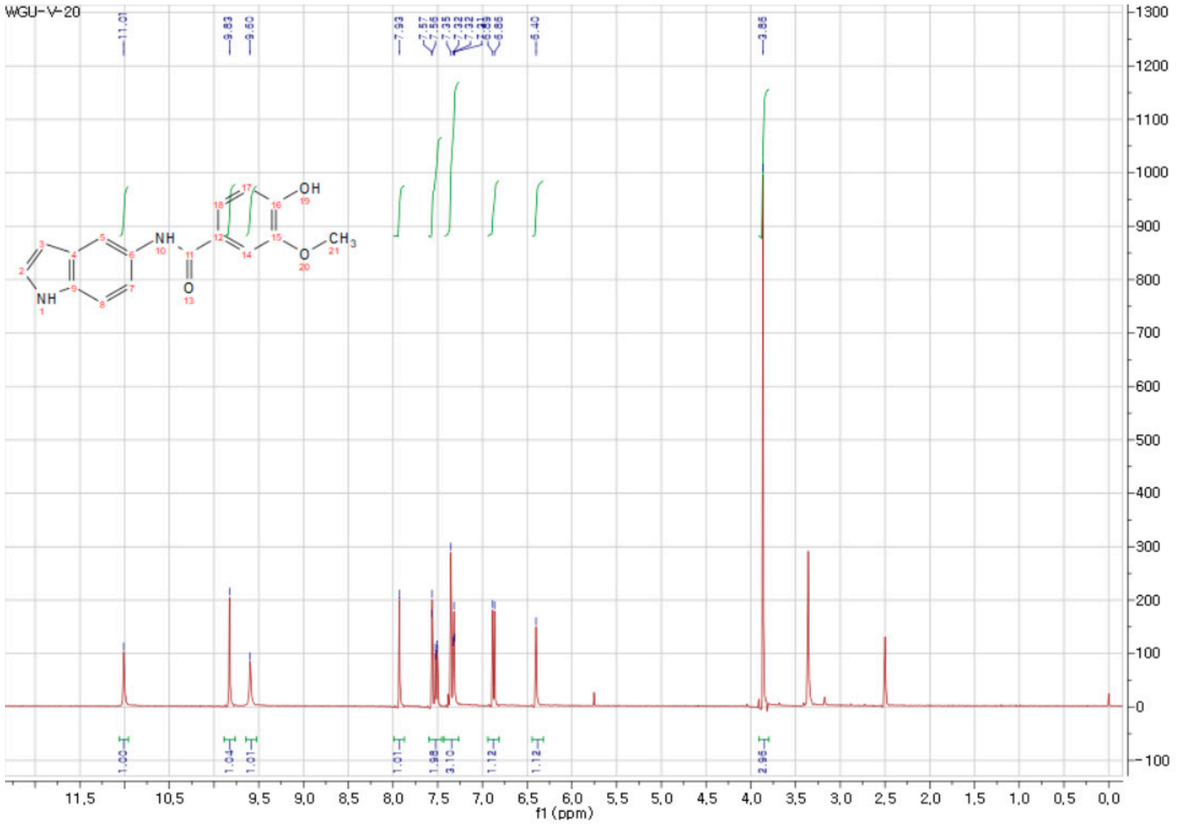

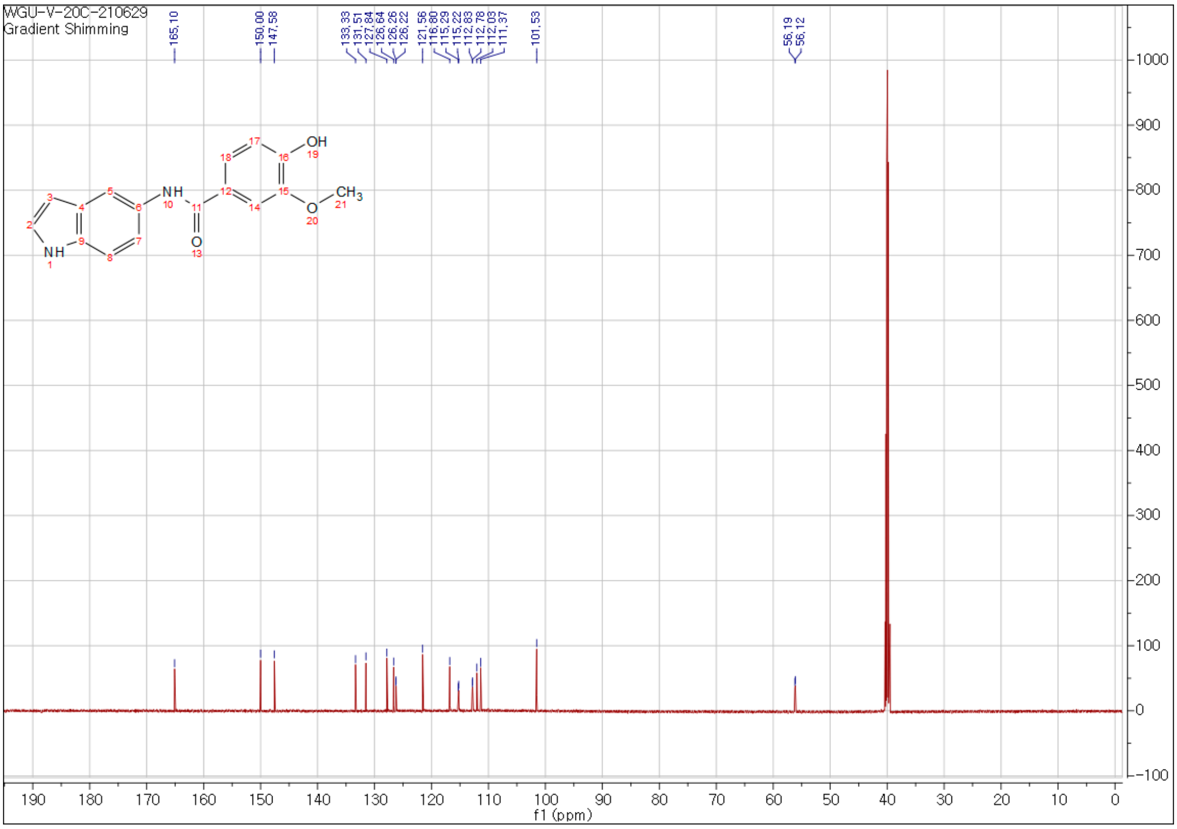

WGU-V-20C-210629
Gradient Shimming
165.10
150.00
147.58
133.33
131.51
127.84
126.64
126.26
126.22
121.56
116.80
115.29
115.22
112.83
112.78
112.03
111.37
101.53
56.19
56.12
f1 (ppm)

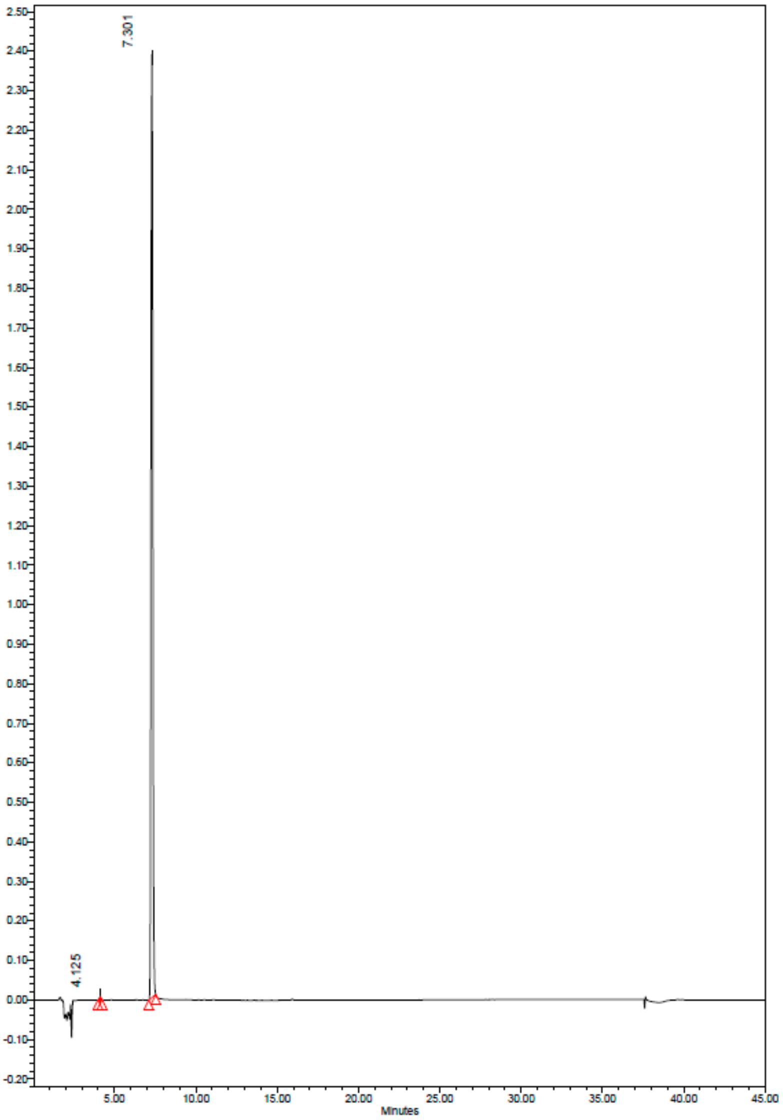

	Name	Retention Time (min)	Purity1 Angle	Purity1 Threshold	PDA Match1 Spect. Name	PDA Match1 Angle	PDA Match1 Threshold	PDA Match1 Lib. Name	Area (µV*sec)	% Area	Height (µV)	Int Type	Amount
1		4.125							48418	0.25	7272	bb	
2		7.301							18976963	99.75	2397016	bb	

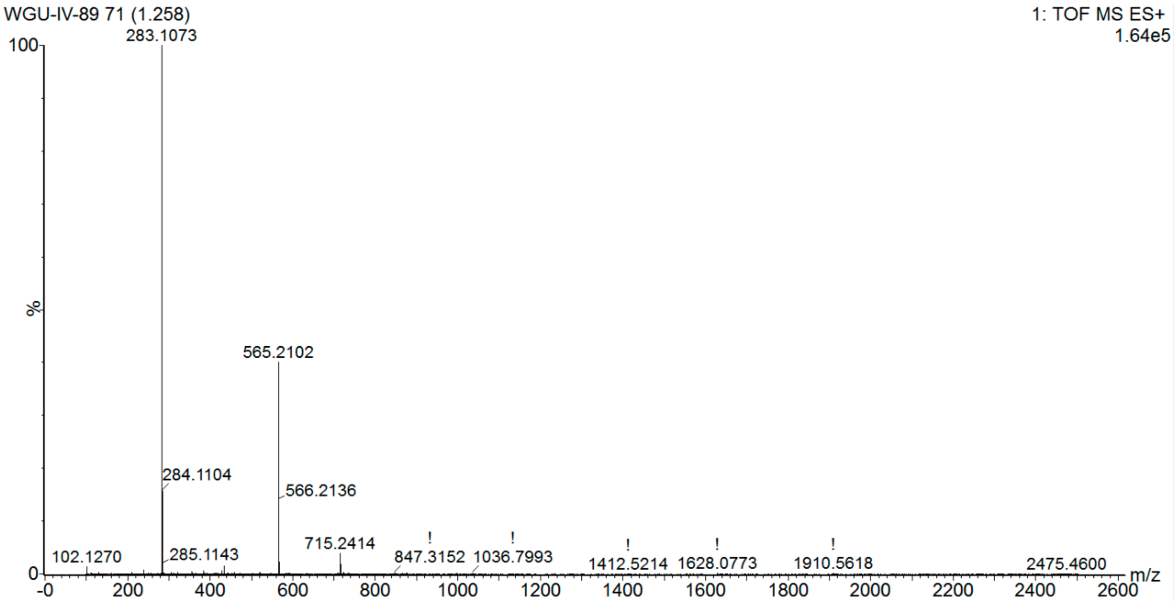

Monoisotopic Mass, Even Electron Ions
413 formula(e) evaluated with 7 results within limits (all results (up to 1000) for each mass)
Elements Used:
C: 0-500 H: 0-1000 N: 0-10 O: 0-20
WGU-IV-89 71 (1.258)
1: TOF MS ES+

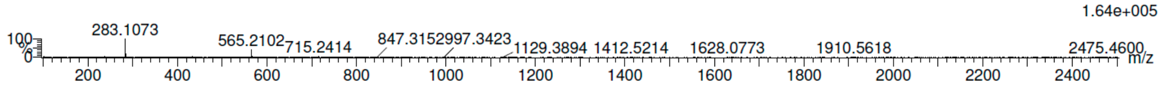

		mDa	PPM	DBE				
Minimum:				-1.5				
Maximum:		5.0	10.0	50.0				

Mass	Calc. Mass	mDa	PPM	DBE	i-FIT	Norm	Conf(%)	Formula
283.1073	283.1056	1.7	6.0	11.5	201.3	0.544	58.07	C12 H11 N8 O
	283.1083	-1.0	-3.5	10.5	202.0	1.277	27.88	C16 H15 N2 O3
	283.1042	3.1	10.9	6.5	202.8	2.065	12.69	C11 H15 N4 O5
	283.1123	-5.0	-17.7	14.5	205.2	4.492	1.12	C21 H15 O
	283.1029	4.4	15.5	1.5	206.9	6.114	0.22	C10 H19 O9
	283.1115	-4.2	-14.8	2.5	209.0	8.302	0.02	C5 H15 N8 O6
	283.1074	-0.1	-0.4	-1.5	213.3	12.549	0.00	H15 N10 O8

Compound **3m** - (^{1}H NMR, [13] C NMR, HPLC, and HRMS data charts)

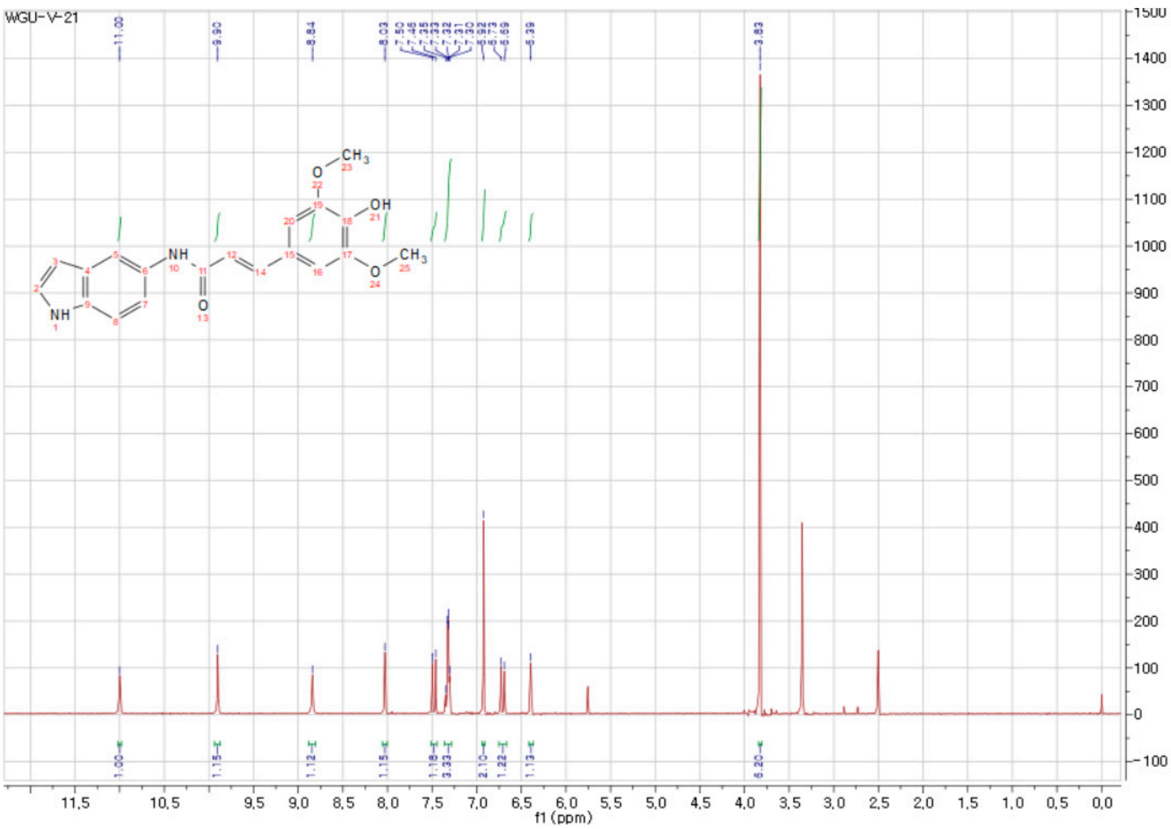

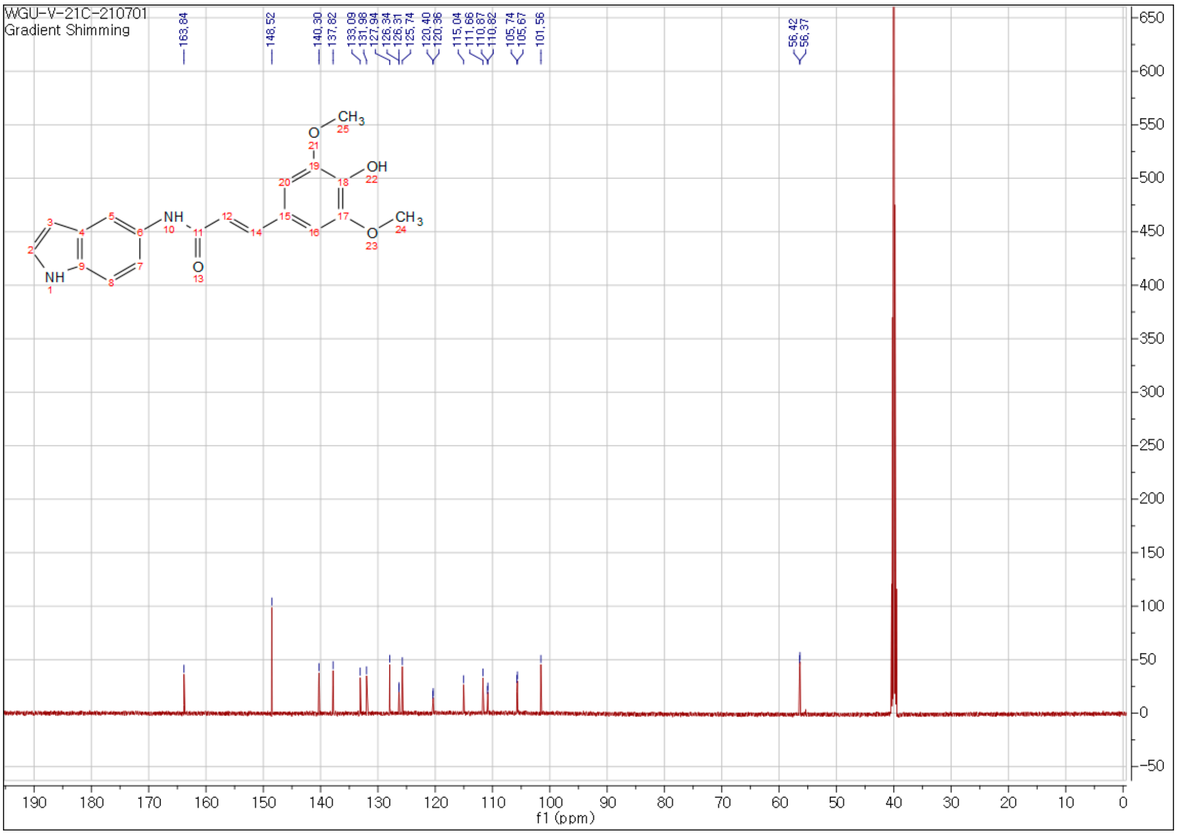

WGU-V-21C-210701
Gradient Shimming
163.84
148.52
140.30
137.82
133.09
131.98
127.94
126.34
126.31
125.74
120.40
120.36
115.04
111.66
110.87
110.82
105.74
105.67
101.56
56.42
56.37
f1 (ppm)

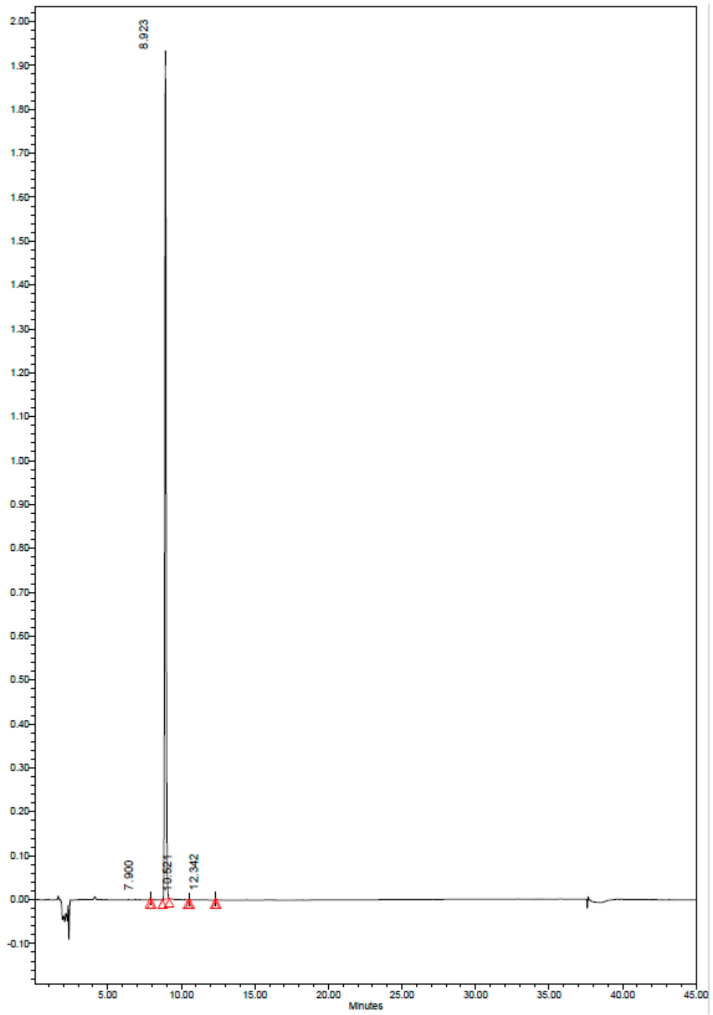

	Name	Retention Time (min)	Purity1 Angle	Purity1 Threshold	PDA Match1 Spect. Name	PDA Match1 Angle	PDA Match1 Threshold	PDA Match1 Lib. Name	Area (µV*sec)	% Area	Height (µV)	Int Type	Amount
1		7.900							8997	0.07	2039	bb	
2		8.923							12659576	99.88	1925922	bb	
3		10.521							1724	0.01	375	bb	
4		12.342							5023	0.04	1363	bb	

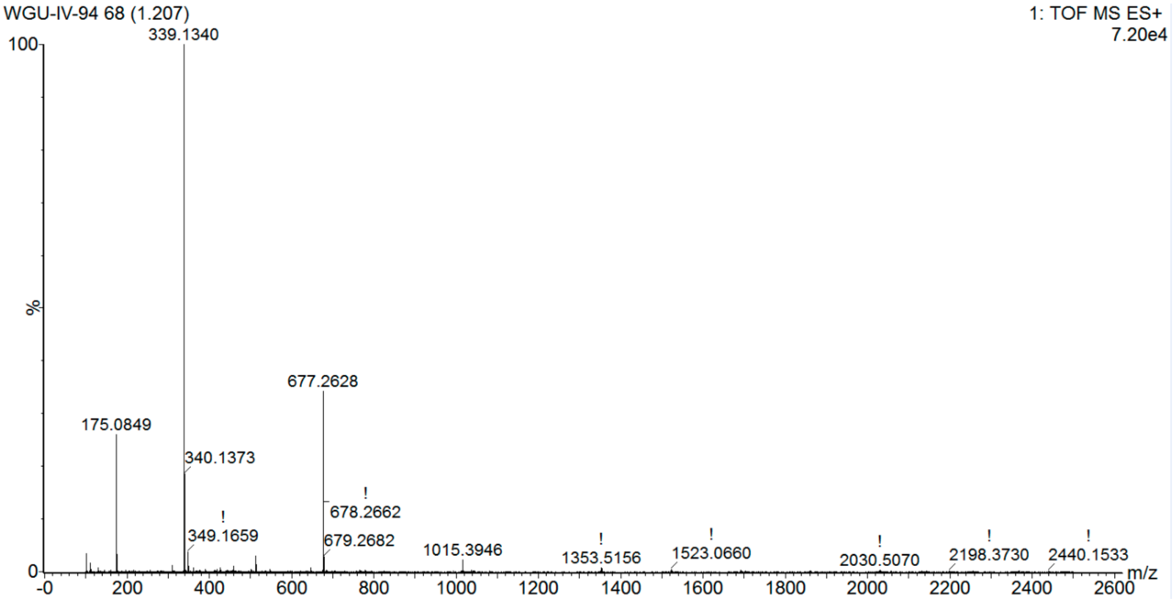

Monoisotopic Mass, Even Electron Ions
579 formula(e) evaluated with 9 results within limits (all results (up to 1000) for each mass)
Elements Used:
C: 0-500 H: 0-1000 N: 0-10 O: 0-20
WGU-IV-94 68 (1.207)
1: TOF MS ES+

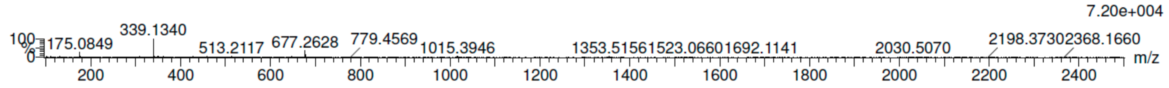

Mass	Calc. Mass	mDa	PPM	DBE	i-FIT	Norm	Conf(%)	Formula
Minimum:				-1.5				
Maximum:		5.0	10.0	50.0				
339.1340	339.1345	-0.5	-1.5	11.5	96.4	0.658	51.78	C19 H19 N2 O4
	339.1305	3.5	10.3	7.5	96.9	1.120	32.62	C14 H19 N4 O6
	339.1318	2.2	6.5	12.5	97.9	2.142	11.75	C15 H15 N8 O2
	339.1358	-1.8	-5.3	16.5	99.4	3.610	2.71	C20 H15 N6
	339.1385	-4.5	-13.3	15.5	101.0	5.182	0.56	C24 H19 O2
	339.1291	4.9	14.4	2.5	101.0	5.242	0.53	C13 H23 O10
	339.1377	-3.7	-10.9	3.5	103.9	8.146	0.03	C8 H19 N8 O7
	339.1363	-2.3	-6.8	-1.5	104.5	8.703	0.02	C7 H23 N4 O11
	339.1336	0.4	1.2	-0.5	107.1	11.361	0.00	C3 H19 N10 O9
